# Impact of Bisphenol A and its alternatives on oocyte health: a scoping review

**DOI:** 10.1093/humupd/dmae025

**Published:** 2024-09-14

**Authors:** Alexandra E Peters, Emmalee A Ford, Shaun D Roman, Elizabeth G Bromfield, Brett Nixon, Kirsty G Pringle, Jessie M Sutherland

**Affiliations:** School of Biomedical Science and Pharmacy, College of Health, Medicine, and Wellbeing, University of Newcastle, Callaghan, NSW, Australia; Mothers and Babies Research Program and Women's Health Research Program, Hunter Medical Research Institute, New Lambton Heights, NSW, Australia; School of Biomedical Science and Pharmacy, College of Health, Medicine, and Wellbeing, University of Newcastle, Callaghan, NSW, Australia; Mothers and Babies Research Program and Women's Health Research Program, Hunter Medical Research Institute, New Lambton Heights, NSW, Australia; The Research Centre, Family Planning Australia, Newington, NSW, Australia; Department of Research, NSW Health Pathology, Newcastle, NSW, Australia; Faculty of Science, School of BioSciences, Bio21 Institute, The University of Melbourne, Parkville, VIC, Australia; School of Environmental and Life Sciences, College of Engineering, Science, and Environment, University of Newcastle, Callaghan, NSW, Australia; Infertility and Reproduction Research Program, Hunter Medical Research Institute, New Lambton Heights, NSW, Australia; School of Environmental and Life Sciences, College of Engineering, Science, and Environment, University of Newcastle, Callaghan, NSW, Australia; Infertility and Reproduction Research Program, Hunter Medical Research Institute, New Lambton Heights, NSW, Australia; School of Biomedical Science and Pharmacy, College of Health, Medicine, and Wellbeing, University of Newcastle, Callaghan, NSW, Australia; Mothers and Babies Research Program and Women's Health Research Program, Hunter Medical Research Institute, New Lambton Heights, NSW, Australia; School of Biomedical Science and Pharmacy, College of Health, Medicine, and Wellbeing, University of Newcastle, Callaghan, NSW, Australia; Mothers and Babies Research Program and Women's Health Research Program, Hunter Medical Research Institute, New Lambton Heights, NSW, Australia

**Keywords:** oocyte quality, endocrine disrupting chemical, BPA, bisphenol, female fertility, reproductive toxicant, follicle development, oocyte maturation, chromosomal abnormalities

## Abstract

**BACKGROUND:**

Bisphenol A (BPA) is an endocrine disrupting chemical released from plastic materials, including food packaging and dental sealants, persisting in the environment and ubiquitously contaminating ecosystems and human populations. BPA can elicit an array of damaging health effects and, alarmingly, ‘BPA-free’ alternatives mirror these harmful effects. Bisphenol exposure can negatively impact female fertility, damaging both the ovary and oocytes therein. Such damage can diminish reproductive capacity, pregnancy success, and offspring health. Despite global government regulations in place to indicate ‘safe’ BPA exposure levels, these policies have not considered the effects of bisphenols on oocyte health.

**OBJECTIVE AND RATIONALE:**

This scoping review was conducted to evaluate evidence on the effects of BPA and BPA alternatives on standardized parameters of oocyte health. In doing so, this review addresses a critical gap in the literature providing a comprehensive, up-to-date synthesis of the effects of bisphenols on oocyte health.

**SEARCH METHODS:**

This scoping review was conducted in accordance with PRISMA guidelines. Four databases, Medline, Embase, Scopus, and Web of Science, were searched twice (23 February 2022 and 1 August 2023) to capture studies assessing mammalian oocyte health post-bisphenol exposure. Search terms regarding oocytes, ovarian follicles, and bisphenols were utilized to identify relevant studies. Manuscripts written in English and reporting the effect of any bisphenol on mammalian oocyte health from all years were included. Parameters for toxicological studies were evaluated, including the number of bisphenol concentrations/doses tested, dosing regimen, biological replicates and/or animal numbers, and statistical information (for human studies). Standardized parameters of oocyte health including follicle counts, oocyte yield, oocyte meiotic capacity, morphology of oocyte and cumulus cells, and oocyte meiotic spindle integrity were extracted across the studies.

**OUTCOMES:**

After screening 3147 studies, 107 studies of either humans or mammalian animal models or humans were included. Of the *in vitro* exposure studies, 96.3% (26/27) and 94.1% (16/17) found at least one adverse effect on oocyte health using BPA or BPA alternatives (including BHPF, BPAF, BPB, BPF, and BPS), respectively. These included increased meiotic cell cycle arrest, altered morphology, and abnormal meiotic spindle/chromosomal alignment. *In vivo*, 85.7% (30/35) of studies on BPA and 92.3% (12/13) on BPA alternatives documented adverse effects on follicle development, morphology, or spindle/chromosome alignment. Importantly, these effects were recorded using levels below those deemed ‘safe’ for human exposure. Over half (11/21) of all human observational studies showed associations between higher urinary BPA levels and reduced antral follicle counts or oocyte yield in IVF patients. Recommendations are presented based on the identified shortcomings of the current evidence, incorporating elements of FDA requirements for future research in the field.

**WIDER IMPLICATIONS:**

These data highlight the detrimental impacts of low-level BPA and BPA alternative exposure, contributing to poor oocyte quality and reduced fertility. These outcomes are valuable in promoting the revision of current policies and guidelines pertaining to BPA exposure internationally. This study serves as a valuable resource to scientists, providing key recommendations on study design, reporting elements, and endpoint measures to strengthen future studies. Ultimately, this review highlights oocyte health as a fundamentally important endpoint in reproductive toxicological studies, indicating an important direction for future research into endocrine disrupting chemicals to improve fertility outcomes.

## Graphical Abstract

**Figure:**
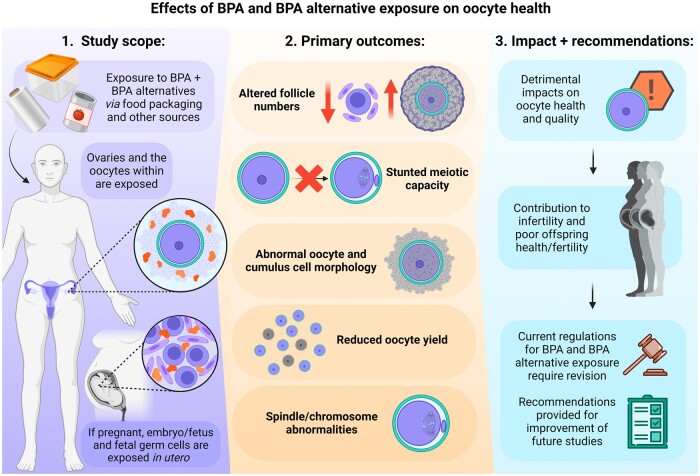
Exposure to BPA and BPA alternatives, at doses considered safe, has detrimental impacts on key oocyte health outcomes, impacting fertility success across generations. Created with BioRender.com.

## Introduction

Our food comes in contact with a multitude of materials during production, packaging, distribution, storage, and preparation. Food contact materials, such as food packaging in supermarkets, kitchenware, and reusable food and drink containers, are necessary to maintain food quality and safety. However, these materials can pose a significant contamination risk (Barnes *et al.*, 2006; Han *et al.*, 2018), whereby active chemical components within contact materials migrate into foods via diffusion (Barnes *et al.*, 2006; Muncke *et al.*, 2014). It is therefore imperative that these food contact materials are appropriately tested and regulated by manufacturers and government food safety bodies to ensure safety. Of growing concern, due to their established ability to impact human health, are endocrine disrupting chemicals in food packaging, such as Bisphenol A (BPA) and its chemical analogues (Vom Saal and Vandenberg, 2021).

BPA or 2,2-bis(4-hydroxyphenyl)propane is an industrial chemical that is widely used for the polymerization of plastics including polycarbonate reusable containers and kitchenware, epoxy resins lining metal cans, and some disposable polyvinyl chloride packaging. BPA is also used in non-food-related materials such as in thermal paper receipts, eyewear, and children’s toys (López-Cervantes and Paseiro-Losada, 2003; Metz, 2016; Almeida *et al.*, 2018; Vilarinho *et al.*, 2019). Used by manufacturers for over 70 years, BPA is well known for its diverse endocrine disrupting properties and ability to migrate into foods (Metz, 2016; Almeida *et al.*, 2018; Vilarinho *et al.*, 2019). The release of BPA as an active endocrine disruptor and its subsequent migration from plastic into foods is accelerated by exposure to common stressors including heating, microwave radiation, ultraviolet radiation, and repeated use (Yang *et al.*, 2011; Bertoli *et al.*, 2015). As such, >90% of human BPA exposure occurs through the oral route, with absorption occurring in the gastrointestinal tract (Vandenberg *et al.*, 2007). BPA is also a prevalent environmental contaminant, rampant within landfill and dispersed throughout ecosystems via microplastics, presenting further opportunities for contamination of food sources (Liu *et al.*, 2019; Vom Saal and Vandenberg, 2021; Makowska *et al.*, 2022). Ultimately, BPA causes an extensive range of adverse health effects in animal models including metabolic, cardiac, hepatic, neurological, reproductive, and developmental pathologies. Further, higher levels of urinary BPA are correlated with many of the same pathologies in humans, evidenced in over 100 observational studies (Vandenberg *et al.*, 2013a; Rancière *et al.*, 2015; Ejaredar *et al.*, 2017; Vom Saal and Vandenberg, 2021).

Within the past few decades, many BPA alternatives such as Bisphenol S (BPS), Bisphenol F (BPF), Bisphenol AF (BPAF), Bisphenol B (BPB), and Fluorene-9-Bisphenol (BHPF) have risen in popularity in response to consumer-driven BPA health concerns. These chemical analogues are consumed under a ‘BPA-free’ label carrying an assumption of safety, however the emerging literature on these compounds refutes these claims (Moon, 2019). Illustrative of this, the popular substitute BPS has higher oral availability than BPA, providing greater opportunity for exposure (Khmiri *et al.*, 2020). Consequently, there is a rapidly growing body of evidence in both animal and human studies showing that BPA alternatives can induce similar adverse health effects to BPA (Rochester and Bolden, 2015; Pelch *et al.*, 2019).

The mechanisms through which BPA exerts effects are pleiotropic and cell/tissue type specific, with their capacity to bind to estrogen receptors and other hormone receptors, disrupting hormone synthesis and epigenetic regulation (Wetherill *et al.*, 2007; Acconcia *et al.*, 2015). In studies where the activity of different BPA analogues has been compared to BPA, many similar or more potent effects have been demonstrated. For example, BPAF and BPB have stronger agonistic effects on human estrogen receptor β than that elicited by BPA (Kojima *et al.*, 2019). Importantly, it is well-documented that bisphenols can have a non-monotonic dose response, that is, a non-linear relationship between dose and effect, complicating the assessment of resultant adverse health effects (Vandenberg, 2014; Eladak *et al.*, 2015). Causing even greater complications are the combined effects of bisphenols with each other or other endocrine disrupting chemicals, which is most reflective of real-life exposure, and results in further alterations of the dose-response dynamics (Hamid *et al.*, 2021).

Female fertility and specifically oocyte health is one aspect of human health that is particularly vulnerable to endocrine disrupting chemicals such as BPA and other bisphenols (Hunt *et al.*, 2003). This is attributed to the finite and long-lived nature of oocytes, remaining arrested in meiosis within the ovary for many decades (Telfer and McLaughlin, 2007; Inoue *et al.*, 2008; Peters *et al.*, 2020). Over this protracted period of arrest, the pre-ovulatory oocyte is susceptible to a variety of exposures from lifestyle or environmental sources that can compromise oocyte quality and function (Muncke *et al.*, 2014; Peters *et al.*, 2020). Indeed, resultant oocyte damage can impair the oocyte’s ability to mature and fertilize optimally, impacting fertility with long-term consequences for embryo development, fetal health, pregnancy success and, ultimately, the health of future generations (Krisher, 2013).

In 2008, the US Food and Drug Administration (FDA) concluded the BPA no observed adverse effect level (NOAEL) as 5 mg/kg bw/day based on the findings of two multigenerational rodent studies funded by the plastic industry (Tyl *et al.*, 2002, 2008; FDA BPA Joint Emerging Science Working Group, 2014b). Importantly, the NOAEL informs the tolerable daily intake (TDI), that is, the estimated amount of a contaminant set by food safety authorities that can be consumed over a lifetime without an appreciable health risk. For BPA, a TDI of 0.05 mg/kg bw/day was adopted in the USA, Australia, and New Zealand in 2010, joining Japan, South Korea, and the European Union with the same TDI regulations (Food Standards Australia New Zealand, 2010; Almeida *et al.*, 2018). During this same period, there was a consumer-driven ban of BPA from baby bottles and infant formula packaging across most developed countries (Food Standards Australia New Zealand, 2010; European Commission, 2011; Food and Drug Administration, 2012). More recently, in April 2023, the European Food Safety Authority (European FSA) established a newly lowered TDI of 0.2 ng/kg bw/day due to health concerns over low-level dietary BPA exposure. France has also banned BPA in almost all food contact materials since 2015 (Eladak *et al.*, 2015; Lambré *et al.*, 2023).

There is limited information available regarding the safe exposure limits of BPA alternatives worldwide. This is despite a growing number of these alternatives being identified as reproductive and developmental toxicants and suspected endocrine disruptors (NICNAS, 2015; Kojima *et al.*, 2019; EFSA *et al.*, 2020). For BPS, a popular alternative to BPA, the European FSA claims BPS does not affect reproductive performance at extremely high doses up to 180 mg/kg bw/day in rodents (EFSA *et al.*, 2020). Similarly, both the Australian National Industrial Chemicals Notification and Assessment Scheme and United States Environmental Protection Agency (US EPA) identifies the reproductive NOAEL for BPS reproductive toxicity at high doses of 60 mg/kg bw/day (EPA. 2014; NICNAS, 2019). In line with these high NOAELs, BPA alternatives are minimally regulated in food packaging globally. Some regulations in place for popular alternatives like BPS include migration limits from packaging into food at 0.05 mg/kg in the European Union, and efforts to limit use in children’s products in three US states (EPA. 2014; EFSA *et al.*, 2020).

Concerningly, many of the current guidelines for ‘safe’ BPA exposure to food do not consider the effects of BPA on oocyte health and fertility. The aforementioned US FDA-led studies reported minimal primordial follicle count data and nothing at all to assess oocyte health. Furthermore, the use of ‘safer’ alternatives to BPA, including BPS, in food packaging is increasing, with little toxicological investigation or tailored restrictions in place. This lack of regulation and independent safety testing, especially given the history of BPA, is extremely concerning. Herein, we used a scoping review to synthesize the peer-reviewed literature assessing the effects of BPA and BPA alternatives on oocyte health parameters. Through this, we aimed to ascertain what is known within the field to date, to highlight what information is lacking or insufficient, and to determine whether the current restrictions in place for BPA and its alternatives are appropriate for protecting oocyte health. Ultimately, this scoping review highlights oocyte health as an important measure of fertility for toxicological studies, informs new or modified guidelines for both currently unregulated and regulated substances, and finally, provides best practice recommendations for future studies in this field.

## Methods

The format of a scoping review was selected for this study in recognition that scoping reviews provide a powerful platform from which to synthesize literature from a range of study designs incorporating diverse data such as different exposures and different measures of oocyte health (Munn *et al.*, 2018).

This scoping review adheres to the PRISMA extension for scoping reviews (Tricco *et al.*, 2018); no protocol is registered.

### Study criteria

To initially be eligible for inclusion in this review, a study had to assess the effects of a toxicant resulting from food processing or packaging on parameters related to oocyte health. Such oocyte health parameters included any direct measure of oocyte health including morphology, spindle alignment, cellular markers of health, e.g. apoptosis or autophagy, oxidative stress, inflammation, chromatin modification, capacity to mature *in vitro*, etc. Studies utilizing oocytes of mammalian origin, either using animal models or human samples, from all years were included. These studies included *in vitro*, *in vivo*, and human observational assessments with acute or chronic exposures of contaminant or toxicant chemicals. In addition, *in vivo* studies that assessed multigenerational or transgenerational effects resulting from prenatal, perinatal, or postnatal exposures were included. Studies focussing on any valid toxicant/food contaminant that is recognized as a possible risk (as determined by the US FDA, Food Standards Australia New Zealand, European FSA, or otherwise) within the western diet were included. Studies assessing toxicant impacts on non-mammalian species such as fish and insects were excluded. Studies in languages other than English were excluded. Systematic/scoping and narrative reviews, case studies, editorials, conference abstracts, and grey literature (non-academic publishing) were excluded.

### Search strategy

Four databases (Medline, Embase, Scopus, and Web of science) were searched using relevant search terms on the 23rd of February 2022. The search terms were constructed to identify a broad range of studies assessing the effects of food contaminants and toxicants on oocyte health. These were designed through consulting the US FDA, Food Standard Australia New Zealand, and European FSA legislations on food chemical safety, to target the most commonly recognized substances within the modern western diet. Search terms and operators were modified according to the database requirements. Additional sources found within reference sections of key studies or government review documents were manually examined by A.E.P to determine if they met the inclusion criteria for this review.

An example of the search in Medline includes: (oocyte.mp. or Oocytes/or pre-ovulatory.mp. or germinal vesicle.mp.) AND (toxicant*.mp. or Food Contamination/or food contamin*.mp. or (food* adj3 packag*).mp. or Food Packaging/or food contact material.mp. or food processing.mp. or Plasticizers/or plastici? er*.mp. or Phthalic Acids/or phthalate.mp. or Microplastics/or microplastic*.mp. or (leachate* adj3 plastic*).mp. or bisphenol.mp. or Endocrine Disruptors/or Styrenes/or styrene*.mp. or plastic*.mp. or Plastics/or (plastic* adj3 additive*).mp. or Vinyl Chloride/or acrylamide*.mp. or chloropropanol*.mp. or 3-MCPD.mp. or glycidyl ester*.mp. or 4-methylimidazole*.mp. or ethyl carbamate*.mp. or Furans/or furan*.mp. or Polycyclic Aromatic Hydrocarbons/or heterocyclic aromatic amine.mp. or Nitrosamines/or nitrosamine*.mp.).

A second search was conducted on the 1st of August 2023 to capture additional literature added to the four databases since the previous search. As this search was conducted after bisphenols were chosen as the focus of this study, the search terms were modified to only capture studies assessing the impacts of bisphenols on oocyte health. This search was also expanded to include any studies that may not have included the term ‘oocyte’ but did examine ovarian follicles in line with follicle counts being a key study parameter recorded in this review.

An example of the second search in Medline includes: ((Ovary/or ovar*.mp.) AND (Ovarian Follicle/or follic*.mp.) or oocyte.mp. or Oocytes/or oocytes.mp. or pre-ovulatory.mp. or germinal vesicle.mp.) AND bisphenol*.mp. limit to dt = 2022023-20230801.

### Screening

After the removal of duplicates, two reviewers (A.E.P. and E.A.F.) independently conducted title and abstract screening of the search results in Covidence using the defined inclusion criteria. Any discrepancies in screening results were resolved through discussion. Studies that passed title and abstract screening were then obtained and subject to full-text review by A.E.P, and exclusions were confirmed by an independent reviewer (J.M.S.). Full texts that met this inclusion criteria and included the assessment of BPA or BPA alternatives were chosen as the focus of this study and proceeded to the data extraction stage.

### Data extraction

Data were extracted by A.E.P. who collected the following information: author/s, year of publication, bisphenol studied, study design, dose and administration of bisphenol, detected bisphenol concentration of participant samples (for human studies), test species and age, location (for human studies), sample size, primary aim, the standardized oocyte health parameters measured, and their outcomes. These standardized oocyte health parameters were a set of five parameters chosen based on the US EPA guidelines for reproductive toxicity and standard observable/morphological measures of oocyte health used within Australian IVF clinics, and internationally applicable (EPA, 1996; Rienzi *et al.*, 2012). These parameters were also the most consistently reported across the included studies regardless of their primary aim. These include follicle counts, oocyte yield, oocyte meiotic capacity (ability for the oocyte to progress through meiosis up until MII arrest), morphology of the oocyte and cumulus cells, and oocyte meiotic spindle integrity. Other cellular indicators of oocyte/ovarian health that were measured in a proportion of included studies, such as oxidative stress, apoptosis, or epigenetic changes, were not data extracted as part this review. Although study quality is typically not assessed in scoping reviews (Tricco *et al.*, 2018), aspects of study quality pertaining to toxicological study design were extracted, including the number of bisphenol concentrations/doses assessed in each study, the reporting of animal numbers within a dosing regime, and whether bisphenol delivery was oral. Extracted data was reviewed for completeness and accuracy by J.M.S. The results of this review are reported as a narrative synthesis.

## Results

### Search results

**Figure 1.**
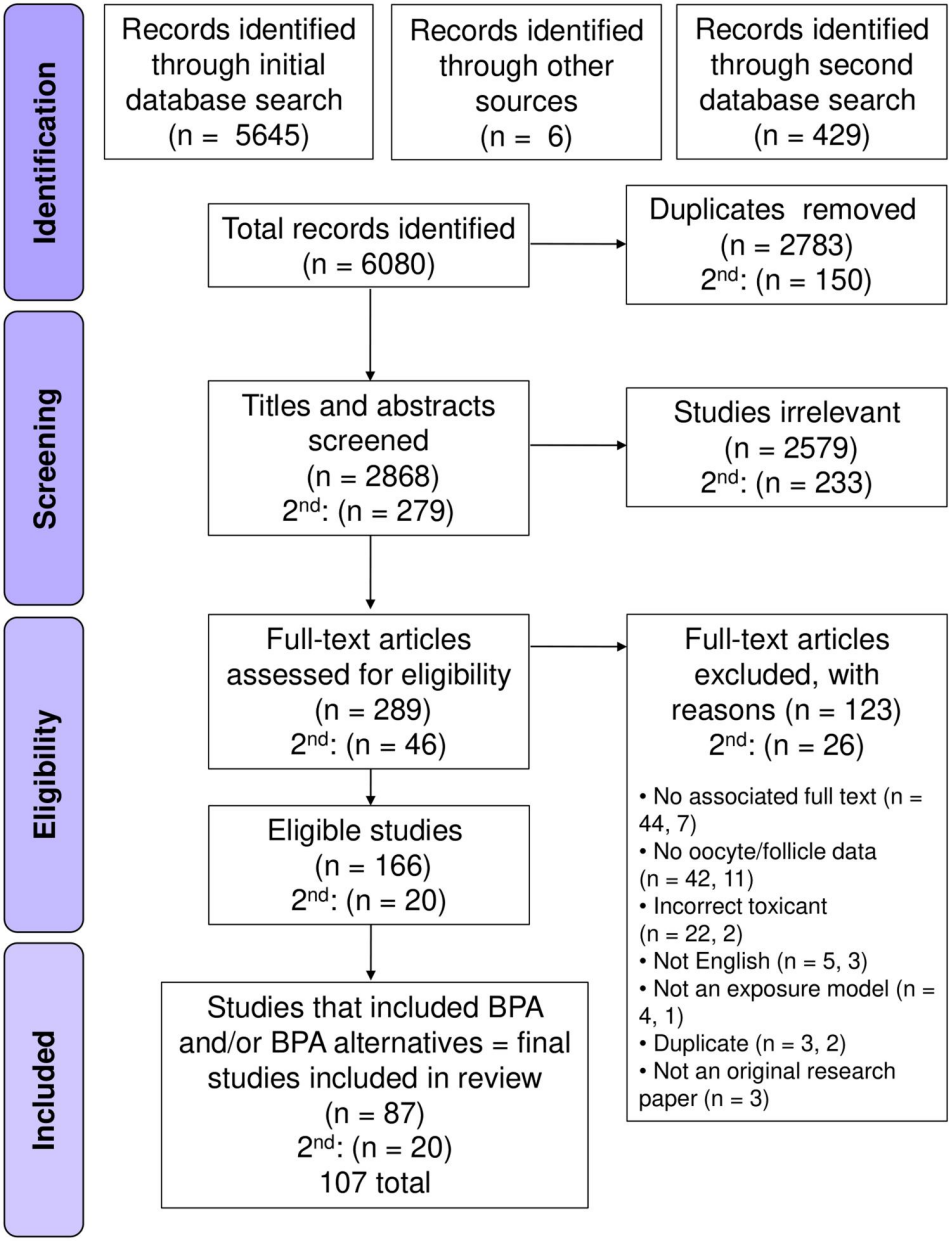
PRISMA flowchart for the selection of studies assessing dietary toxicants and bisphenols on oocyte health and female fertility.

Searching across four databases and additional sources initially yielded 5651 studies, with an additional 429 identified in the second ‘bisphenol only’ search (Fig. 1). Following the removal of duplicates, 3147 studies remained, with title and abstract screening as per the inclusion criteria reducing the total eligible for full text review to 335. To be included in this review, a study had to assess the effects of a toxicant resulting from food processing or packaging on mammalian oocyte health parameters. At full text review, 149 studies were excluded for not appropriately addressing the criteria, with most common reasons including: no published full text associated with abstract, not assessing parameters directly related to the oocyte or follicle, or the toxicant assessed was not found to be associated with food processing or packaging. Four additional studies found in US FDA review documents were manually added at full text review. At the conclusion of full text assessment, 166 studies (along with 20 from the second search) were suitable for inclusion in this review (Fig. 1).

### Dietary toxicants arising from food processing and packaging in oocyte health literature

**Figure 2.**
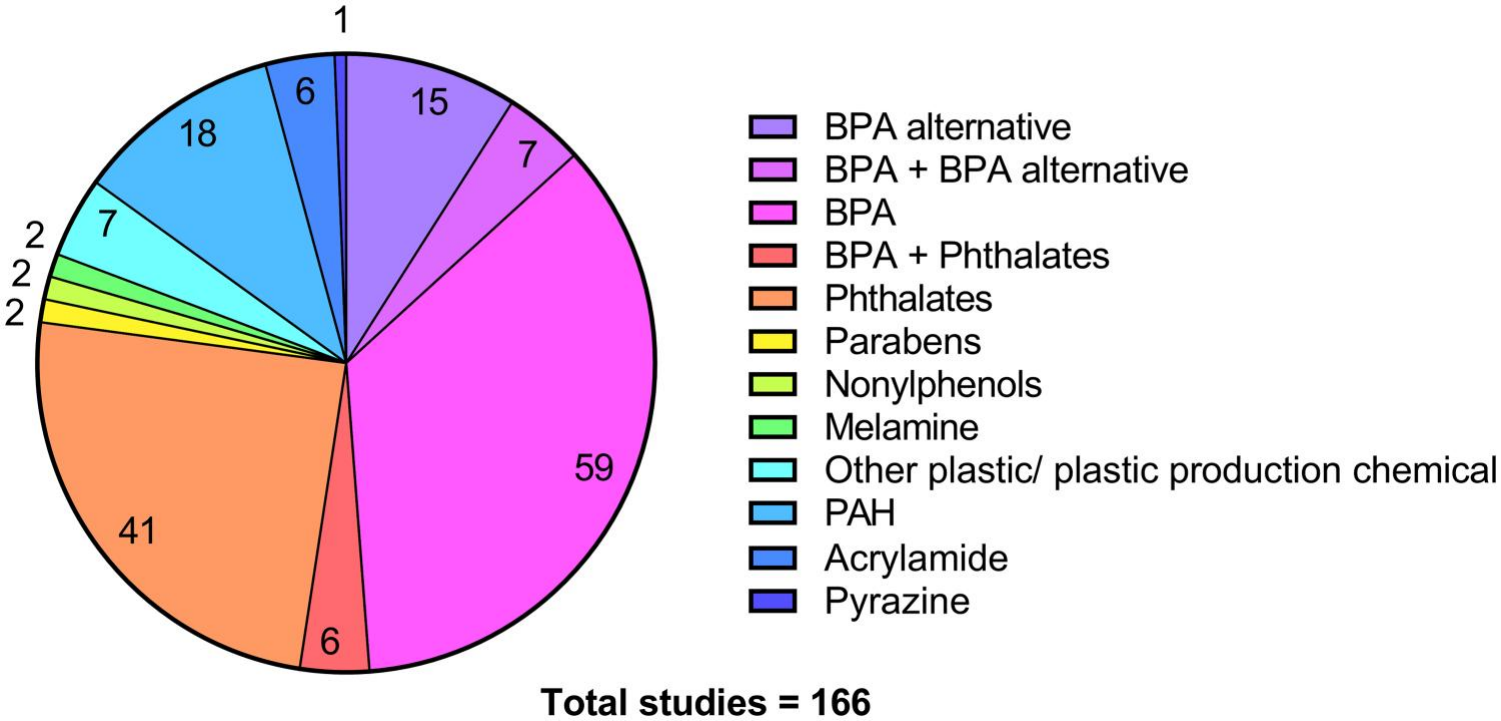
**Pie chart presenting 166 studies included after initial full-text review, categorized by the type of toxicant they assessed.** Studies that included more than one toxicant were presented in a separate category. Colour key corresponds to each category, and the number of studies in each category is represented on the chart.

The 166 studies that initially met our inclusion criteria addressed a wide variety of toxicants arising from food processing and packaging, grouped accordingly in Fig. 2. More than half of these studies (52.4%, 87) investigated the impact of BPA and/or alternatives of BPA on oocyte health. To enable a more comprehensive assessment of these studies, the focus of this review is on these 87 studies, plus the 20 additional studies from the second search. These studies underwent data extraction with their study design characteristics and aims being detailed in Table 1.

**Table 1.** Summary of key features of 107 included studies.

Title	Authors (year)	Primary aim	Toxicant/s of interest studied	Exposure/type of study	Concentration/dose and administration regime OR detected levels	Female species/strain and age at collection	Sample size (relevant to outcomes of interest)
**STUDIES THAT ASSESSED BPA ALTERNATIVES**							

Foetal exposure to the bisphenols BADGE and BPAF impairs meiosis through DNA oxidation in mouse ovaries	Abdallah *et al.* (2023)	To explore the effects of prenatal exposure of mouse oocytes to BADGE and BPAF on meiosis initiation and progression and their consequences on fertility in adulthood.	BADGE and BPAF	*In vivo* (*in utero*)	Delivered orally to pregnant mothers in drinking water at ∼500 µg/kg bw/day for 5 or 9 days from 10.5–14.5 or 18.5 dpc	NMRI and OGG1-deficient C57/Bl6 mice, 18.5 dpc or PND8, or 3 months old	Total animals in each treatment group not specified, 3–5 independent exposures per experiment
Spindle abnormalities and chromosome misalignment in bovine oocytes after exposure to low doses of bisphenol A or bisphenol S	Campen *et al.* (2018)	To compare the effects of *in vitro* exposure to BPA or BPS on meiotic progression, spindle morphology and chromosome alignment in bovine oocytes.	BPA and BPS	*In vitro*	COCs treated with 1, 10, 100 fM, pM, nM, or 50 µM for 24 h	Holstein-Friesian and Jersey heifers (cows), naturally cycling	≥3 independent replicates per experiment, total 18–250 oocytes per treatment group
Bisphenol S impaired *in vitro* ovine early developmental oocyte competence	Desmarchais *et al*. (2020)	To examine the acute effects of low and environmental doses of BPS on ewe oocyte quality and developmental competence, and its mechanism of action during *in vitro* maturation.	BPS	*In vitro*	COCs treated with 1 nM, 10 nM, 100 nM, 1 µM or 10 µM for 24 h	Adult ewes (sheep), reproductive age	≥3 independent replicates per experiment, 30–60 COCs per treatment group per replicate
Chronic low BPS exposure through diet impairs *in vitro* embryo production parameters according to metabolic status in the ewe	Desmarchais *et al.* (2022)	To determine BPS *in vivo* effects on folliculogenesis and embryo production after chronic exposure through diet, and the influence of metabolic status in adult ewes.	BPS	*In vivo*	Delivered orally in diet at 4 or 50 µg/kg bw/day) for at least 3 months	Ile-de-France ewes (sheep), ∼2.5 years old, primiparous	10 animals per treatment group
Bisphenol AF negatively affects oocyte maturation of mouse *in vitro* through increasing oxidative stress and DNA damage	Ding *et al.* (2017)	To investigate the toxic effects of BPAF on mouse oocytes and its possible mechanisms.	BPAF	*In vitro*	GV oocytes treated with 50, 100 or 200 μM for 0, 8, or 14 h	Kunming mice, 3–4 weeks old	≥3 independent replicates per experiment, cell numbers not provided
Bisphenol F exposure affects mouse oocyte *in vitro* maturation through inducing oxidative stress and DNA damage	Ding *et al.* (2022)	To study BPF toxicity on mouse oocyte meiotic maturation and quality.	BPA and BPF	*In vitro*	GV oocytes treated with 100, 200, 250, or 300 µM BPA, or 300, 400, or 500 µM BPF for 8 or 14 h	Kunming mice, 3–4 weeks old	≥3 biological replicates per experiment, total ∼90–200 oocytes per treatment group
Fluorene-9-bisphenol exposure induces cytotoxicity in mouse oocytes and causes ovarian damage	Jia *et al.* (2019)	To investigate the toxicity and mechanism of BHPF exposure in mouse oocytes *in vitro* and *in vivo*.	BHPF	*In vitro* and *in vivo*	***In vitro*:** GVs treated with 50, 100, 200, or 400 µM for 14 h ***In vivo*:** Delivered via gastric cannula in peanut oil at 2, 10, or 50 mg/kg bw/day for 12 days	CD-1 mice, minimum 8 weeks old	Independent replicates, cell numbers, or animal numbers not provided
Effects of acute fluorene-9-bisphenol exposure on mouse oocyte *in vitro* maturation and its possible mechanisms	Jiao *et al.* (2019)	To evaluate the effects and its related mechanisms of BHPF on mouse oocyte maturation *in vitro*.	BHPF	*In vitro*	GV oocytes treated with 50, 100, or 150 µM for 2 or 12 h	Kunming mice, age not specified	≥3 independent replicates per experiment, total 27–174 oocytes per treatment group
The toxic effects of fluorene-9-bisphenol on porcine oocyte *in vitro* maturation	Jiao *et al.* (2020)	To evaluate the potential effects of BHPF on porcine oocyte maturation and quality derived from COCs *in vitro*.	BHPF	*In vitro*	COCs treated with 25, 50, or 75 μM for 42–44 h	Large white pigs, pre-pubertal	≥3 independent replicates per experiment, total 61–140 oocytes per treatment group
Use of a mouse model of experimentally induced endometriosis to evaluate and compare the effects of Bisphenol A and Bisphenol AF exposure	Jones *et al.* (2018)	To determine if BPA or BPAF potentiate the development of endometriosis and if hormonal status alters how toxicant exposure affects disease.	BPA and BPAF	*In vivo*	Delivered orally in diet at 0.001 mg/kg bw/day, 5 mg/kg/day (BPA NOAEL) or 50 mg/kg bw/day (BPA LOAEL) for 7 weeks	Transgenic C57BL/6-Tg(UBC-GFP)30Scha/J (GFP) and wild type C57BL/6J mice, sexually mature	≥6 animals per treatment group
Cumulative and potential synergistic effects of seven different bisphenols on human granulosa cells *in vitro*?	Lebachelier de la Riviere *et al.* (2023)	To investigate the *in vitro* effects of six BPA analogues and BPA on human granulosa cell characteristics and hormonal features.	BPA, BPS, BPF, and BPAF	Human observational + *in vitro* (*in vitro* not relevant)	Mean: BPA: 0.075 ng/ml, BPS: 0.21 ng/ml, BPF: 0.13 ng/ml, BPAF: not detected	Women undergoing ART procedure, 17–43 years old, France	277 participants, follicular fluid collected at time of oocyte retrieval
Detrimental effect of Bisphenol S in mouse germ cell cyst breakdown and primordial follicle assembly	Liu *et al.* (2021)	To assess the effect of BPS on early ovarian folliculogenesis in mice.	BPS	*In vitro* and *in vivo*	***In vitro*:** PND0 ovaries treated with 10, 50, or 100 μM for 3 days ***In vivo*:** Delivered intraperitoneally to newborn mice in saline solution at 2 or 10 mg/kg bw/day for 3 days	CD-1 mice, collected at 0 (*in vitro*), 3 or 21 (*in vivo*) days old	***In vitro*:** ≥3 replicates per experiment, ovary numbers not provided ***In vivo*:** Animal numbers per treatment group not provided, ≥3 replicates per experiment
Comparison of the effects of BPA and BPAF on oocyte spindle assembly and polar body release in mice	Nakano *et al.* (2016)	To elucidate the effects of BPA and BPAF on oocyte maturation by monitoring maturation and MAD2 localization in oocytes cultured using the hanging drop method.	BPA and BPAF	*In vitro*	COCs treated with 2, 20, 50 or 100 µg/ml for 18 h or with 50 µg/ml for 12 h with 9 h recovery	ICR mice, 3–4 weeks old	Three replicates per experiment, ≥30 oocytes per replicate
Long-term exposure to very low doses of Bisphenol S affects female reproduction	Nevoral *et al.* (2018)	To evaluate the effect of BPS on folliculogenesis and oocyte quality after *in vivo* exposure to low doses of BPS.	BPS	*In vivo*	Delivered orally to 4-week-old mice in drinking water at 0.001, 0.1, 10, or 100 µg/kg bw/day for 4 weeks	ICR mice, collected at ∼8 weeks old	16 animals per treatment group (3 per group for oocyte data, 7 per group for ovary data)
Exposure to alternative bisphenols BPS and BPF through breast milk: noxious heritage effect during nursing associated with idiopathic infertility	Nevoral *et al.* (2021)	To simulate the real-life exposure route to bisphenols being considered safe for reproductive health.	BPS and BPF	*In vivo* (through breast milk)	Delivered orally in drinking water to dams at 0.2 or 20 µg/kg bw/day during their nursing period between offspring age PND0–15	ICR mice, collected at PND15 or PND60	4–9 breastfeeding dams and 3–16 offspring per treatment group
Effects of BPA, BPS, and BPF on oxidative stress and antioxidant enzyme expression in bovine oocytes and spermatozoa	Nguyen *et al.* (2022)	To confirm the effects of BPA on oxidative stress levels in gametes and investigate whether BPS and BPF affect oocytes and sperm by increasing oxidative stress.	BPF	*In vitro*	COCs treated with 0.05 mg/ml for 24 h	*Bos taurus* (cow), age not specified	≥3 biological replicates per experiment, ≥10 COCs per treatment group per replicate
Effects of Bisphenol-S low concentrations on oxidative stress status and *in vitro* fertilization potential in mature female mice	Nourian *et al.* (2017)	To elucidate the dose-dependent effects of BPS exposure on *in vitro* fertilization outcome and oxidative stress status using mice as an animal model.	BPS	*In vivo*	Delivered intraperitoneally to 2–3-month-old mice at 1, 5, 10, 50, or 100 µg/kg bw/day for 21 days	Mice (strain not specified), collected at ∼3–4 months old	Five animals per treatment group
BPS‐induced ovarian dysfunction: protective actions of melatonin via modulation of SIRT‐1/Nrf2/NFĸB and IR/PI3K/pAkt/GLUT‐4 expressions in adult golden hamster	Pal *et al.* (2023)	To investigate molecular BPS-induced ovarian toxic injuries and their remedy by melatonin.	BPS	*In vivo*	Delivered orally in corn oil at 150 mg/kg bw/day for 28 days	Golden hamsters, adult	12 animals per treatment group (4 per group for ovarian histology)
Acute low-dose Bisphenol S exposure affects mouse oocyte quality	Prokešová *et al.* (2020)	To assess cytoskeletal and chromatin changes in oocytes following *in vivo* exposure, using appropriate markers.	BPS	*In vivo*	Delivered orally to ∼2 month old mice in DMSO at 0.001, 0.1, 10, or 100 ng/g bw/day for 7 days	ICR mice, collected at ∼2 months old	15 animals per treatment group
BPA and BPS affect Connexin 37 in bovine cumulus cells	Sabry *et al.* (2021a)	To address the effects of BPA and BPS on Connexin 43 and 37 during *in vitro* maturation of bovine COCs and *in vitro* culture of bovine cumulus cells.	BPA and BPS	*In vitro*	COCs treated with 0.05 mg/ml for 24 h	*Bos taurus* (cow), age not specified	≥3 biological replicates per experiment, COC treatment numbers not provided
Effects of Bisphenol A and Bisphenol S on microRNA expression during bovine (*Bos taurus*) oocyte maturation and early embryo development	Sabry *et al.* (2021b)	To test if abnormal expression of key miRNAs during oocyte maturation and embryo development occurs following BPA and BPS exposure during maturation.	BPA and BPS	*In vitro*	COCs treated with 0.05 mg/ml for 24 h	*Bos taurus* (cow), age not specified	≥3 biological replicates per experiment, COC treatment numbers not provided
BPA and BPS affect the expression of anti-Mullerian hormone (AMH) and its receptor during bovine oocyte maturation and early embryo development	Saleh *et al.* (2021)	To investigate the effects of BPA and BPS on embryo developmental capability.	BPA and BPS	*In vitro*	COCs treated with 0.05 mg/ml for 24 h	*Bos taurus* (cow), age not specified	≥3 biological replicates per experiment, 60 COCs per treatment group
Bisphenol S impairs oestradiol secretion during *in vitro* basal folliculogenesis in a mono-ovulatory species model	Vignault *et al.* (2022)	To study BPS effects on follicular development and hormonal secretions during basal folliculogenesis.	BPS	*In vitro*	Isolated pre-antral follicles treated with 0.1 or 10 µM for 15 days	Ewes (cow), peri-pubertal	Seven independent experiments with eight follicles per treatment group, n > 150 animals total
Mechanisms underlying disruption of oocyte spindle stability by bisphenol compounds	Yang *et al.* (2020)	To test the acute impact of bisphenols on assembled MII spindle organization and stability.	BPA and BPF	*In vitro*	MII oocytes treated with 5, 25, or 50 µg/ml for 4 h	B6D2F1 mice, 20–21 days old	≥3 independent replicates per experiment, 30–60 oocytes per treatment group
Exploration of the damage and mechanisms of BPS exposure on the uterus and ovary of adult female mice	Yue *et al*. (2023a)	To explore the disruptive effects of BPS on the uterus and ovary of adult female mice.	BPS	*In vivo*	Delivered orally in corn oil at 300 µg/kg bw/day for 28 days	CD-1/ICR mice, collected at 3 months old	Nine animals per treatment group in total (three per group for ovarian histology)
Identification of risk for ovarian disease enhanced by BPB or BPAF exposure	Yue *et al.* (2023b)	To assess whether BPB or BPAF exposure has effects on normal ovarian function, differentially expressed genes, and the risk of ovarian diseases.	BPB and BPAF	*In vivo*	Delivered orally in corn oil at 300 µg/kg bw/day for 14 or 28 days	CD-1 (ICR) mice, collected at ∼3 months old	4–5 animals per treatment group
Bisphenol S negatively affects the meiotic maturation of pig oocytes	Žalmanová *et al*. (2017)	To explore the effects of BPS on the *in vitro* maturation of porcine oocytes.	BPS	*In vitro*	COCs treated with 30 pM, 3 nM, 300 nM, or 30 µM for 24, 48, or 72 h	Gilts (pigs), pre-pubertal	3–4 independent experiments, 82–120 oocytes per treatment group
The Bisphenol S contamination level observed in human follicular fluid affects the development of porcine oocytes	Žalmanová *et al*. (2023)	To assess the effect of BPS on *in vitro* oocyte maturation, fertilization, and embryo development using concentrations similar to those detected in ART clinic patients.	BPS	*In vitro*	COCs treated with 300 pM, 30 nM, or 3 µM for 48 h	Gilts (pig), non-cycling	3–8 independent experiments, 23–100 oocytes per treatment group
Bisphenol B exposure disrupts mouse oocyte meiotic maturation *in vitro* through affecting spindle assembly and chromosome alignment	Zhang *et al.* (2020a)	To evaluate the effects of BPB on mouse oocyte maturation and its related mechanisms *in vitro*.	BPB	*In vitro*	GV oocytes treated with 50, 100, 150, or 200 µM for 8 or 14 h	Kunming mice, 3–4 weeks old	Three independent experiments, 88–117 oocytes per treatment group
Maternal Bisphenol S exposure affects the reproductive capacity of F1 and F2 offspring in mice	Zhang *et al.* (2020b)	To assess how maternal BPS exposure affects the fertility parameters of F1 and F2 female offspring.	BPS	*In vivo* (*in utero*)	Delivered orally in saline to pregnant mice at 2, 10, 50, 100, or 200 mg/kg bw/day from 12.5–15.5 dpc	ICR mice, F1 collected at 15.5 dpc, PND3, PND21, or 5 weeks old. F2 collected at PND3 or PND21	Number of animals per treatment group not specified, ≥3 replicates per experiment , 174 animals utilised in total

**STUDIES THAT ASSESSED BPA**							

Bisphenol A alters oocyte maturation by prematurely closing gap junctions in the cumulus cell-oocyte complex	Acuña-Hernández *et al.* (2018)	To investigate whether BPA effects on oocyte meiotic division were correlated with reduced transfer in gap junction intercellular communication.	BPA	*In vitro*	COCs treated with 0.22, 2.2, 22, 220, or 2200 nM for 2 or 16 h	C57BL/6 mice, 4 weeks old	≥3 independent experiments, 15–57 COCs per treatment group
Neonatal Bisphenol-A exposure alters rat reproductive development and ovarian morphology without impairing activation of gonadotropin-releasing hormone neurons	Adewale *et al.* (2009)	To determine whether neonatal exposure to BPA or PTT (estrogen receptor agonist) induces similar malformations.	BPA	*In vivo*	Delivered subcutaneously in sesame oil at 50 µg/kg or 50 mg/kg bw/day for 4 days from PND0 to PND3	Long Evans rats, ovaries collected at ∼5 months old	10–12 animals per treatment group
Effect of Bisphenol A on alterations of ICAM-1 and HLA-G genes expression and DNA methylation profiles in cumulus cells of infertile women with poor response to ovarian stimulation	Aftabsavad *et al.* (2021)	To assess the relationship of follicular fluid BPA concentrations with gene expression, protein level and methylation status of ICAM-1 and HLA-G in the cumulus cells of infertile patients.	BPA	Human observational	Mean + SD: 4.73 + 2.23 ng/ml (without healthy lifestyle habit), 1.56 + 1.33 ng/ml (with healthy lifestyle habit)	Women participating in ICSI program with poor ovarian response, <35 years old, Iran	Eighty participants, follicular fluid collected
The effects of *in utero* Bisphenol A exposure on the ovaries in multiple generations of mice	Berger *et al.* (2016)	To examine whether BPA alters expression of insulin-like growth factor family, hormone receptors, and steroidogenesis-related genes.	BPA	*In vivo* (*in utero*)	Delivered orally to pregnant F0 females in corn oil at 0.5, 20, or 50 µg/kg bw/day for 10 days from gestational day 11 to birth	Inbred FVB mice, collected at PND4 or PND21	1–6 animals per treatment group
Bisphenol A exposure reduces the estradiol response to gonadotropin stimulation during *in vitro* fertilization	Bloom *et al.* (2011)	To assess associations between serum BPA, peak estradiol concentrations, and the number of oocytes retrieved during IVF.	BPA	Human observational	Median (Q1, Q3): 2.53 (0.52, 6.31) ng/ml	Women undergoing first IVF cycle, 28–44 years old, USA	44 participants, blood collected
Differential follicle counts as a screen for chemically induced ovarian toxicity in mice: Results from continuous breeding bioassays	Bolon *et al.* (1997)	To compare reproductive performance and differential follicle counts as endpoints for ovarian toxicity.	BPA	*In vivo* (*in utero*)	Dose unclear, delivered during a 7 day pre-mating period, and again to mothers over 93 days spanning the birth of multiple litters. Offspring are then dosed until 74 (+ or - 10) days of age, and then paired and bred.	CD-1 mice, collected at 50–240 days old	20–40 animals per sex per treatment group
Human meiotic progression and recombination are affected by Bisphenol A exposure during *in vitro* human oocyte development	Brieño-Enríquez *et al.* (2011)	To evaluate the effects of BPA on meiotic prophase in human fetal oocytes from cultured ovaries.	BPA	*In vitro*	Fetal ovaries treated with 1, 5, 10, 20, or 30 µM for 7, 14, or 21 days	Human fetuses, 18–22 weeks old	Six fetuses (12 ovaries and 21 510 oocytes total)
Gene expression is altered after Bisphenol A exposure in human fetal oocytes *in vitro*	Brieño-Enríquez *et al.* (2012)	To characterize the gene expression of human fetal oocytes in culture and evaluate the effect of BPA in cultured human oocytes.	BPA	*In vitro*	Fetal ovaries treated with 30 µM for 7, 14, or 21 days	Human fetuses, 18–22 weeks old	6 fetuses (12 ovaries)
A protective role of cumulus cells after short-term exposure of rat cumulus cell-oocyte complexes to lifestyle or environmental contaminants	Campen *et al.* (2017)	To investigate the effects of five lifestyle and environmental factors on gap junction function, gene expression within key regulatory pathways, and protein levels of gap junction protein connexin 43 in rat COCs.	BPA	*In vitro*	COCs treated with 20 ng/ml for 1–25 h	Sprague Dawley rats, 21–25 days old	Three experimental replicates, 2–7 rats per replicate, 10–12 COCs per treatment group
Bisphenol-A induces cell cycle delay and alters centrosome and spindle microtubular organization in oocytes during meiosis	Can *et al*. (2005)	To test the potential inhibitory effects of BPA on meiotic cell cycle progression, centrosomes and spindle integrity in mouse cumulus–oocyte complexes (COCs).	BPA	*In vitro*	COCs treated with 10 or 30 µM for 8 h (between GV and MI), or 10 h (between MI and MII) and one group with 10 h recovery	Balb/c mice, 19–21 days old	Experimental replicates not specified, ∼200–300 COCs per treatment group in total
Bisphenol A exposure modifies methylation of imprinted genes in mouse oocytes via the estrogen receptor signalling pathway	Chao *et al.* (2012)	To test the potential effects of BPA on methylation of imprinted genes during oocyte growth and meiotic maturation in CD-1 mice.	BPA	*In vivo*	Delivered hypodermically in saline + DMSO at 20 or 40 µg/kg bw/day from PND7 to PND 14 or every 5 days from PND5 to PND 20	CD-1 mice, collected on PND15 or PND21	Number of animals per treatment group not specified, 183 mice in total, ≥3 independent experiments
Soy intake modifies the relation between urinary bisphenol a concentrations and pregnancy outcomes among women undergoing assisted reproduction	Chavarro *et al.* (2016)	To examine whether soy intake modifies the association between BPA and fertility in women undergoing assisted reproduction.	BPA	Human observational	Median (Q1, Q3): 1.3 (0.9, 1.9) µg/l (SG-adjusted)	Women who completed at least 1 IVF cycle (EARTH), 18–45 years old, USA	239 participants, up to two spot urine samples per IVF cycle
Urinary Bisphenol A concentrations and early reproductive health outcomes among women undergoing IVF	Ehrlich *et al.* (2012)	To determine if urinary BPA concentrations were associated with ovarian response and early reproductive outcomes.	BPA	Human observational	Median (Q1, Q3): 2.32 (1.60, 3.76) µg/l (SG-adjusted)	Women undergoing oocyte retrieval (EARTH), 21–44 years old, USA	174 participants, up to 2 spot urine samples per IVF cycle
Exposure of mouse oocytes to Bisphenol A causes meiotic arrest but not aneuploidy	Eichenlaub-Ritter *et al.* (2008)	To evaluate the effect of BPA in an *in vitro* and *in vivo* setting on oocyte meiotic progression and chromosomal constitution.	BPA	*In vitro* and *in vivo*	***In vitro*:** GV oocytes treated with 50, 100, 200, 400, 800 ng/ml, or 10 µg/ml for 16 h ***In vivo*:** Delivered orally in corn oil to 22 day old F1 females at 20, 40, or 100 ng/g bw/day for 7 days	MF1 outbred mice (2–4 month old) and C57Bl×CBA/Ca F1 hybrid mice (collected at 28 days old)	***In vitro*:** ≥3 experimental replicates, 4–12 mice (∼150–450 oocytes) per treatment group in total ***In vivo*:** 8–20 animals per treatment group
Neonatal exposure to Bisphenol A and reproductive and endocrine alterations resembling the polycystic ovarian syndrome in adult rats	Fernández *et al.* (2010)	To investigate the effects of neonatal exposure to BPA on the reproductive axis in adult female Sprague Dawley rats.	BPA	*In vivo*	Delivered subcutaneously in castor oil at 50 µg/50 µl (6.2–2.5 mg/kg), 500 µg/50 µl (62.5–25.0 mg/kg), or 5 µg/50 µl (0.62–0.25 mg/kg) from PND1 to PND 10	Sprague Dawley rats, collected at 4–5 months old	5–7 animals per treatment group
Bisphenol A exposure during oocyte maturation *in vitro* results in spindle abnormalities and chromosome misalignment in *Bos taurus*	Ferris *et al.* (2015)	To evaluate the effects of BPA during bovine *in vitro* oocyte maturation on meiotic progression, spindle formation, and chromosome alignment in MII oocytes.	BPA	*In vitro*	COCs treated with 15 ng/ml (65 nM) or 30 ng/ml (130 nM) for 24 h	*Bos taurus* (cow), age not specified	Experimental replicates/animal numbers not specified, ∼20–100 COCs per treatment group
BPA exposure during *in vitro* oocyte maturation results in dose-dependent alterations to embryo development rates, apoptosis rate, sex ratio and gene expression	Ferris *et al.* (2016)	To assess impacts of BPA exposure on COCs maturing *in vitro* and subsequent embryonic developmental rates, transcript composition, sex ratio, cell number, and rates of apoptosis.	BPA	*In vitro*	COCs treated with 15 ng/ml (65 nM) or 30 ng/ml (130 nM) for 24 h	Bovine, age not specified	Experimental replicates/animal numbers not specified, ∼250–320 zygotes per treatment group
Serum unconjugated Bisphenol A concentrations in women may adversely influence oocyte quality during *in vitro* fertilization	Fujimoto *et al.* (2011)	To measure serum BPA levels in women undergoing IVF and correlate these with oocyte maturation and fertilization outcomes.	BPA	Human observational	Median: 2.53 ng/ml	Women undergoing first IVF cycle, 28–44 years old, USA	44 participants, blood collected
Bisphenol A-induced ovotoxicity involves DNA damage induction to which the ovary mounts a protective response indicated by increased expression of proteins involved in DNA repair and xenobiotic biotransformation	Ganesan and Keating (2016)	To determine ovarian effects of BPA exposure, including DNA repair and xenobiotic biotransformation in culture of post natal day 4 rat ovaries.	BPA	*In vitro*	Ovaries treated with 440 µM for 2–8 days	F344 rats, PND4	n = 5 (5 ovaries per treatment)
Mixtures of urinary concentrations of phenols and phthalate biomarkers in relation to the ovarian reserve among women attending a fertility clinic	Génard-Walton *et al.* (2023)	To evaluate the joint effects of urinary phenol and phthalate metabolite concentrations, as a mixture, in relation to the ovarian reserve among women consulting in a fertility clinic.	BPA	Human observational	Median (Q1, Q3): 1.0 (0.5, 1.9) µg/l (SG-adjusted)	Infertile women, 18–45, USA (Earth study)	271 participants, 1–14 urine samples collected per participant over 1 or more cycles
NTS, NTSR1 and ERs in the pituitary-gonadal axis of cycling and postnatal female rats after BPA treatment	González-Gómez *et al.* (2023)	To understand the effects of BPA treatment during gestation and lactation on the hypophysis-gonadal axis of female rat offspring.	BPA	*In vivo* (*in utero*, and through breast milk)	Delivered subcutaneously to pregnant mothers in sesame oil at 0.5 or 2 mg/kg bw/day during gestation (21 days) and 21 days of lactation	Sprague Dawley rats, collected from 1 to 6 weeks old	Five pregnant mothers per treatment group, with five offspring collected weekly from week 1 to 6
Effect of Bisphenol A level in follicular fluid on ICSI outcome	Herez *et al.* (2022)	To assess the impact of Bisphenol A in follicular fluid on ICSI outcome.	BPA	Human observational	Mean + SEM: 45.44 + 1.73 ng/ml (pregnant), 68.23 + 6.77 ng/ml (non-pregnant)	Infertile women, 20–42 years old, Iraq	Sixty participants, follicular fluid collected during oocyte retrieval
Bisphenol A initiates excessive premature activation of primordial follicles in mouse ovaries via the PTEN signalling pathway	Hu *et al.* (2018)	To observe the effects of BPA on follicular activation and development, depletion of the primordial follicle pool, and the modulation of the PTEN signalling pathway in the premature activation.	BPA	*In vivo*	Delivered orally to 6 week old mice in corn oil at 1 μg, 10 μg, 100 μg, 1 mg, and 10 mg/kg bw/day for 14–42 days	CD-1 mice, collected at ∼8–12 weeks old	13–30 animals per treatment group
Combinational exposure to Bisphenol A and a high-fat diet causes trans-generational malfunction of the female reproductive system in mice	Huang *et al.* (2022)	To evaluate the simultaneous exposure of BPA and high-fat diet on female mouse reproduction and trans-generational effects.	BPA	*In vivo* (pre-gestation, *in utero*, and breast milk)	Delivered orally to F0 generation females in PBS at 500 µg/kg bw/day for 10 weeks pre-gestation, gestation (3 weeks), and lactation (3 weeks)	ICR mice, F1 and F2 animals collected at 24 weeks old	4–5 animals per treatment group in each generation, each F1 and F2 mouse from a different litter
Bisphenol A exposure causes meiotic aneuploidy in the female mouse	Hunt *et al.* (2003)	To understand the cause of increased meiotic chromosome abnormalities in mice exposed to damaged caging and recreate these abnormalities with a BPA treatment.	BPA	*In vivo*	Delivered orally to 20–22 day old mice in corn oil at 20, 40, or 100 ng/g bw/day for 6–8 days OR 20 ng/g bw/day for 3, 5, or 7 days prior to oocyte collection	Mice (strain not specified), collected at 28 days old	Number of animals or replicates per treatment group not specified
Bisphenol A alters early oogenesis and follicle formation in the fetal ovary of the rhesus monkey	Hunt *et al.* (2012)	To determine whether BPA induces disturbances in meiotic progression of oocytes and follicle formation in the developing primate ovary.	BPA	*In vivo* (*in utero*)	Delivered orally to pregnant macaques in fruit at 400 µg/kg bw/day or continuously from an implant to produce maternal serum levels of 2.2–3.3 ng/ml from gestational day 50–100 or 100 term	Rhesus macaques, fetuses collected at 50 days of gestation or at term	2–6 animals per treatment group
Deleterious effects of endocrine disruptors are corrected in the mammalian germline by epigenome reprogramming	Iqbal *et al.* (2015)	To evaluate the effects of endocrine disruptors on global epigenetic reprogramming and imprint resetting in the male germline after *in utero* exposure.	BPA	*In vivo* (*in utero*)	Delivered orally in corn oil to pregnant F0 females at 0.2 mg/kg bw/day alongside other endocrine disruptors starting at 8.5 or 12.5 dpc for 5 days	Transgenic TgOG2, inbred FVB, 129S1, and JF1 mice, collected at 13.5–17.5 dpc	Animal numbers per treatment group not provided, 2–3 replicates for all experiments
Effects of estrogenic compounds on neonatal oocyte development	Karavan and Pepling (2012)	To determine if exposure to synthetic estrogens, diethylstilbestrol, ethinyl estradiol and Bisphenol A affected perinatal oocyte development.	BPA	*In vivo*	Delivered subcutaneously in peanut oil at 5 or 50 mg/kg bw/day for 4 days from PND1 to PND 4	CD1 outbred mice, collected at PND5	6–12 animals per treatment group
Body fluid concentrations of Bisphenol A and their association with *in vitro* fertilization outcomes	Kim *et al.* (2021)	To examine couples who underwent IVF procedures, and assess the association between BPA concentrations in various body fluids and IVF outcomes.	BPA	Human observational	Median (Q1, Q3): urine: 0.66 (0.27, 5.13) ng/ml (SG- adjusted), plasma: 0.118 (0.036, 0.229) ng/ml, follicular fluid: 0.063 (0.021, 0.147) ng/mL	Women seeking infertility treatment, 24–48 years old, Korea	146 participants, follicular fluid, urine, and plasma collected on day of follicle aspiration
Continuous exposure to Bisphenol A during *in vitro* follicular development induces meiotic abnormalities	Lenie *et al.* (2008)	To analyze the effects of chronic BPA exposure (3 nM to 30 μM) on follicle-enclosed growth and maturation of mouse oocytes *in vitro*.	BPA	*In vitro*	Early pre-antral follicles treated with 3 nM, 30 nM, 300 nM, 3 μM, or 30 μM for 12 days	F1 hybrid mice (C57BL/6j × CBA/Ca), age not specified	Eight experimental replicates using 32 animals, ∼80–100 oocytes per treatment group in total
Prepubertal Bisphenol A exposure interferes with ovarian follicle development and its relevant gene expression	Li *et al.* (2014)	To assess the effects of BPA on ovarian structure and function, measuring expression levels of follicle development-related genes.	BPA	*In vivo*	Delivered intraperitoneally in olive oil at 10, 40, or 160 mg/kg bw/day for 7 days (Day 28–35)	Wistar rats, collected at 35 days old	12 animals per treatment group
Astaxanthin improves the development of the follicles and oocytes through alleviating oxidative stress induced by BPA in cultured follicles	Li *et al.* (2022)	To investigate whether astaxanthin could alleviate BPA-induced oxidative stress damage of follicles and improve their development.	BPA	*In vitro*	Isolated pre-antral follicles treated with 25 µmol/l for 11 days	Kunming mice, 14 days old	Experimental replicates of treatments not provided, used 90 animals, and 180 follicles per treatment group in total
In utero Bisphenol A exposure disturbs germ cell cyst breakdown through the PI3k/Akt signaling pathway and BDNF expression	Li *et al.* (2023a)	To examine the effect and mechanism of exposure to low levels of BPA in utero during the critical ovarian developmental window.	BPA	*In vivo (in utero)*	Delivered orally to pregnant mothers in corn oil at 2 or 20 µg/kg bw/day for 10 days from gestational day 11 until birth	CD-1/ICR mice, collected at PND4 or PND22	Six pregnant mothers and 10 pups per treatment group
BPA interferes with granulosa cell development and oocyte meiosis in mouse preantral follicles	Li *et al.* (2023b)	To investigate the effects of BPA on granulosa cell development and meiosis of oocytes using *in vitro* culture system of mouse pre-antral follicles.	BPA	*In vitro*	Isolated pre-antral follicles treated with 10 µg/ml for 11 days	Kunming mice, 14 days old	Experimental replicates of treatments not provided, used 90 animals, and 40–250 COCs/oocytes per treatment group in total
Influence of N-acetyl-L-cysteine against Bisphenol A on the maturation of mouse oocytes and embryo development: *in vitro* study	Li and Zhao (2019)	To investigate the effect of BPA exposure with NAC during oocyte IVM on oocyte maturation, embryo developmental potential and oxidative stress markers.	BPA	*In vitro*	COCs treated with 20, 50, or 100 µg/ml for 14 h	Kunming mice, 6–8 weeks old	≥3 experimental replicates, 30–40 oocytes per treatment group per replicate
Bisphenol A promotes autophagy in ovarian granulosa cells by inducing AMPK/mTOR/ULK1 signalling pathway	Lin *et al.* (2021)	To determine the adverse effects of BPA on normogonadotropic patients and the mechanism of its toxicity on human granulosa cells.	BPA	Human observational and *in vivo*	Human observational: median (Q1, Q3): 0.66 (0.29, 1.14) µg/l (Cr-adjusted) *In vivo*: Delivered orally in peanut oil to 6–8 weeks old mice at 1, 10, or 100 µg/kg bw/day for 2 weeks	Human observational: normogonadotropic infertile patients undergoing IVF/ICSI, 20–40 years old, China *In vivo*: Kunming mice, collected at ∼8–10 weeks old	Human observational: 106 participants, blood samples and two urine samples collected per cycle *In vivo*: six animals per treatment group
Bisphenol A deteriorates egg quality through HDAC7 suppression	Liu *et al.* (2017)	To investigate the role of HDAC7 in eggs with BPA treatment, and determine the epigenetic effect of BPA for egg maturation and vitality.	BPA	*In vivo*	Delivered orally in corn oil at 50 µg/kg bw/day, treatment time not specified	C57BL/6 outbred mice, 4–6 weeks old	Animal numbers or replicates per treatment group not provided
BPA disrupts meiosis I in oogonia by acting on pathways including cell cycle regulation, meiosis initiation and spindle assembly	Loup *et al.* (2022)	To investigate the effects of BPA on prophase I meiosis in the fetal sheep ovary.	BPA	*In vitro*	Fetal ovary explants treated with 3 µM or 30 µM for 20 days	Pré-Alpes ewe (sheep) fetuses, 50, 60, and 70 dpc	Experimental replicates of treatments not provided, three ovary pieces from different fetuses per treatment group
Bisphenol-A and human oocyte maturation *in vitro*	Machtinger *et al.* (2013)	To determine whether BPA exposure perturbs meiotic maturation, spindle organization and chromosome alignment in human oocytes *in vitro*.	BPA	*In vitro*	GV oocytes treated with 20 ng/ml, 200 ng/ml, or 20 µg/ml BPA for 30 h	Human patients, 23.9–43.8 years old	121 patients, ≥2 oocytes from each patient, 5–100 oocytes per treatment group
Stereological study on the effect of vitamin C in preventing the adverse effects of Bisphenol A on rat ovary	Mehranjani and Mansoori (2016)	To investigate the effect of vitamin C on the ovary tissue in rats treated with BPA using stereological methods.	BPA	*In vivo*	Delivered orally in corn oil at 60 µg/kg bw/day for 20 days	Wistar rats, adult	Six animals per treatment group
Bisphenol A correlates with fewer retrieved oocytes in women with tubal factor infertility	Mina *et al.* (2022)	To evaluate associations among serum, urinary, and follicular fluid BPA concentrations and the number of retrieved and fertilized oocytes and pregnancy rates in women with PCOS or tubal factor infertility.	BPA	Human observational	Median (Q1, Q3) (PCOS, tubal factor infertility): urine: 0.80 (0.48, 1.27) µg/g, 0.73 (0.40, 1.14) µg/g (Cr- adjusted), serum: 0.58 (0.16, 0.94) ng/ml, 0.78 (0.39, 1.21) ng/ml, follicular fluid: 0.50 (0.14, 1.15) ng/ml, 1.13 (0.40, 2.05) ng/ml	Caucasian women with PCOS or tubal factor infertility, mean ages 35–38, Greece	93 participants, follicular fluid, blood, and urine collected at time of oocyte retrieval
Urinary Bisphenol A concentrations and association with *in vitro* fertilization outcomes among women from a fertility clinic.	Mínguez-Alarcón *et al.* (2015)	To reevaluate, in a larger number of women from the same cohort as Mok-Lin *et al.* (2010) and Ehrlich *et al.* (2012), the associations of urinary BPA concentrations with early IVF outcomes.	BPA	Human observational	Median (Q1, Q3): 1.38 (0.97, 2.24) µg/l (SG-adjusted)	Women undergoing IVF, 32.5–39 years old (Q1–Q3), USA (EARTH study)	256 participants, up to 2 urine samples collected per cycle
Dietary folate intake and modification of the association of urinary Bisphenol A concentrations with *in vitro* fertilization outcomes among women from a fertility clinic	Mínguez-Alarcón *et al.* (2016)	To explore whether intake of folate and other methyl donors modified the association between urinary BPA concentrations and IVF outcomes.	BPA	Human observational	Median (Q1, Q3): 1.3 (0.9, 1.9) µg/l (SG-adjusted)	Women undergoing IVF, 32–38 years old (Q1–Q3), USA (EARTH study)	178 participants, up to 2 urine samples collected per cycle
Urinary concentrations of Bisphenol A, parabens and phthalate metabolite mixtures in relation to reproductive success among women undergoing *in vitro* fertilization	Mínguez-Alarcón *et al.* (2019)	To investigate whether urinary concentrations reflecting mixtures of BPA, parabens and phthalates were associated with reproductive outcomes among women who underwent IVF.	BPA	Human observational	Median (Q1, Q3): 1.10 (0.71, 1.75) µg/l (SG-adjusted)	Women undergoing IVF, 32–39 years old (Q1–Q3), USA (EARTH study)	420 participants, up to 2 urine samples collected per cycle
Effects of selected endocrine disruptors on meiotic maturation, cumulus expansion, synthesis of hyaluronan and progesterone by porcine oocyte-cumulus complexes	Mlynarcíková *et al*. (2009)	To examine the effect of phenols (BPA and CMP) and phthalates (DEHP and BBP) on oocyte maturation and cumulus expansion in cultured porcine COCs, and production of factors by cumulus cells.	BPA	*In vitro*	COCs treated with 0.0001, 0.01, 1, or 100 μM for up to 44 h	Porcine, age not specified	Four independent experiments, ∼40–200 COCs per treatment group in total
Estrogen and Bisphenol A disrupt spontaneous [Ca(2+)]_i_ oscillations in mouse oocytes	Mohri and Yoshida (2005)	To assess the effects of estrogen or endocrine disrupters (EDs) on the dynamic changes in intracellular Ca2+ concentration of mouse immature oocytes.	BPA	*In vitro*	GV oocytes treated with 1 nM, 10 nM, 100 nM, 10 μM, or 100 µM for 60 min	CD-1/ICR mice, 8–12 weeks old	Experimental or biological replicates not provided, 15–40 oocytes per treatment group
Urinary Bisphenol A concentrations and ovarian response among women undergoing IVF	Mok-Lin *et al.* (2010)	To investigate the association of pre- and peri-conception urinary BPA concentrations with oocyte and oestradiol production among women undergoing IVF.	BPA	Human observational	Median (Q1, Q3): 2.28 (1.46, 4.00) µg/l (SG-adjusted)	Women undergoing IVF, 21–44 years old, USA (EARTH)	84 women, 2 urine samples collected per cycle
Exposure to Bisphenol A in young adult mice does not alter ovulation but does alter the fertilization ability of oocytes	Moore-Ambriz *et al.* (2015)	To evaluate whether BPA alters ovulation, oocyte fertilization rate, and early zygote development.	BPA	*In vivo*	Delivered orally in corn oil to 28–32-day old mice at 50 µg/kg bw/day from the day of first estrus for three estrus cycles	C57BL/6J mice, collected after third estrus cycle	Six animals per treatment group
Bisphenol A effects on the growing mouse oocyte are influenced by diet	Muhlhauser *et al.* (2009)	To evaluate the effect of diet containing high or low phytoestrogen content on BPA and its effects on pre-ovulatory oocyte health outcomes.	BPA	*In vivo*	Delivered orally in corn oil at 20, 40, 100, 200, or 500 µg/kg bw/day at 21 days old for 7 days	C57BL/6J mice, collected at 28 days old	Animal numbers or replicates per treatment group not provided
Environmentally induced epigenetic transgenerational inheritance of ovarian disease	Nilsson *et al.* (2012)	To determine if exposure to environmental toxicants has the capacity to promote epigenetic transgenerational inheritance of a disease phenotype.	BPA	*In vivo* (*in utero*)	Delivered intraperitoneally in sesame oil only to pregnant F0 rats at 50 mg/kg bw/day within a mixture of other plastics (DBP and DEHP) on days E8–E14 gestation. A half dose group was also created due to small litter sizes.	Hsd: Sprague Dawley outbred rats, F1, F2, and F3 generation rats collected at 1 year old	Nine animals per treatment group
The effects of prenatal and lactational Bisphenol A and/or di(2-ethylhexyl) phthalate exposure on female reproductive system	Ozkemahli *et al*. (2022)	To evaluate the effects of single and combined prenatal and lactational exposure to BPA and/or DEHP on the adult rat female reproductive system.	BPA	*In vivo* (*in utero*, and through breast milk)	Delivered orally to pregnant mothers in corn oil at 50 mg/kg bw/day for 16 days during gestation (days 6–21) and 21 days of lactation	Sprague Dawley rats, collected at 10 weeks old	Three pregnant mothers and 5–6 pups in each treatment group
Evaluation of aneugenic effects of Bisphenol A in somatic and germ cells of the mouse	Pacchierotti *et al.* (2008)	To analyze the frequency of micronuclei in bone marrow erythrocytes, and hyperploidy in oocytes, sperm and zygotes of mice exposed to acute, sub-chronic or chronic low doses of BPA.	BPA	*In vivo*	Delivered orally in corn oil at 0.2 or 20 mg/kg bw in a single dose (acute), 0.04 mg/kg for 7 days (sub-acute), or 0.5 mg/l for 7 weeks in drinking water (chronic)	C57Bl/6 mice, collected at 4–11 weeks old	Animal numbers or replicates per treatment group not provided
Bisphenol A exposure disrupts organelle distribution and functions during mouse oocyte maturation	Pan *et al.* (2021)	To investigate whether BPA can be toxic to organelles within oocytes *in vitro*.	BPA	*In vitro*	GV oocytes treated with 50, 100, or 200 µM for 3 or 12 h	Mice, strain and age not specified	≥3 biological replicates, ∼100–250 oocytes per treatment group
Melatonin improves oocyte maturation and mitochondrial functions by reducing Bisphenol A-derived superoxide in porcine oocytes *in vitro*	Park *et al.* (2018)	To confirm the protective role of melatonin in BPA exposure during meiotic maturation and cumulus cell expansion in maturing porcine COCs.	BPA	*In vitro*	COCs treated with 50, 75, or 100 µM for 22–44 h	Yorkshire/Landrace × Duroc pigs, 6 months old	Three experimental replicates, ∼140–160 oocytes or ∼340–380 COCs per treatment group in total
Level of Bisphenol A in follicular fluid and serum and oocyte morphology in patients undergoing IVF treatment	Poormoosavi *et al.* (2019)	To assess the correlation between the levels of BPA in the serum and follicular fluid using oocyte morphology.	BPA	Human observational	Median (Q1, Q3) and/or mean + SD not provided	Women receiving ART treatment for infertility, 20–45 years old, Iran	90 participants, serum and follicular fluid collected on day of oocyte retrieval
Bisphenol A exposure modulates reproductive and endocrine system, mitochondrial function and cellular senescence in female adult rats: A hallmarks of polycystic ovarian syndrome phenotype	Prabhu *et al*. (2022)	To examine the molecular patho-mechanisms in a BPA-induced PCOS rat model.	BPA	*In vivo*	Delivered orally in corn oil at 0.001 or 0.1 mg/kg bw/day for 90 days	Sprague Dawley rats, collected at 9 months old	Six animals per treatment group
Urinary Bisphenol A concentrations and *in vitro* fertilization outcomes among women from a fertility clinic	Radwan *et al.* (2020)	To examine the association between urinary BPA concentration and *in vitro* reproductive outcomes among women from an infertility clinic.	BPA	Human observational	Median (Q1, Q3): 2.28 (1.46, 4.00) µg/l (SG-adjusted)	Women undergoing IVF, 24–44 years old, Poland	450 participants, ≥1 urine sample collected per cycle prior to egg retrieval
Neonatal exposure to Bisphenol A or diethylstilbestrol alters the ovarian follicular dynamics in the lamb	Rivera *et al.* (2011)	To test whether neonatal exposure to low doses of BPA or DES adversely affects the pre-pubertal lamb ovary.	BPA	*In vivo*	Delivered subcutaneously in corn oil at 50 µg/kg bw/day for 14 days from PND1 to PND 14	Corriedale × Hampshire down lambs, ovaries collected at PND30	6–10 animals per treatment group
Neonatal exposure to Bisphenol A reduces the pool of primordial follicles in the rat ovary	Rodríguez *et al*. (2010)	To investigate whether neonatal exposure to BPA is able to disrupt early follicle development in rats.	BPA	*In vivo*	Delivered subcutaneously in corn oil at 0.05 or 20 mg/kg every 48 h at PND1, 3, 5, and 7	Wistar rats, collected at PND8	8–10 animals each from a different litter per treatment group
Impairment of steroidogenesis and follicle development after Bisphenol A exposure during pregnancy and lactation in the ovaries of Mongolian gerbils aged females	Ruiz *et al.* (2023)	To describe the histopathological repercussions of BPA exposure in pregnancy and lactation in aged ovaries using a rodent model.	BPA	*In vivo*	Delivered orally to pregnant mothers in corn oil at 50 µg/kg bw/day for 39 days (from Day 8 of gestation until end of lactation)	Mongolian gerbils (mothers), collected at 18 months old	5 animals per treatment group
Ovarian dysfunctions in adult female rat offspring born to mothers perinatally exposed to low doses of Bisphenol A	Santamaría *et al.* (2016)	To investigate ovarian folliculogenesis and steroidogenesis in adult female rat offspring of mothers exposed orally to low doses of BPA during gestation and breastfeeding.	BPA	*In vivo* (*in utero*, and through breast milk)	Delivered orally in drinking water to pregnant rats at 0.5 or 50 µg/kg bw/day from gestational day 9 to weaning at PND21	Wistar-derived rats, collected at PND90	10–12 dams (mothers) and at least 10 F1 animals per treatment group
Urinary Bisphenol A concentration is correlated with poorer oocyte retrieval and embryo implantation outcomes in patients with tubal factor infertility undergoing *in vitro* fertilisation	Shen *et al.* (2020)	To investigate the effects of BPA on female reproduction and the associations between BPA exposure and the outcomes of IVF-ET (embryo transfer).	BPA	Human observational	Median (Q1, Q3): 0.72 (0.25, 2.02) µg/l (Cr-adjusted)	Women seeking IVF-ET (embryo transfer) treatments, 28–34 (Q1–Q3) years old, China	351 participants, 1 urine sample collected on day of oocyte retrieval
Endocrine disruptors *in utero* cause ovarian damages linked to endometriosis	Signorile *et al.* (2012)	To investigate the long-term effect of prenatal BPA exposure on murine ovary development and in the context of an endometriosis-like phenotype.	BPA	*In vivo* (*in utero* and breast milk)	Delivered subcutaneously to pregnant mothers in PBS at 100 or 1000 µg/kg bw/day during gestation (3 weeks) and 1 week post-natal (4 weeks total)	BALB-C mice, collected at 3 months old	6 pregnant mothers and 20 offspring per treatment group
The association of Bisphenol-A urinary concentrations with antral follicle counts and other measures of ovarian reserve in women undergoing infertility treatments	Souter *et al.* (2013)	To evaluate the association between urinary BPA concentrations and antral follicle counts among women undergoing fertility treatments.	BPA	Human observational	Median (Q1, Q3): 1.6 (0.9, 2.3) µg/l (SG-adjusted)	Women undergoing infertility treatments, 21.6–46.7 years old, USA (Earth study)	209 participants (154 for follicle counts), ≥1 urine sample collected per cycle prior to follicle counts
Bisphenol A exposure *in utero* disrupts early oogenesis in the mouse	Susiarjo *et al.* (2007)	To assess how meiosis is affected in oocytes from females exposed to low, environmentally relevant doses of BPA during a 1-week fetal exposure.	BPA	*In vivo* (*in utero*)	Delivered through an implant into pregnant females at 20 µg/kg bw/day at 11.5 days gestation for 1 week until endpoint	C57BL/6 mice, collected at 18.5 days gestation or 4–5 weeks old	6–16 animals per treatment group
Chronic exposure to a low concentration of Bisphenol A during follicle culture affects the epigenetic status of germinal vesicles and metaphase II oocytes	Trapphoff *et al.* (2013)	To determine whether exposure to low concentrations of BPA during follicle culture and oocyte growth alters gene methylation and histone posttranslational modification.	BPA	*In vitro*	Pre-antral follicles treated with 3 or 300 nM for 12 or 13 days	C57/Bl6J × CBA/Ca F1 mice, age not specified	5–10 biological replicates, 3–5 mice per replicate, and ∼75–300 follicles/oocytes per treatment group per replicate
The toxic effects and possible mechanisms of Bisphenol A on oocyte maturation of porcine *in vitro*	Wang *et al.* (2016)	To evaluate the influence of acute exposure to BPA and DEHP on porcine oocyte maturation, subcellular structure, epigenetic modification, oxidative stress, autophagy, and apoptosis.	BPA	*In vitro*	COCs treated with 200 or 250 μM for 44 or 60 h	Gilts (pigs), prepubertal	≥3 biological replicates, ∼100–350 COCs/oocytes per treatment group in total
Interfering effects of Bisphenol A on *in vitro* growth of preantral follicles and maturation of oocytes	Wang *et al.* (2018)	To study the interfering effects of BPA on the growth of pre-antral follicles, the proliferation of granulosa cells and the maturation of oocytes *in vitro*.	BPA	*In vitro*	Pre-antral follicles treated with 4.5 or 45 µM for 11 days	Kunming mice, 14 days old	3–10 replicates, 20–500 follicles/oocytes per treatment group in total
The effect of plastic bottled water consumption on outcomes of ICSI cycles undertaken for unexplained infertility	Yenigül *et al.* (2021)	To evaluate whether BPA levels in maternal urine, serum, and follicular fluid could affect embryo quality and ICSI cycle outcomes in women with unexplained infertility.	BPA	Human observational	Mean + SD (tap water, plastic bottled water): urine: 7.0 + 3.0 ng/ml, 8.1 + 3.9 ng/ml, serum: 10.2 + 7.7 ng/ml, 22.6 + 17.1 ng/ml, follicular fluid: 7.4 + 6.9 ng/ml, 14.4 + 10.0 ng/ml	Women undergoing ICSI with unexplained infertility, 23–33 years old, Turkey	82 participants, urine, serum, and follicular fluid collected on day of oocyte retrieval
Fetal exposure to Bisphenol A affects the primordial follicle formation by inhibiting the meiotic progression of oocytes	Zhang *et al*. (2012a)	To assess the effects of BPA on germ cell cyst breakdown and primordial follicle formation.	BPA	In vivo (in utero)	Delivered orally in DMSO to pregnant mice at 0.02, 0.04, or 0.08 mg/kg from 12.5 to 18.5 dpc with pups delivered at 19.5	CD-1 mice, collected at 15.5 dpc, 17.5 dpc, 19.5 dpc, PND3, PND5, and PND7	7–8 mice per treatment group
Bisphenol A exposure modifies DNA methylation of imprint genes in mouse fetal germ cells	Zhang *et al.* (2012b)	To assess the effects of BPA on DNA methylation of imprinting genes in fetal mouse germ cells.	BPA	*In vivo* (*in utero*)	Delivered orally in DMSO to pregnant mice at 40, 80, or 160 µg/kg bw/day from 0.5 to 12.5 dpc	CD-1 mice, collected at 12.5 dpc	Animal numbers per treatment group not provided, ≥3 independent replicates per experiment
Di-(2-ethylhexyl) phthalate and Bisphenol A exposure impairs mouse primordial follicle assembly *in vitro*	Zhang *et al.* (2014)	To examine the effects of BPA and DEHP exposure on primordial follicle formation.	BPA	*In vitro*	Ovaries treated with 10 or 100 μM for 3 days	CD-1 mice, newborn	≥3 experimental replicates, ovary numbers not specified
Melatonin protects oocyte quality from Bisphenol A-induced deterioration in the mouse	Zhang *et al.* (2017)	To evaluate oocyte quality of control, BPA-exposed, and ‘BPA + melatonin’-administered groups by investigating their oocyte meiotic maturation and fertilization ability.	BPA	*In vivo*	Delivered orally in corn oil at 100 µg/kg bw/day at 9 a.m. for 7 days prior to oocyte collection	ICR mice, 4–6 week old	Animal numbers per treatment group not provided, ≥3 independent experiments
Exposure to Bisphenol A at physiological concentrations observed in Chinese children promotes primordial follicle growth through the PI3K/Akt pathway in an ovarian culture system	Zhao *et al.* (2014)	To assess the effect of BPA on the primordial follicle pool by employing a neonatal ovarian culture system.	BPA	*In vitro*	Ovaries treated with 0.1, 1, or 10 µM for 5 or 10 days	C57BL/6 mice, PND4	Five biological replicates/ovaries per treatment group
Bisphenol A exposure inhibits germ cell nest breakdown by reducing apoptosis in cultured neonatal mouse ovaries	Zhou *et al.* (2015)	To assess whether BPA exposure inhibits germ cell nest breakdown by inhibiting oxidative stress and/or apoptotic pathways.	BPA	*In vitro*	Ovaries treated with 0.1, 1.0, 5.0, or 10 µg/ml for 1–8 days	CD-1 mice, PND0	3–6 independent experiments, ovary numbers not specified
Bisphenol A and ovarian reserve among infertile women with polycystic ovarian syndrome	Zhou *et al.* (2017)	To better understand possible effects of BPA exposure on ovarian reserve in women with polycystic ovary syndrome (PCOS).	BPA	Human observational	Median (Q1, Q3): 2.35 (1.47, 3.95) µg/l (Cr-adjusted)	Infertile women with PCOS, 25–32 (Q1–Q3) years old, China	268 participants, 1 urine sample collected per participant
Effects of Bisphenol A on ovarian follicular development and female germline stem cells	Zhu *et al*. (2018)	To investigate the effects and potential mechanism of BPA on mouse ovarian follicular development and female germline stem cells.	BPA	*In vivo*	Delivered intraperitoneally in DMSO at 12.5, 25, or 50 mg/kg bw/day for 10 days	CD-1 mice, 6 weeks old	3 animals per treatment group

**Abbreviations:** BADGE, Bisphenol A diglycidyl ether; BHPF, Fluorene-9-bisphenol; BPA, Bisphenol A; BPAF, Bisphenol AF; BPB, Bisphenol B; BPF, Bisphenol F; BPS, Bisphenol S; bw, body weight; COC, cumulus-oocyte complex; Cr, creatinine; DES, diethylstilbestrol; DMSO, dimethylsulfoxide; DPC, days post coitum; EARTH study, The Environment and Reproductive Health study; GV, germinal vesicle; ICSI, intracytoplasmic sperm injection; IVF, *in vitro* fertilisation; IVM, *in vitro* maturation; LOAEL, lowest observed adverse effect level; MI, metaphase I; MII, metaphase II; NOAEL, no observed adverse effect level; PCOS, polycystic ovarian syndrome; PND, postnatal day; SG, specific gravity; TDI, tolerable daily intake.

**Figure 3.**
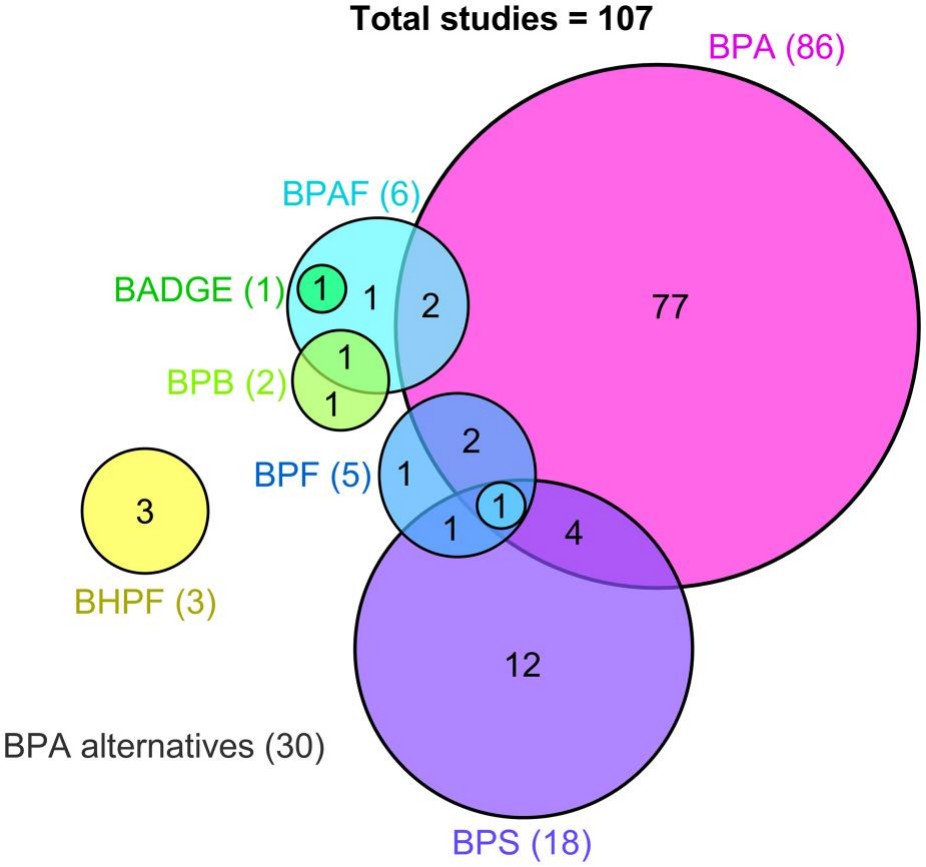
**A modified Venn diagram representing 107 studies for final inclusion in this review after the second search and the bisphenols they assessed.** Each coloured circle represents the number of studies in this review (not to scale) assessing a bisphenol, or multiple bisphenols (overlapping areas): Bisphenol A (BPA, 86), Bisphenol S (BPS, 18), Bisphenol F (BPF, 5), Bisphenol AF (BPAF, 6), Bisphenol B (BPB, 2), and Bisphenol A diglycidyl ether (BADGE, 1). The total number of studies assessing each bisphenol are provided in brackets.

Out of all 107 studies, there were 86 (80.4%) that investigated BPA and 30 (28.0%) that investigated BPA alternatives including: BPS (18), BPAF (6), BPF (5), BHPF (3), and BPB (2) (Fig. 3). Nine of these studies examined BPA alongside one or more BPA alternatives, and two studies examined two BPA alternatives in combination (Nevoral *et al.*, 2021). There was also one study that assessed BPAF alongside Bisphenol A diglycidyl ether (BADGE) (Fig. 3), the resulting basic monomer of the process used to create BPA-based epoxy resins (Abdallah *et al.*, 2023; Yue *et al.*, 2023b). These studies were published between 1997 to 2023 for BPA, and 2016 to 2023 (year the most recent search was conducted) for the BPA alternatives.

**Figure 4.**
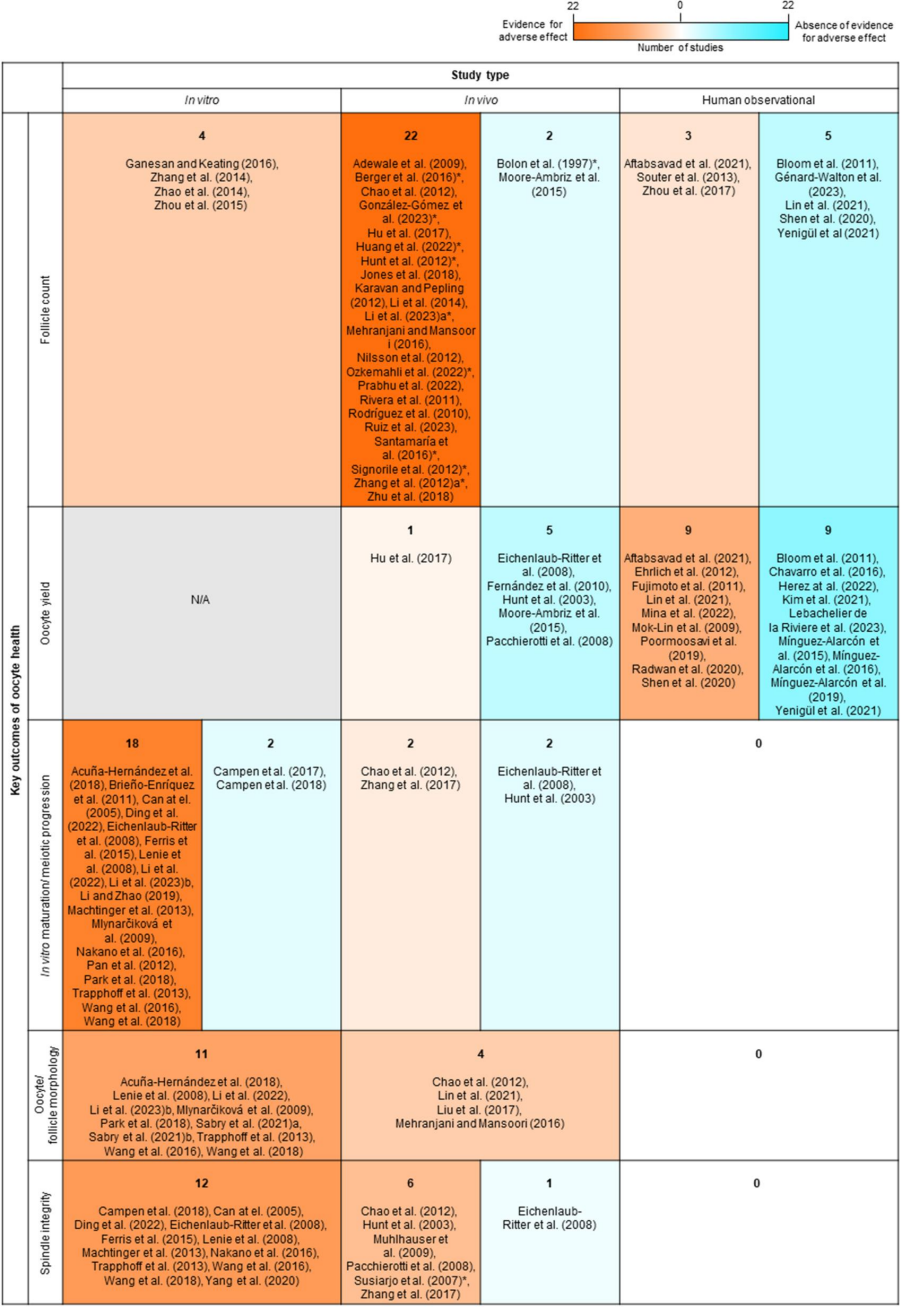
**Studies assessing the effect of BPA on oocyte health grouped according to study type and the oocyte health parameter that was assessed within each study.** Studies that found an adverse effect in an oocyte health parameter are in orange shaded cells, and studies that found no effect are in blue shaded cells, with increasing intensity of colour representing a higher number of studies. *In vivo* studies indicated with an asterisk utilized an indirect exposure model, i.e. *in utero* or breastmilk exposure. Please note that the presence of a study in the blue shaded cells indicates no adverse effect only for the relevant outcome, not the whole study. Many studies may have reported an adverse effect for a different outcome in the figure or, alternatively, a different outcome that was not covered in this review. Studies that were listed as having no adverse effect above but which reported other adverse effects of BPA on the oocyte, ovary or female fertility outside the scope of this review include Campen *et al.* (2017), Fernández *et al*. (2010), Moore-Ambriz *et al.* (2015), Lebachelier de la Riviere *et al.* (2023), Bloom *et al.* (2011), Chavarro *et al.* (2016), Herez *et al.* (2022), Lin *et al.* (2021), Mínguez-Alarcón *et al.* (2016), Shen *et al.* (2020), and Yenigül *et al.* (2021).

**Figure 5.**
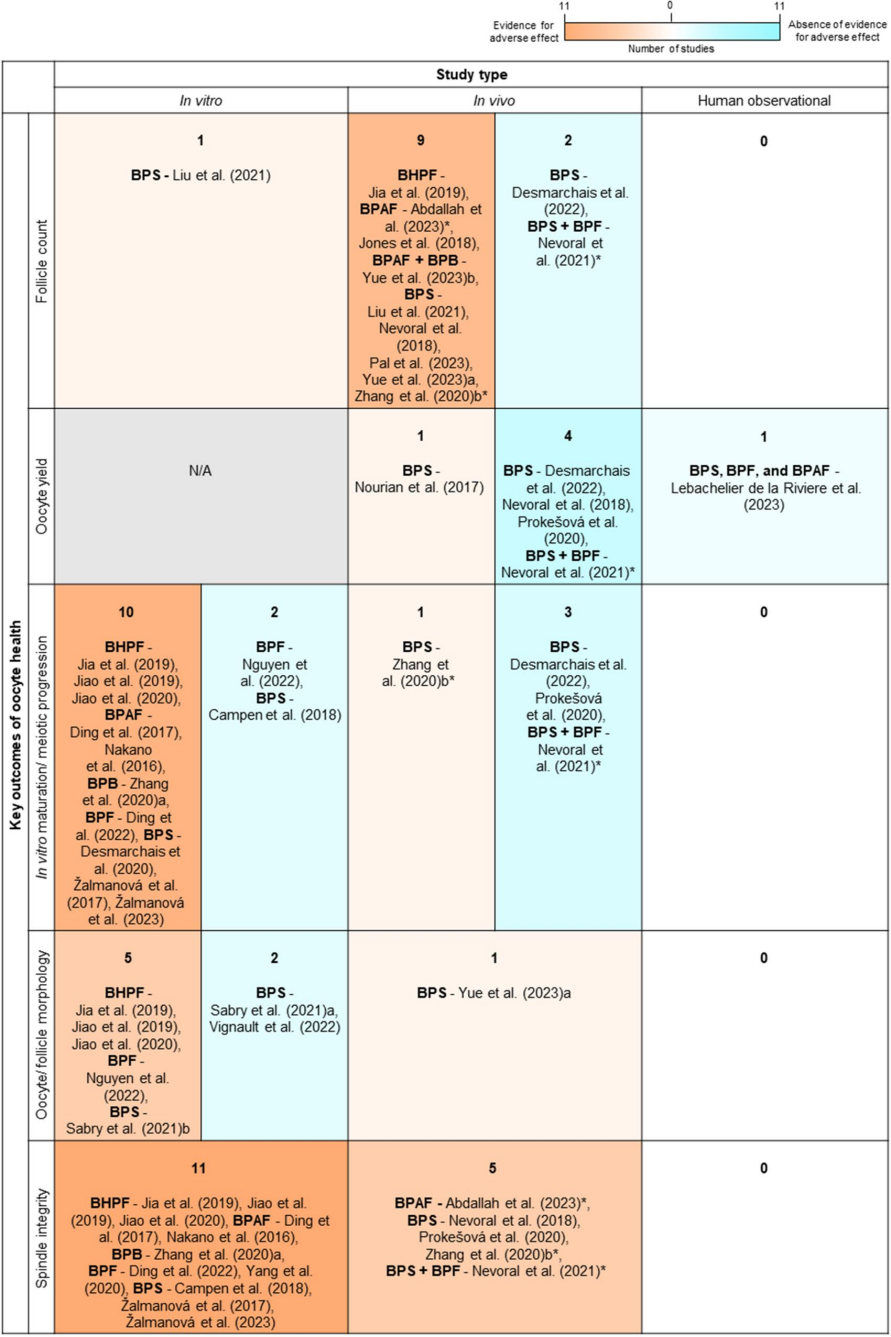
**Studies assessing the effect of BPA alternatives on oocyte health grouped according to study type and the oocyte health parameter that was assessed within each study.** Studies that found an adverse effect in an oocyte health parameter are in orange shaded cells and studies that found no effect are in blue shaded cells. *In vivo* studies indicated with an asterisk utilized an indirect exposure model, i.e. *in utero* or breastmilk exposure. Please note that the presence of a study in the blue shaded cells indicates no adverse effect only for the relevant outcome, not the whole study. Many studies may have reported an adverse effect for a different outcome in the figure or, alternatively, a different outcome that was not covered in this review. Studies that were listed as having no adverse effect above but which reported other adverse effects of BPA on the oocyte, ovary or female fertility outside the scope of this review include Nguyen *et al.* (2022), Sabry *et al.* (2021a), Vignault *et al.* (2022), Desmarchais *et al.* (2022), Nevoral *et al.* (2018), Nevoral *et al.* (2021), Prokešová *et al.* (2020), and Lebachelier de la Riviere *et al.* (2023). Abbreviations: BHPF, fluorene-9-bisphenol; BPAF, bisphenol AF; BPB, bisphenol B; BPF, bisphenol F; BPS, bisphenol S.

Key results of these 107 studies were recorded and summarized, based on the effect seen, in Fig. 4 for BPA and Fig. 5 for the BPA alternatives. Five standardized parameters of oocyte health were developed for assessment in this review based on the US EPA guidelines for reproductive toxicity and standard observable/morphological measures of oocyte health used within Australian IVF clinics, and internationally applicable (EPA, 1996; Rienzi *et al.*, 2012). These included follicle counts (quantitative data), yield of oocytes collected, meiotic capacity, morphology of the oocyte or cumulus-oocyte-complex (COC), and meiotic spindle characteristics. Effects in these categories were considered adverse in this review if they significantly differed from the control group. Seven studies included in this review did not measure any of these parameters and so their findings were not included in Figs 4 and 5. Overall, excluding these studies, 85% (67/80 or 83.8% for BPA and 26/29 or 89.7% for BPA alternatives) of studies that assessed at least one of five parameters, documented an adverse effect.

### *In vitro* studies

Of the studies assessing BPA, 37.2% (32/86) utilized *in vitro* methods. Five studies did not include any assessable parameters aligned to this study. The concentrations of BPA employed within these studies varied vastly, and importantly, 11 studies employed treatment concentrations at or below the *in vitro* equivalent of the US FDA lowest observed adverse effect level (LOAEL) for BPA of 50 mg/kg bw/day (Vandenberg *et al.*, 2013a). The extrapolation of this *in vivo* LOAEL to an equivalent *in vitro* treatment concentration presents some difficulty (Ziv-Gal *et al.*, 2013) due to the vastly different nature of these two exposure models, however the LOAEL equivalent is estimated to be 50 ng/ml or ∼219 nM (Wetherill *et al.*, 2007). Overall, of the 27 *in vitro* studies that included at least one assessable parameter, 96.3% (26) reported at least one adverse effect in response to BPA treatment, with seven (25.9%) studies reporting adverse effects below the estimated *in vitro* LOAEL.

In contrast to BPA, there is currently no widely established LOAEL or *in vitro* equivalent for any BPA alternative included in this review. Of the studies captured in this review assessing BPA alternatives, 18/30 (60%) employed *in vitro* treatment models. One study did not include any assessable parameters aligned to this study, and of those remaining, 16/17 (94.1%) reported an adverse outcome.

The BPA studies most commonly exposed COCs, isolated follicles, or whole ovaries, with only five studies exposing denuded oocytes (cumulus cells removed) to BPA (Mohri and Yoshida, 2005; Eichenlaub-Ritter *et al.*, 2008; Yang *et al.*, 2020; Pan *et al.*, 2021; Ding *et al.*, 2022). These biological samples were obtained from mice (53.1%), cows (18.9%), pigs (9.4%), humans (9.4%), rats (6.3%), and sheep in one instance. Interestingly, whether denuded, within a COC, or remaining within an isolated follicle, oocytes exhibited similar adverse effects of premature meiotic arrest and deformations in spindle morphology when treated with BPA *in vitro*, even when exposed to doses below the LOAEL *in vitro* equivalent (Lenie *et al.*, 2008; Acuña-Hernández *et al.*, 2018; Yang *et al.*, 2020; Pan *et al.*, 2021). Studies assessing BPA alternatives most frequently treated GV oocytes, MII oocytes, or COCs from mice (44.4%), cows (33.3%), pigs (16.7%), or sheep in one instance, with varying concentrations of BPA alternative.

#### Reporting of methodologies and study design

The reporting of methodological characteristics pertaining to sample size amongst *in vitro* studies was inconsistent. Eight (18.6%, 8/43) studies clearly reported their total utilized animal/human specimen numbers for the entirety of the study and across all experiments and/or experimental replicates. Amongst the remaining majority of studies that did not provide complete sample size information, there was variable reporting of experimental replicates. These studies described replicates as either independent (25.6%, 11/43), biological (20.9%, 9/43), or not specified (16.3%, 7/43), rendering specific biological sample size and the repetition of experiments open to reader interpretation in many cases. Eight studies (18.6%, 8/43) did not disclose any information about the replicates undertaken to generate their data, and 14% (6/43) of studies did not specify the oocyte or ovary numbers used. For the BPA studies, 50% (16) included three or more concentrations whilst only five studies included five or more. For studies assessing BPA alternatives, 72.2% (13) included at least three concentrations and two studies included at least five. Additionally, the age or reproductive status of utilized animals was not specified in 25.6% (11/43) of *in vitro* studies.

#### Meiotic progression

The most reported feature of oocyte health within the studies evaluating BPA and BPA alternatives *in vitro* was meiotic progression in 20 and 12 studies, respectively. There were 18/20 (90.0%) and 10/12 (83.3%; including BPS, BPAF, BHPF, BPF, and BPB) studies which reported adverse effects (Figs 4 and 5). All the studies assessing meiotic progression examined the *in vitro* maturation of pre-ovulatory oocytes except for Brieño-Enríquez *et al.* (2011), who cultured fetal oocytes. The studies disclosing adverse effects detailed reduced polar body extrusion rates and variability in stage of premature arrest or atresia.

Three studies in total reported no adverse effects on meiotic progression in COCs from cows and rats for BPA and BPS at doses up to 50 µM, and 0.05 mg/ml or 200 µM for BPF (Campen *et al.*, 2017; 2018; Nguyen *et al.*, 2022).

#### Spindle integrity

Overall, spindle integrity was a frequently reported parameter across the *in vitro* studies (12 for BPA, 11 for BPA alternatives) with all reporting adverse effects including chromosome misalignment or altered spindle dimensions due to BPA or BPA alternative exposure. All five BPA alternatives included in this review were represented in this category. Campen *et al.* (2018) study, covering both BPA and BPS, reported extensive spindle abnormalities in oocytes exposed to almost all concentrations of both chemicals tested; these concentrations were as low as 1 fM (10^−15^ molar) and well below the NOAEL for both bisphenols.

#### Oocyte and follicle morphology

Eleven *in vitro* studies assessed morphology and all 11 recorded adverse morphological outcomes resulting from BPA treatment of COCs or follicles. These commonly reported reduced cumulus cell expansion and, in some cases, darkened granulosa/cumulus cells, however oocyte morphology was rarely described. Five out of seven BPA alternative studies assessing morphology reported adverse effects in COC expansion and darkened cumulus cells for BPF, BPS, and BHPF, and/or cytoplasmic oocyte abnormalities such as granular appearance for BHPF.

#### Follicle counts

All four studies that measured follicle numbers in cultured ovaries identified adverse effects of BPA treatment through delayed germ cell nest breakdown causing reduced primordial follicles in neonatal mouse and rat ovaries (Zhang *et al.*, 2014; Zhao *et al.*, 2014; Zhou *et al.*, 2015; Ganesan and Keating, 2016). In contrast, the only study assessing BPS on mouse germ cell nest breakdown demonstrated the opposite effect whereby BPS treated ovaries in culture contained reduced numbers of germ cell cysts and increased numbers of follicles compared to the control group (Liu *et al.*, 2021).

### *In vivo* studies

There were 35 studies assessing BPA *in vivo* included in this review; 22 (62.9%) of these were carried out in mice, 10 (28.6%) in rats, and 1 each in macaques, gerbils, and sheep. As mentioned earlier, the LOAEL for BPA is 50 mg/kg bw/day; this dose informs the subsequent no observed adverse effect level (NOAEL) of 5 mg/kg bw/day, and finally the TDI, another 100 times lower at 0.05 mg/kg bw/day (FDA BPA Joint Emerging Science Working Group, 2014b). Of the 35 studies in this review, 30 (85.7%) *in vivo* BPA studies reported an adverse effect in at least one of the five assessable parameters. Each of these studies documented effects at or below the LOAEL, with a further 71.4% below the NOAEL, and 40% reporting effects at or below the TDI.

There were 13 studies which measured the *in vivo* impacts of BPA alternatives including BPS alone (eight studies), BPS alongside BPF (one study), BPAF alongside BPA, BPB, or BADGE (one study each), and BHPF (one study). Of these 13 studies, 12 (92.3%) observed at least 1 adverse outcome, and all 9 BPS studies that documented adverse effects recorded these below the European FSA reproductive toxicity limit of 180 mg/kg bw/day (EFSA *et al.*, 2020). The 12 *in vivo* models that resulted in adverse outcomes were conducted in mice or, in one case, hamsters. The only study that reported no adverse effects delivered very low levels of BPS at 4 or 50 µg/kg bw/day in the diet of primiparous sheep for at least 3 months and reported no changes in follicle counts, oocyte yield, and meiotic capacity (Desmarchais *et al.*, 2022). Only one *in vivo* study compared BPA with an alternative (BPAF) and the same adverse effect of increased atretic follicles was recorded across all BPA and BPAF treated groups regardless of dose (Jones *et al.*, 2018).

We identified 16 studies (14 for BPA, 1 for BPS, 1 for BPAF and BADGE) that delivered bisphenols to mothers during gestation (perinatally), and subsequently measured the effects of *in utero* exposure on offspring fertility. In six cases, BPA or a combination of BPS and BPF was delivered to mothers during lactation to assess indirect exposure through breastmilk to neonates and their reproductive health outcomes. Of all the perinatal exposure studies, 92.9% (13/14) that assessed a standardized parameter of offspring oocyte health reported an adverse effect regardless of the timing, dose (at the LOAEL, NOAEL, or TDI), and duration of exposure during gestation. These adverse effects most commonly culminated in numerous changes to follicle numbers in F1 offspring (11 studies), specifically reducing primordial follicle count in seven of these studies. There were also four studies that reported effects on the meiotic spindle including spindle aberrations, chromosome misalignment, and aneuploidy in F1 generation oocytes. Similar reductions in primordial follicle count or perturbance of ovarian cyst breakdown were further observed in four instances, in the F2 generation or skipping to the F3 generation resulting from F0 exposure to low-dose BPS, BPA alone, or combined with a high fat diet or other EDCs found in plastic (Nilsson *et al.*, 2012; Berger *et al.*, 2016; Zhang *et al.*, 2020b; Huang *et al.*, 2022).

#### Reporting of methodologies and study design

Twelve of the 47 *in vivo* studies (25.5%) did not include sufficient details regarding the number of animals used in each treatment group within their dosing regimen. Oral delivery of BPA/BPA alternatives was employed in 68.1% (32/47) of all studies, with three or more doses delivered in 38.3% (18/47) of cases. Extending this approach to study parameters, six studies could not be analyzed for their results due to the lack of any comparable endpoints.

#### Follicle counts

Changes in follicle number was the most reported outcome in response to *in vivo* BPA and BPA alternative exposure, with 22/24 (91.7%) and 9/11 (81.1%) studies reporting an adverse effect, respectively. For BPA, 13 studies observed decreased primordial follicle numbers, indicating a depleted ovarian reserve resulting from direct or *in utero* exposure. Alongside this, there were two reports of increased primordial follicle numbers as a result of either mouse neonatal exposure or adult gerbil exposure followed by ageing to 18 months (Karavan and Pepling, 2012; Ruiz *et al.*, 2023). In three cases, a reduction in primordial follicle numbers was identified in either the F2 or F3 generation of rats or mice from F0 mothers exposed during gestation and/or lactation (Nilsson *et al.*, 2012; Berger *et al.*, 2016; Huang *et al.*, 2022). However, in two of these studies, the effects of BPA were observed in combination with other plastic derived endocrine disrupting chemicals or a high-fat diet in the F0 generation (Nilsson *et al.*, 2012; Huang *et al.*, 2022). Across the total 22 studies recording changes in follicle number, 9 reported increased atretic follicle numbers in BPA-treated groups, and 4 documented increased numbers of multi-oocyte follicles, however, the quantity of other follicle types were altered in an inconsistent manner.

In contrast, only two BPA alternative studies recorded a change in primordial follicle counts. These studies presented opposing changes of decreased primordial follicle numbers resulting from BPAF exposure of adult mice (Yue *et al.*, 2023b) or increased primordial follicle numbers in F1 offspring of BPS exposed pregnant mice for four days mid-gestation, however, these effects were not maintained in the F2 offspring (Zhang *et al.*, 2020b). Notably, five studies observed an increase in atretic follicle numbers due to oral delivery of BPS, BHPF, BPAF, or BPB to sexually mature mice or hamsters, or *in utero* F1 exposed mouse offspring. Similar to BPA, the BPA alternative studies reported a number of varying observations in quantities of other follicle stages.

#### Oocyte spindle integrity

The integrity of oocyte spindles was found to be compromised in six out of seven *in vivo* BPA studies, and in all five *in vivo* BPA alternative studies (four BPS, one BPF, one BPAF + BADGE). BPA exposures below the LOAEL in a variety of models led to increased rates of aberrant spindles and/or misaligned chromosomes in three studies (Hunt *et al.*, 2003; Muhlhauser *et al.*, 2009; Zhang *et al.*, 2017), as well as abnormal MI spindles (Chao *et al.*, 2012), aneuploidy (Susiarjo *et al.*, 2007), and premature centrosome separation (Pacchierotti *et al.*, 2008). Spindle alignment was impacted in all studies by low dose oral BPS and BPF exposure in mice for durations of between 4 days and 4 weeks. Three studies also documented chromosomal abnormalities (misalignment, aneuploidy) resulting from BPS, BPAF, or BADGE exposure in F1 offspring of exposed mothers or in neonates feeding from exposed mothers (Zhang *et al.*, 2020b; Nevoral *et al.*, 2021; Abdallah *et al.*, 2023).

#### Meiotic progression

Two out of four studies investigating *in vitro* meiotic progression of pre-ovulatory oocytes retrieved from young mice exposed to BPA orally or hypodermically revealed reduced germinal vesicle breakdown rates under the NOAEL (Chao *et al.*, 2012) or reduced polar body extrusion rates under the LOAEL (Zhang *et al.*, 2017). Likewise, Zhang *et al.* (2020) observed decreased rates of both germinal vesicle breakdown and polar body extrusion in *in vitro* matured oocytes retrieved from F1 mice exposed to low-dose BPS *in utero*. Three other studies assessed the meiotic progression of oocytes retrieved from sheep or mice orally exposed to BPS and BPF at or below the TDI (0.05 mg/kg bw/day). These three studies reported no changes in the *in vitro* maturation rates, regardless of the exposure model (acute, chronic, or indirect neonatal exposure through breast milk) or the presence or absence of cumulus cells (Prokešová *et al.*, 2020; Nevoral *et al.*, 2021; Desmarchais *et al.*, 2022).

#### Oocyte and follicle morphology

All four *in vivo* BPA studies that reported on follicle and/or oocyte morphology observed a range of adverse effects. These included cystic dilation of follicles with decreased sparse granulosa cells (Lin *et al.*, 2021), increased oocyte diameter (Chao *et al.*, 2012), presence of granules in the oocyte cytoplasm (Liu *et al.*, 2017), and decreased zona pellucida thickness, oocyte volume, and nuclear volume in antral follicles (Mansoori *et al.*, 2013). In three out of the four studies, sexually mature mice or rats were exposed to BPA orally.

We identified a single study examining the effects of oral 300 µg/kg bw/day BPS on the uterus and ovary of adult mice. This study recorded unclear oocyte structure within follicles, compromised zona pellucida appearance, and atrophy of immature follicles (Yue *et al.*, 2023a).

#### Oocyte yield

Only one out of six studies recorded a reduced yield of healthy oocytes due to 10 mg/kg bw/day BPA exposure for 42 days in mice (Hu *et al.*, 2018). Similarly, one out of five BPA alternative studies documented a decrease in mean oocyte yield with increasing BPS dose for five doses up to 100 µg/kg bw/day for 21 days in mice (Nourian *et al.*, 2017).

### Clinical studies

There were 21 observational studies that assessed BPA levels in patients attending fertility clinics; one of these studies assessed BPS, BPF, and BPAF alongside BPA (Lebachelier de la Riviere *et al.*, 2023). These studies collected urine, blood, and/or follicular fluid from patients to measure the amount of BPA present and correlated this with patient outcomes. The parameters of interest recorded in these studies were oocyte yield and antral follicle count. There were four studies that examined these correlations in the context of additional lifestyle factors including folate intake, soy intake, plastic container use, and bottled water intake (Chavarro *et al.*, 2016; Mínguez-Alarcón *et al.*, 2016; Aftabsavad *et al.*, 2021; Yenigül *et al.*, 2021). No studies reported on *in vitro* oocyte maturation, recorded descriptive morphological data on the oocytes collected, or examined the spindle integrity of MII oocytes.

Participant numbers across the 21 studies ranged from 44 to 450, with an age range of 18–46.7 years old. All 10 studies conducted in the US, one Korean, and one Greek study documented a mean or median participant age between 35 and 36 years of age, except for a mean age of 38 for the Greek study participants with tubal factor infertility (mean of total participants not provided) (Mina *et al.*, 2022). Studies from other countries including China, Iran, Iraq, Poland, France, and Turkey had younger participants with a mean or median age between 27 and 33, however one Iranian study did not provide statistical data on patient age (Poormoosavi *et al.*, 2019).

In 76.2% (16/21) of studies, patient populations were undergoing autologous IVF or ICSI cycles for a variety of infertility factors including female factor, male factor, combined, and unexplained infertility. In addition to these studies, Zhou *et al.* (2017) only assessed patients with polycystic ovarian syndrome (PCOS), Shen *et al.* (2020) assessed those with tubal factor infertility, and Mina *et al.* (2022) studied participants with either of these diagnoses. Two studies only included patients undergoing ICSI that had either an unexpected poor ovarian response (Aftabsavad *et al.*, 2021) or unexplained infertility (Yenigül *et al.*, 2021). Eight of the 21 studies were undertaken as part of the Environment and Reproductive Health (EARTH) study, an ongoing prospective cohort study carried out in Massachusetts General Hospital Fertility Center between 2004 and 2012 to evaluate environmental and dietary determinants of fertility.

Overall, 52.4% (11/21) of the clinical studies recorded at least one adverse effect. Of the 18 studies that reported on oocyte yield, nine (50%) documented reduced total and/or mature (MII) oocyte yield associated with higher levels of BPA in urine, serum, or follicular fluid. Conversely, eight studies conducted antral follicle counts of participants and three of these recorded a negative association between increasing BPA concentration and antral follicle numbers (Souter *et al.*, 2013; Zhou *et al.*, 2017), though one of these studies recorded a *P* value of 0.051 for this parameter (Aftabsavad *et al.*, 2021). Adverse effects were reported in some of the studies assessing specific subsets of patient populations seeking ART procedures in 6 out of 11 instances, including those undergoing ICSI (Fujimoto *et al.*, 2011), those with tubal factor infertility (Shen *et al.*, 2020; Mina *et al.*, 2022), those with poor response to ovarian stimulation (Aftabsavad *et al.*, 2021), or those with PCOS (Zhou *et al.*, 2017). The studies that revealed associations between oocyte or follicle parameters with BPA were performed on patient cohorts from China (three studies), USA (three studies), Iran (two studies), Greece (one study), and Poland (one study).

There were an equal number of studies (9/18, 50%) that reported no correlation between BPA concentrations measured in patient samples and oocyte yield, and five studies that failed to record a correlation with antral follicle count. These studies were primarily based on patient cohorts from the USA (six studies), alongside four studies from Korea, Iraq, France, and Turkey.

Five studies assessed oocyte yield and antral follicle count together; of these, two reported no changes in both parameters (Bloom *et al.*, 2011; Yenigül *et al.*, 2021), two studies documented an association with oocyte yield but not antral follicle count (Shen *et al.*, 2020; Lin *et al.*, 2021), and one revealed associations with both parameters (Aftabsavad *et al.*, 2021). Despite Bloom *et al.* (2011) reporting no correlations between BPA concentrations and total oocyte yield or follicle counts, another study using the same patient cohort by Fujimoto *et al.* (2011) reported negative correlations between serum BPA concentration and mature MII oocyte yield in nine Asian women undergoing ICSI.

A recent study from France was the first to assess conjugated BPS, BPF, and BPAF (alongside BPA) in follicular fluid and correlated these levels with oocyte yield. This study did not observe a correlation with any bisphenol, however they only examined this by grouping participant samples based on whether bisphenols were detectable or undetectable and not by concentration (Lebachelier de la Riviere *et al.*, 2023).

**Figure 6.**
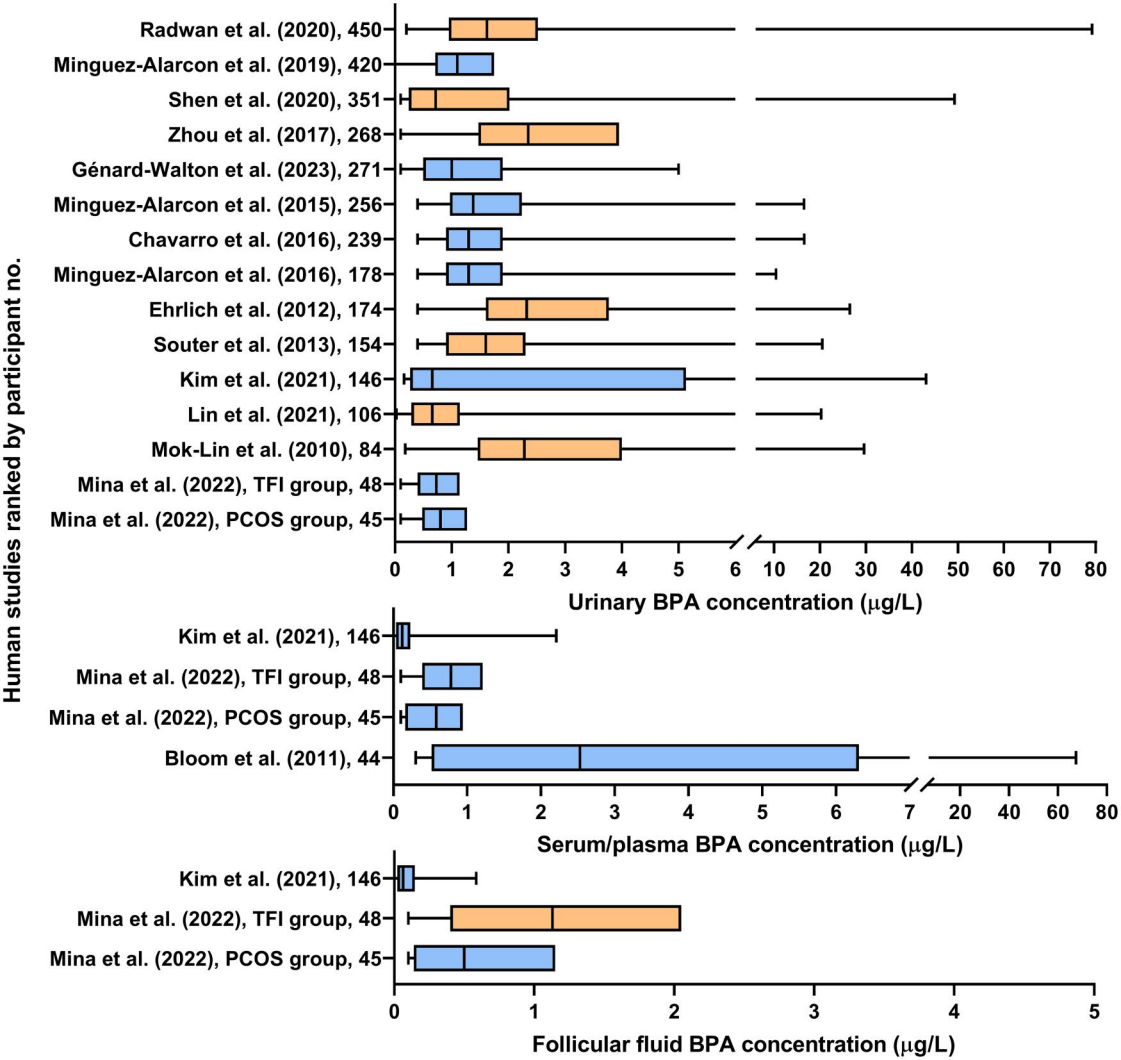
**Box and whisker plot of 15 human observational studies ranked by participant number, displaying urinary, serum, or follicular fluid BPA concentrations of the participants.** BPA concentration data presented includes the median, quartile 1, quartile 3, the minimum detected concentration or limit of detection, and maximum detected concentration where available. Orange indicates that the study found an adverse effect associated with BPA concentration, blue indicates the absence of an adverse effect. Six studies did not provide adequate information on the bisphenol concentrations of participant samples and were not included in this figure.

The levels of detected BPA in patient samples varied greatly from study-to-study, even amongst studies from the same country with sample BPA concentrations below the limit of detection (commonly 0.1–0.4 µg/l for urine) ranging from 0% up to 56.8% (Kim *et al.*, 2021) and 88.1% (Lebachelier de la Riviere *et al.*, 2023). Additionally, for the BPA alternatives, 87% (BPS) and 98.6% (BPF) of samples were below the limit of detection, and BPAF was undetectable across all samples (Lebachelier de la Riviere *et al.*, 2023). The median adjusted urinary BPA concentrations ranged from 0.66 to 2.35 µg/l, and the maximum detected BPA in urine ranged from 10.45 to 79.19 µg/l (Fig. 6). Only three out of five studies that assessed serum/plasma, and two out of seven studies that assessed follicular fluid, reported median BPA concentrations; these were 0.012–2.53 µg/l and 0.063–1.13 µg/l, respectively (Bloom *et al.*, 2011; Kim *et al.*, 2021; Mina *et al.*, 2022).

Overall, only 7 out of 21 studies (33%) provided a full range of standard statistical parameters on BPA levels in their study population including quartiles or percentiles, means, standard deviations, maximum concentrations, the limit of detection, and the percentage of the population that fell below this limit. A further eight studies provided adequate information missing one or two of these details, and six studies provided very minimal, insufficient data on BPA concentrations across their participants and as such are not included in Fig. 6.

## Discussion

This scoping review was designed to determine the extent of knowledge on BPA and BPA alternatives to date, and the effects of these chemicals on oocyte health. Additionally, we sought to provide insight into current regulations, or lack thereof, for BPA and its analogues established by the US FDA and mirrored across many countries, and the suitability of these for oocyte health and female fertility. Specific endpoints that measure oocyte health and related ovarian parameters such as those documented in this review have been neglected within US FDA guideline studies and in the CLARITY-BPA program, and are generally understudied compared to other endpoints (Tyl *et al.*, 2002, 2008; FDA BPA Joint Emerging Science Working Group, 2014b; Camacho *et al.*, 2019). It is of great importance for human health that the quality of the oocyte (a cell capable of governing reproductive success, pregnancy success, and offspring health) is considered by food safety regulatory bodies worldwide when deciding safe exposure of toxicants within our diet. Importantly, this review highlights only one example of the potential thousands of endocrine disrupting chemicals (EDCs) humans are exposed to through diet or within the environment daily that remain largely untested and unregulated (Futran Fuhrman *et al.*, 2015).

### Trends in adverse effects caused by bisphenols on oocyte health

In preparing this review, it was found that 85% (85/100) of studies that assessed at least one of five oocyte health parameters (including follicle counts, oocyte yield, oocyte meiotic capacity, oocyte/follicle morphology, and spindle integrity) recorded an adverse effect. Overall, all bisphenol analogues examined in these studies displayed similar patterns in terms of the impact they exerted on the oocyte *in vitro* and *in vivo*. *In vitro*, the most frequently affected parameters of oocyte health were meiotic progression, spindle integrity, and oocyte/COC morphology. *In vivo*, resulting from direct or *in utero* exposure, the most affected parameters were follicle counts followed by spindle integrity for BPA and its alternatives. Adverse outcomes in oocyte spindle integrity, including misaligned chromosomes and abnormal spindle morphology, were conserved between the two exposure models. Patterns in adverse effects that have emerged from the synthesis of data in this review provide insight into the mechanisms and extent of damage BPA can cause to the oocyte. These data also provide concerning evidence that the analogues of BPA can elicit the same detrimental effects, however the field would greatly benefit from further studies of these alternatives incorporating additional endpoint analyses. In this context, we noted that the majority of the *in vivo* studies that assessed a BPA alternative focused solely on BPS (8/13), so more *in vivo* studies assessing the range of other BPA alternatives are warranted.

The evidence for adverse outcomes associated with BPA exposure gleaned from human observational studies revealed different trends. For instance, the studies that assessed follicle counts were only able to quantify antral follicles via ultrasound, where three out of eight studies reported an associated decrease in antral follicles with BPA exposure. The outcomes of these human studies cannot be directly compared to the other study types in this review where a broader range of follicle types could be quantified, and the most common effect was reduced primordial follicles.

Decreased oocyte yield, the most documented parameter in human studies, was associated with increased urinary BPA in half of the studies. The human oocytes retrieved were largely of unreported quality with respect to both meiotic spindle integrity and morphology. Aftabsavad *et al.* (2021) did report decreased ‘oocyte quality’ associated with higher follicular fluid BPA concentration, however the authors provided no indication of how oocyte quality was assessed in their study design. In contrast, most *in vivo* animal studies found no association between BPA exposure and oocyte yield, however, five studies that documented unchanged oocyte yield subsequently recorded that these oocytes had increased chromosome abnormalities or spindle alignment issues after BPA, BPS, or BPF exposure. Utilizing discarded human oocytes from patients who provided urine samples would enable assessment of meiotic spindle defects or oocyte morphology providing valuable information on the impacts of bisphenols on human oocyte quality. Alternatively, parameters of embryo development and quality could be used as an indicator of oocyte quality; this was included in a number of human studies (Mínguez-Alarcón *et al.*, 2016; Radwan *et al.*, 2020; Shen *et al.*, 2020), however, embryo outcomes were not a parameter chosen for assessment in this scoping review. It should therefore be emphasized that the strength of evidence presented by the oocyte-specific parameters herein does not reflect or predict potential adverse embryo or pregnancy outcomes reported in these clinical studies.

### *In vitro* studies

The *in vitro* studies considered in this review were primarily mechanistic, being designed with the aim of investigating and understanding how bisphenols alter oocyte or follicle health parameters. As such, the bisphenol concentrations used in these studies are generally not reflective of actual exposure levels in human populations. *In vitro*, the metabolic processing that alters bisphenol bioactivity is removed (Wetherill *et al.*, 2007; Thayer *et al.*, 2015) and chronic exposure is simply not possible to imitate, particularly when culturing oocytes. Despite these limitations, seven BPA studies recorded low dose adverse effects below the equivalent *in vitro* LOAEL of 50 ng/ml. It is clear from the *in vitro* data summarized in this review that the oocyte is susceptible to damage from bisphenol exposure at very low concentrations *in vitro*.

The mechanistic investigations of *in vitro* studies, despite not being the focus of this review, give valuable insights into the reasons underlying the profound impacts of bisphenol exposure on oocyte health. Bisphenols can act directly on receptors in an agonistic or antagonistic manner, interfering with receptor signalling pathways and hormone synthesis. It has been demonstrated in multiple instances that BPA and its analogues including BPS, BPB, BPF, and BPAF have comparable or superior estrogenic agonist potencies on human estrogen receptors α and β (Kojima *et al.*, 2019; Durcik *et al.*, 2022). Many bisphenols also possess similarities in their effects on the androgen receptor, pregnane X receptor, constitutive androstane receptor, and the glucocorticoid receptor (Kojima *et al.*, 2019). This functional comparability in the actions of BPA and BPA alternatives provides an explanation for the similarities in their effects on the oocyte and follicle and emphasizes the urgency of revising their safety. In addition to these direct impacts on receptors, bisphenols can also have indirect effects resulting in induction of apoptosis, oxidative stress, and inflammation in many components of the female reproductive system including cells within the ovary (Pan *et al.*, 2024; Wang *et al.*, 2024). Oxidative stress, and in some cases, apoptosis, was induced by BPA or BPA alternative exposure in numerous *in vitro* studies that recorded standardized oocyte health parameters reviewed herein. These resultant impacts of increased oxidative stress and dysregulated apoptosis on cultured neonatal ovaries (Zhang *et al.*, 2014; Zhou *et al.*, 2015), pre-antral follicles (Li *et al.*, 2022), cumulus-oocyte complexes (Park *et al.*, 2018; Li and Zhao, 2019; Jiao *et al.*, 2020), and oocytes (Ding *et al.*, 2017, 2022) are key explanatory factors contributing to the understanding of how bisphenols have detrimental impacts on follicle and oocyte development/maturation and on oocyte quality.

A characteristic observed across some studies on bisphenols was non-monotonicity; that is, a non-linear relationship between dose and effect, that can often be ‘U’ shaped (Vandenberg, 2014; Eladak *et al.*, 2015). This phenomenon has been observed far beyond EDCs and toxicological contexts, in pharmacological and even biological responses to natural hormones and hormonal drugs (Calabrese and Baldwin, 2001; Vandenberg *et al.*, 2012). Indeed, some *in vitro* studies reviewed here displayed non-linear dose responses to BPA and its analogues, whereby low concentrations produced more severe adverse effects than that associated with higher concentrations (Acuña-Hernández *et al.*, 2018; Campen *et al.*, 2018). In the case of non-monotonicity, it can be argued that there is no safe low dose, as low-dose effects can be just as harmful, if not more harmful, than higher doses. Dose response dynamics are further complicated by concurrent exposure to other EDCs that can produce synergistic, additive, or antagonistic effects on various receptors and hormone axes when combined (Hamid *et al.*, 2021). Compounds with endocrine disrupting properties are greatly abundant in food and daily life, including phytoestrogens such as genistein, other chemical components of plastic such as phthalates, and pharmaceutical drugs such as antibiotics. Taken together, these factors create a challenge for BPA regulation by governing bodies such as the US FDA who hold BPA to the standard of a linear dose-response, leading to the dismissal of published data that demonstrates non-monotonic effects (Vom Saal and Vandenberg, 2021). For change in the regulation of bisphenols to occur, a paradigm shift in the understanding of bisphenol mechanisms led by *in vitro* studies is required. Accordingly, the performance of additional studies is recommended to increase our understanding of the mechanisms by which bisphenols act on the oocyte alone and in combination with other EDCs to drive non-monotonic dose responses.

### *In vivo* studies

The endocrine disrupting nature of bisphenols leads to many of their impacts being sex-specific. While this review addresses those impacts on the female reproductive system, more specifically the oocyte and follicle, it should remain of high importance that bisphenols can impact all aspects of human reproduction, including male fertility. Valuable *in vitro* and *in vivo* studies have revealed that bisphenols can disturb the function of testicular mitochondria, dysregulate testicular hormone production by affecting Leydig and Sertoli cells, interfere with spermatogenesis, affect sperm leading to reduced embryo cleavage and blastocyst formation, and alter testis morphology (Adegoke *et al.*, 2020; Cariati *et al.*, 2020; Li *et al.*, 2020; Ryu *et al.*, 2023). These impacts are underpinned by similar underlying mechanisms as those that affect the female reproductive system such as interfering with receptor signalling, increased oxidative stress and apoptosis, and induction of epigenetic modifications. The consideration of the sex-specific impacts of bisphenol exposure together provides added insight into the mechanisms responsible and should be employed in human risk assessments as these effects ultimately culminate in same outcomes of compromised reproduction/fertility and offspring health.

The majority (33/35, 94.3%) of *in vivo* studies reviewed herein were performed in rodent models. The relevance of rodent models to human risk assessment of BPA exposure has been trivialized by regulatory bodies such as the FDA, maintaining the perspective that rodent toxicity data is not highly relevant to humans. This stance was generated by the findings of two studies performed by Völkel *et al.* (2002) in a small number of human volunteers, concluding that orally ingested BPA is efficiently converted to its less active form, BPA glucuronide, *via* first-pass metabolism in a complete manner before it is rapidly excreted in urine resulting in minimal burden to the human body. It was also highlighted that this process occurs rapidly in humans compared to rats, indicative of major species differences and that highly active unconjugated BPA detected in human biological samples is likely due to contamination (Völkel *et al.*, 2008). These findings and subsequent studies led by the FDA have been used to defend decisions to maintain the current BPA TDI, insist that human unconjugated BPA exposure is negligible, and dismiss low-dose exposure studies using rodent models. There are, however, several human biomonitoring studies that provide opposing evidence, demonstrating that glucuronidation is not a complete process in humans for BPA or its alternatives (Thayer *et al.*, 2015; Karrer *et al.*, 2018). Indeed, there is also evidence suggesting high similarity in the unconjugated bioavailability of BPA in adult humans, rhesus monkeys, rats, and mice despite differences in the routes of elimination (Taylor *et al.*, 2011; Thayer *et al.*, 2015). Additionally, there are other important factors that must be considered when modelling human exposure and comparing relevance of model species. One of these factors is developmental maturity, as neonatal glucuronidation capacity, or the ability for the neonate to efficiently metabolize bisphenols, is not yet fully functional, carrying major implications for fetal and neonatal exposure (Ginsberg and Rice, 2009; Karrer *et al.*, 2018). Another key factor is the route of exposure, as different bisphenol exposure routes lead to variations in the way it is metabolized. For example, transdermally absorbed BPA has a longer half-life than orally absorbed BPA, as it is not subject to first-pass metabolism (Vandenberg *et al.*, 2013b; Sasso *et al.*, 2020). Taken together, it is evident that bisphenol pharmacokinetics have many intricacies, and whilst species specific differences occur, these are not significant enough to disregard low-dose rodent exposure models. The *in vivo* rodent data in this review provides a wealth of valuable information, however like all scientific findings, this should be interpreted alongside data from other species and study designs.

Due to the aforementioned metabolic differences in the developing neonate, bisphenol exposure during gestation and neonatal development is of high concern. Our review identified 13 studies that delivered bisphenols to mothers during gestation (perinatally) and reported an adverse effect in a standardized oocyte health parameter in offspring. These adverse effects were overwhelmingly observed as changes to follicle numbers in F1 generation ovaries, commonly reducing primordial follicles, and dysregulating oocyte spindle alignment, indicative of detrimental impacts to follicle formation and oogenesis. Disturbances to the ovary, again, most often reduced primordial follicles, were further observed in the F2 generation or skipping to the F3 generation resulting from F0 exposure to bisphenols alone or in combination with other insults, demonstrating the sensitivity of fetal germ cells to bisphenols and the transgenerational implications of bisphenol exposure on fertility (Nilsson *et al.*, 2012; Berger *et al.*, 2016; Zhang *et al.*, 2020b; Huang *et al.*, 2022). A mechanism through which BPA exposure has been implicated in causing multigenerational and transgenerational effects on hormonal regulation and fertility outcomes is epigenetic modification. Epigenetic modifications are chemical changes to chromatin including DNA methylation, histone modification, and modulation of non-coding RNA. These processes result in the alteration of gene expression without causing changes to the DNA sequence, and importantly, are heritable (Santangeli *et al.*, 2017). Several studies captured in this review also identified epigenetic changes in oocytes exposed to bisphenols *in vitro*, *in vivo*, and in F1 offspring (*in utero*). These included overall methylation changes to DNA, including imprinted genes, and differential methylation of histones (Trapphoff *et al.*, 2013; Wang *et al.*, 2016; Prokešová *et al.*, 2020; Zhang *et al.*, 2020a,b). This evidence of multigenerational and transgenerational effects alongside epigenetic disturbance in the ovary and oocyte could manifest in hugely detrimental long-term impacts on future generations, and urgently warrants further investigation.

A large proportion of the reviewed *in vivo* studies assessed and reported ‘low dose’ effects, that is, effects at doses below the BPA NOAEL of 5 mg/kg bw/day and reflective of human exposure. Equivalently low doses of BPA alternatives were employed yielding similar impacts, despite their supposed low reproductive toxicity reported by regulatory organizations, e.g. European BPS NOAEL is 180 mg/kg bw/day (EFSA *et al.*, 2020).

The US FDA conducted three reviews between 2009 and 2013 in which they evaluated available ‘low dose’ literature assessing outcomes of BPA below the NOAEL of 5 mg/kg bw/day (FDA BPA Joint Emerging Science Working Group, 2011, 2013, 2014a). Eight *in vivo* studies included in this scoping review are evaluated in these US FDA reviews (Adewale *et al.*, 2009; Fernández *et al.*, 2010; Rodriguez *et al.*, 2010; Rivera *et al.*, 2011; Chao *et al.*, 2012; Hunt *et al.*, 2012; Karavan and Pepling, 2012; Signorile *et al.*, 2012). Despite all eight studies reporting adverse effects, the US FDA dismissed the results of seven of these studies due to a lack of clarity in the reporting of the experimental design and methods, assessing less than three doses or less than 10 replicates, criticism of experimental design, and/or lack of ‘meaningful’ endpoints. The current review identified an additional 18 studies on BPA that have observed low-dose adverse effects *in vivo*, many of which have been published in the subsequent 10 years since the release of the latest US FDA review. Alongside these US FDA reviews, the US government initiated a series of studies with independent academic collaborators termed the CLARITY-BPA program to corroborate findings from regulatory guideline studies and academic experimental studies. Beginning in 2012, this was collectively the largest animal study conducted on BPA and its long-term effects (Camacho *et al.*, 2019). This was monumental for BPA research, and reported adverse effects in various organs, including the uterus and ovary, at doses well under the NOAEL. The evidence from this study drove academic collaborators to recommend tighter restrictions on BPA including a new TDI of 2.5 ng/kg bw/day, a mere 0.005% of the current TDI. This recommendation was controversially rejected by the US FDA based on the assumption that human BPA exposure is negligible (Vom Saal and Vandenberg, 2021).

In contrast, the European FSA 2023 review of BPA concluded that it was likely that BPA caused female reproductive toxicity based on the animal evidence, some of which is again included in this review (Moore-Ambriz *et al.*, 2015; Berger *et al.*, 2016; Santamaría *et al.*, 2016; Lambré *et al.*, 2023). More specifically, they concluded there are likely effects on ovarian histology after developmental and adult exposure to BPA, and effects on follicle counts after adult exposure. These conclusions are echoed in the present review. Based on this European FSA review, a new TDI of 0.2 ng/kg bw/day was established for Europe, with concerns for the health of consumers with average and high exposure to BPA exceeding this TDI (Lambré *et al.*, 2023).

Based on the balance of evidence from *in vivo* studies considered in this review in tandem with the European FSA conclusions, it appears timely to recommend that the current BPA NOAEL, risk assessment strategies, and risk management strategies should be reconsidered by organizations such as the US FDA. Further supporting this recommendation, bisphenols and more broadly EDCs, pose a number of challenges in the context of traditional risk assessment of chemical contaminants. Some of these challenges include chronic human exposure occurring in combination with other EDCs, the unknown combinatorial effects of EDC mixtures, latent transgenerational effects, non-monotonicity, and effects caused by EDCs not always being categorically adverse. Moreover, the definition of an ‘adverse effect’ is not universally defined or clear within every context and there appears to be little consensus on what endpoints are most suitable in EDC toxicological studies (Futran Fuhrman *et al.*, 2015). This review identifies strong evidence trends from *in vivo* animal studies that substantiate the adverse effects of BPA, and emerging evidence for its analogues, on oocyte quality in low dose contexts. Taken together, these factors encourage us to closely evaluate the way in which study results are interpreted, what is classified as an adverse effect for the oocyte, and the very definition of reproductive toxicity in an *in vivo* context amongst food safety governing bodies internationally.

### Human observational studies

Despite the measurement of urine, serum, and follicular fluid BPA concentrations in the studies included in this review and within the wider population, urine sample data was the most robust, frequently sampled, and reported in detail, so will be the primary focus of the following discussion. Due to the large inconsistencies in reporting of BPA concentrations in follicular fluid in the included studies, and the inability to compare these follicular fluid data to the wider healthy human population, these data will not be discussed. The median concentrations of urinary BPA observed in the included observational studies was 0.66–2.35 µg/l (or µg/g of creatinine), which is comparable to concentrations in general populations across different continents including Asia, North America, and Europe where the median creatinine-adjusted concentration is 1.36–2.41 µg/g (Zhang *et al.*, 2011; Sarigiannis *et al.*, 2016; Colorado-Yohar *et al.*, 2021). A 2018 study used available global urinary BPA data to back-calculate the estimated daily intake of BPA for adults and children (Huang *et al.*, 2018). Global average daily intakes of BPA were calculated to be 38.78 ng/kg bw/day in adults and 51.74 ng/kg bw/day in children; exposures that exceed the European FSA recommended TDI of 0.2 ng/kg bw/day (Lambré *et al.*, 2023). Other studies that have used median human blood/serum concentrations to calculate predicted exposure have however obtained much higher values, up to 500 µg/kg bw/day (Vandenberg *et al.*, 2007).

Once ingested, BPA is metabolized and eliminated from the body via conjugation enzyme systems in the liver and gastrointestinal tract. Once converted to its conjugated form, most commonly BPA glucuronide, BPA is considered less biologically active and hence less of a health risk, however the more biologically active unconjugated or ‘free’ BPA has still been detected, albeit to a lesser extent, in urine and blood/serum (Vandenberg *et al.*, 2010; Thayer *et al.*, 2015). This detection of unconjugated BPA in urine importantly aligns with literature reporting the incomplete first pass metabolism of BPA in humans, rhesus monkeys, and mice, thus prolonging exposure to the harmful, more biologically active BPA form (Thayer *et al.*, 2015; Taylor *et al.*, 2011). The studies included in this review primarily reported total BPA except for Lebachelier de la Riviere *et al.* (2023) who measured conjugated bisphenols in follicular fluid, and Bloom *et al.* (2011) who analysed unconjugated serum BPA.

There are benefits and disadvantages for the use of different bodily fluids to measure BPA; for example, phenols were found to be more stable within blood/serum compared to urine samples during storage, however BPA is more rapidly metabolized and cleared from the human circulation (Völkel *et al.*, 2002; Vandenberg *et al.*, 2010). Despite their differences, a meta-analysis of BPA concentrations in adult human populations from 14 studies found that equivalent BPA concentrations in urine and blood/serum were similar, including in unadjusted or creatinine-adjusted measures (Colorado-Yohar *et al.*, 2021). Due to the less invasive nature of collection, urine is the most common sample collected to measure human BPA exposure, and this is reflected in the studies within this review.

The most prominent issue that remains for BPA detection in urine is that a single urine sample only reflects short-term exposure. The half-life of BPA in the human body is variably documented, however orally administered BPA can be rapidly absorbed, detected in the blood within 15 min, and excreted via urine within 24 h of exposure in a non-pregnant adult human (Thayer *et al.*, 2015). Despite its ability to be rapidly excreted, BPA also possesses moderately lipophilic properties and as such can accumulate in human tissues. It has been detected within a variety of human tissues, particularly adipose tissue, placenta, liver, and fetal tissue in addition to fluids such as breast milk and amniotic fluid (Corrales *et al.*, 2015). Yet regarding urine detection, the short-lived nature of BPA metabolism and excretion means single spot urine samples provide little insight into long-term exposure, particularly given that exposures are also often episodic and vary over time (Braun *et al.*, 2011). The human studies considered in this review commonly collected one or two urine samples per IVF cycle, detected BPA via isotope dilution high-performance liquid chromatography (HPLC) coupled with tandem mass spectrometry, and normalized urinary concentrations to creatinine or specific gravity (the ratio of the relative density of urine to water (Muscat *et al.*, 2011)) to correct for urine concentration. The geometric mean of urine concentrations was used if there was more than one sample per IVF treatment cycle, generating a single urinary BPA concentration per cycle. This BPA concentration was then statistically correlated with various outcomes of the IVF cycle in a cross-sectional or prospective context. This approach has the inherent limitation that BPA exposure may not be accurately represented owing to variations in exposure between days, time of day, and whether urine was collected during fasting (Ye *et al.*, 2008), all of which were factors that differed among these studies. Similar concerns regarding timing of urine sampling and using the geometric mean, obscuring episodic data from individual urine samples, were raised by the US FDA when reviewing the Mok-lin *et al.* (2010) study (FDA BPA Joint Emerging Science Working Group, 2011). In addition, indirect methods of sample preparation involving enzymatic deconjugation of the sample to hydrolyze conjugated BPA into free BPA prior to measurement via HPLC mass spectrometry have been demonstrated to underestimate BPA levels (Gerona *et al.*, 2020). To ensure the accuracy of BPA measurement in urine samples, direct quantification of BPA in its conjugated and unconjugated form is essential.

There were eight studies in this review that formed part of the EARTH prospective cohort study in Massachusetts, constituting almost half of the human clinical studies included. This raises concerns of bias within the findings of this scoping review due to overrepresentation of one demographic and the potential overlap of data and patients between these studies. With the exception of the EARTH cohort studies, studies that found adverse effects versus those that did not were conducted in different countries. However, studies comparing the BPA concentrations in populations across different continents including Asia, North America, and Europe, have demonstrated that BPA exposure is widespread and relatively comparable (median creatinine-adjusted concentration 1.36–2.41 µg/g) (Zhang *et al.*, 2011; Sarigiannis *et al.*, 2016; Colorado-Yohar *et al.*, 2021).

Despite literature documenting the impact of BPA alternatives on human health being much more scarce, emerging data from China, Sweden and Spain has revealed lower median urine concentrations (creatinine adjusted) of around 0.04–0.15 µg/g for BPF and 0.05–0.08 µg/g for BPS, compared to that of BPA (Derakhshan *et al.*, 2019; Sanchis *et al.*, 2020; Cui *et al.*, 2021). The one study measuring BPA alternatives reviewed herein tested follicular fluid rather than urine and documented average conjugated concentrations of 0.21 µg/l for BPS and 0.13 µg/l for BPF. These concentrations were higher than the average detected BPA concentration (0.075 µg/l); this is likely due to BPA being banned in food contact materials within France, however all bisphenols in this study had a low detection rate amongst participants (Lebachelier de la Riviere *et al.*, 2023).

BPA alternatives require much more characterization, and conclusions about their safety should be approached with caution as their toxicokinetics are not well understood. For example, in a sheep pregnancy model BPS was found to reach higher concentrations than BPA in the maternal circulation and have a longer half-life than BPA in the fetal compartment (Gingrich *et al.*, 2019). There is a pressing need for future studies in the fertility clinic setting to include a spectrum of bisphenols in their detection protocols to assess the prevalent BPA alternatives in the context of human fertility and oocyte health.

### Key recommendations for future studies

Key recommendations based on the findings from this review and elements of the US FDA Redbook 2000 (Toxicological Principles for the Safety Assessment of Food Ingredients) guidelines (U.S. Food and Drug Administration, 2000; Revised 2007) are provided in Table 2, for the purpose of informing best practice in future studies aimed at assessing the impact of bisphenol on oocyte health and female fertility. We contend that the adoption of these recommendations will help guide more consistent study outcomes, contributing to formulation of a robust and reliable evidence base. It is our hope that such information will provide an evidence-based framework to inform legislative bodies regarding the health implications of the spectrum of bisphenols we encounter, including those linked to low-dose exposures.

**Table 2.** Key recommendations for future studies assessing the impact of bisphenol exposure on oocyte health and female fertility.

Factor	Key recommendation
Concentration and dosing	*In vitro* and *in vivo* studies should employ at least three concentrations/doses or five if assessing non-monotonicity, including equivalent *in vitro* low doses below 50 ng/ml or *in vivo* doses below the NOAEL and/or TDI of BPA; the same concentrations/doses should be maintained for BPA alternatives. Animal dosing of bisphenols via oral delivery is preferable if assessing them within a dietary contaminant context, in line with US FDA requirements.
Employment of multiple bisphenols	*In vitro* and *in vivo*: Any BPA alternative such as BPS, BPF, BPB, BPAF etc. being assessed either in isolation or in combination with other bisphenols should be examined alongside BPA as an additional treatment group to characterize and compare the mechanisms of action. BPA can be used to provide a point of reference to build the profile of BPA analogues that are not yet well established within the literature; also generating additional evidence for BPA. Human studies in the fertility clinic setting should aim to incorporate the relevant alternative bisphenols (BPS, BPF, BPB, BPAF, etc.) in their detection protocols alongside BPA.
Endpoint measures	*In vitro* and *in vivo*: Studies prioritizing molecular and mechanistic endpoints should pair these with one or more comparable endpoints associated with oocyte or ovarian adverse effects (follicle counts, oocyte yield, oocyte meiotic capacity, oocyte and/or cumulus cell morphology, and oocyte meiotic spindle integrity). Human studies: Spindle assessment or morphological assessment of discarded oocytes from participants should be included where possible alongside current endpoints of oocyte yield and antral follicle count to link to animal study outcomes. ART outcomes including measures of embryo quality, embryo development, and implantation rates are also ideal to report in order to assess the developmental potential of oocytes selected for use in IVF/ICSI.
Methodological reporting	*In vitro* and *in vivo*: For study results to be considered by governing bodies, studies must include detailed reporting of methodologies including all aspects of animal use, experimental replicates, cell numbers, and dosing protocols as per US FDA guidelines. These guidelines such as the Redbook 2000 should be clearly defined, updated, and made accessible by the US FDA. Peer review processes for publication of these studies should hold authors to the correct reporting standards of methodologies in this toxicological context. Human studies should report the full range of statistical population parameters for BPA concentration in samples (means, standard deviation, quartiles/percentiles, maximums, the limit of detection, and percent of participants samples that were below the limit of detection, unadjusted and adjusted urinary parameters) for ease of interpretation and comparison between study population.
Sample collection and detection	Human observational studies should aim to collect multiple urine samples across different time points using a prospective cohort approach, consider these as separate data points, and where possible, measure unconjugated BPA. To obtain accurate results, it is critical that detection and quantification of BPA and BPA alternatives is conducted using the direct method (not indirect) as detailed in Gerona *et al.* 2020).

**Abbreviations:** BPA, Bisphenol A; BPAF, Bisphenol AF; BPB, Bisphenol B; BPF, Bisphenol F; BPS, Bisphenol S; NOAEL, no observed adverse effect level; TDI, tolerable daily intake; US FDA, United States Food and Drug Administration.

## Conclusion

Taken together, the available *in vitro* and *in vivo* evidence demonstrates that BPA has detrimental effects on oocyte health at low levels, that are well below the US FDA LOAEL and NOAEL for oral BPA exposure. This review has highlighted that BPA can impact follicle development, meiotic progression of the oocyte, the morphology of the oocyte or cumulus-oocyte-complex, as well as the integrity of the meiotic spindle, all of which are crucial components capable of contributing to poor oocyte quality and reduced fertility if disrupted. Concerningly, ‘BPA free’ alternatives that carry a public perception of safety can elicit the same spectrum of effects upon oocyte health. There is thus an urgent need for the revision of current guidelines by food safety authorities globally, following in the footsteps of the European FSA, to reduce prescribed safe exposure levels of BPA and introduce clear restrictions for the use of BPA analogues in line with the recommendations we have outlined. Ultimately, this scoping review highlights oocyte health as a fundamentally important endpoint in female reproductive toxicological studies, indicating an important direction for future research into endocrine disrupting chemicals to improve fertility outcomes.

## Data Availability

Key data generated for this article are available in the manuscript. Any other data generated from this article will be shared on reasonable request to the corresponding author.

## References

[dmae025-B1] Abdallah S , Jampy A, Moison D, Wieckowski M, Messiaen S, Martini E, Campalans A, Radicella JP, Rouiller-Fabre V, Livera G et al Foetal exposure to the bisphenols BADGE and BPAF impairs meiosis through DNA oxidation in mouse ovaries. Environ Pollut 2023;317:120791. 36464114 10.1016/j.envpol.2022.120791

[dmae025-B2] Acconcia F , Pallottini V, Marino M. Molecular mechanisms of action of BPA. Dose Response 2015;13:1559325815610582. 26740804 10.1177/1559325815610582 PMC4679188

[dmae025-B3] Acuña-Hernández DG , Arreola-Mendoza L, Santacruz-Márquez R, García-Zepeda SP, Parra-Forero LY, Olivares-Reyes JA, Hernández-Ochoa I. Bisphenol A alters oocyte maturation by prematurely closing gap junctions in the cumulus cell-oocyte complex. Toxicol Appl Pharmacol 2018;344:13–22. 29458137 10.1016/j.taap.2018.02.011

[dmae025-B4] Adegoke EO , Rahman MS, Pang M-G. Bisphenols threaten male reproductive health via testicular cells. Front Endocrinol (Lausanne) 2020;11:624. 33042007 10.3389/fendo.2020.00624 PMC7518410

[dmae025-B5] Adewale HB , Jefferson WN, Newbold RR, Patisaul HB. Neonatal Bisphenol-A exposure alters rat reproductive development and ovarian morphology without impairing activation of gonadotropin-releasing hormone neurons. Biol Reprod 2009;81:690–699. 19535786 10.1095/biolreprod.109.078261 PMC2754884

[dmae025-B6] Aftabsavad S , Noormohammadi Z, Moini A, Karimipoor M. Effect of Bisphenol A on alterations of ICAM-1 and HLA-G genes expression and DNA methylation profiles in cumulus cells of infertile women with poor response to ovarian stimulation. Sci Rep 2021;11:9595. 33953208 10.1038/s41598-021-87175-1 PMC8099902

[dmae025-B7] Almeida S , Raposo A, Almeida-González M, Carrascosa C. Bisphenol A: food exposure and impact on human health. Compr Rev Food Sci Food Saf 2018;17:1503–1517. 33350146 10.1111/1541-4337.12388

[dmae025-B8] Barnes K , Sinclair R, Watson D. Chemical Migration and Food Contact Materials. 1st edn. Cambridge, UK: Woodhead Publishing, 2006.

[dmae025-B9] Berger A , Ziv-Gal A, Cudiamat J, Wang W, Zhou C, Flaws JA. The effects of in utero Bisphenol A exposure on the ovaries in multiple generations of mice. Reprod Toxicol 2016;60:39–52. 26746108 10.1016/j.reprotox.2015.12.004 PMC4866900

[dmae025-B10] Bertoli S , Leone A, Battezzati A. Human Bisphenol A exposure and the “diabesity phenotype”. Dose-Response 2015;13:155932581559917. 10.1177/1559325815599173 PMC4734317 26858585

[dmae025-B11] Bloom MS , Kim D, Vom Saal FS, Taylor JA, Cheng G, Lamb JD, Fujimoto VY. Bisphenol A exposure reduces the estradiol response to gonadotropin stimulation during in vitro fertilization. Fertil Steril 2011;96:672–677.e672. 21813122 10.1016/j.fertnstert.2011.06.063 PMC3168558

[dmae025-B12] Bolon B , Bucci TJ, Warbritton AR, Chen JJ, Mattison DR, Heindel JJ. Differential follicle counts as a screen for chemically induced ovarian toxicity in mice: results from continuous breeding bioassays. Fundam Appl Toxicol 1997;39:1–10. 9325022 10.1006/faat.1997.2338

[dmae025-B13] Braun JM , Kalkbrenner AE, Calafat AM, Bernert JT, Ye X, Silva MJ, Barr DB, Sathyanarayana S, Lanphear BP. Variability and predictors of urinary Bisphenol A concentrations during pregnancy. Environ Health Perspect 2011;119:131–137. 21205581 10.1289/ehp.1002366 PMC3018492

[dmae025-B14] Brieño-Enríquez MA , Reig-Viader R, Cabero L, Toran N, Martínez F, Roig I, Garcia Caldés M. Gene expression is altered after Bisphenol A exposure in human fetal oocytes in vitro. Mol Hum Reprod 2012;18:171–183. 22121209 10.1093/molehr/gar074

[dmae025-B15] Brieño-Enríquez MA , Robles P, Camats-Tarruella N, García-Cruz R, Roig I, Cabero L, Martínez F, Caldés MG. Human meiotic progression and recombination are affected by Bisphenol A exposure during in vitro human oocyte development. Hum Reprod 2011;26:2807–2818. 21795248 10.1093/humrep/der249

[dmae025-B16] Calabrese EJ , Baldwin LA. U-Shaped dose-responses in biology, toxicology, and public health. Annu Rev Public Health 2001;22:15–33. 11274508 10.1146/annurev.publhealth.22.1.15

[dmae025-B17] Camacho L , Lewis SM, Vanlandingham MM, Olson GR, Davis KJ, Patton RE, Twaddle NC, Doerge DR, Churchwell MI, Bryant MS et al A two-year toxicology study of bisphenol A (BPA) in Sprague-Dawley rats: CLARITY-BPA core study results. Food Chem Toxicol 2019;132:110728. 31365888 10.1016/j.fct.2019.110728

[dmae025-B18] Campen KA , Kucharczyk KM, Bogin B, Ehrlich JM, Combelles CMH. Spindle abnormalities and chromosome misalignment in bovine oocytes after exposure to low doses of Bisphenol A or Bisphenol S. Hum Reprod 2018;33:895–904. 29538760 10.1093/humrep/dey050 PMC5925783

[dmae025-B3693585] Can A, , Semiz O, , Cinar O. Bisphenol-A induces cell cycle delay and alters centrosome and spindle microtubular organization in oocytes during meiosis. Mol Hum Reprod 2005;11:389–396. 15879462 10.1093/molehr/gah179

[dmae025-B19] Campen KA , McNatty KP, Pitman JL. A protective role of cumulus cells after short-term exposure of rat cumulus cell-oocyte complexes to lifestyle or environmental contaminants. Reprod Toxicol 2017;69:19–33. 28087314 10.1016/j.reprotox.2017.01.003

[dmae025-B20] Cariati F , Carbone L, Conforti A, Bagnulo F, Peluso SR, Carotenuto C, Buonfantino C, Alviggi E, Alviggi C, Strina I. Bisphenol A-induced epigenetic changes and its effects on the male reproductive system. Front Endocrinol (Lausanne) 2020;11:453. 32849263 10.3389/fendo.2020.00453 PMC7406566

[dmae025-B21] Chao H-H , Zhang X-F, Chen B, Pan B, Zhang L-J, Li L, Sun X-F, Shi Q-H, Shen W. Bisphenol A exposure modifies methylation of imprinted genes in mouse oocytes via the estrogen receptor signaling pathway. Histochem Cell Biol 2012;137:249–259. 22131059 10.1007/s00418-011-0894-z

[dmae025-B22] Chavarro JE , Minguez-Alarcon L, Chiu YH, Gaskins AJ, Souter I, Williams PL, Calafat AM, Hauser R. and Team ES. Soy intake modifies the relation between urinary Bisphenol A concentrations and pregnancy outcomes among women undergoing assisted reproduction. J Clin Endocrinol Metab 2016;101:1082–1090. 26815879 10.1210/jc.2015-3473 PMC4803173

[dmae025-B23] Colorado-Yohar SM , Castillo-González AC, Sánchez-Meca J, Rubio-Aparicio M, Sánchez-Rodríguez D, Salamanca-Fernández E, Ardanaz E, Amiano P, Fernández MF, Mendiola J et al Concentrations of Bisphenol-A in adults from the general population: A systematic review and meta-analysis. Sci Total Environ 2021;775:145755. 34132197 10.1016/j.scitotenv.2021.145755

[dmae025-B24] Corrales J , Kristofco LA, Steele WB, Yates BS, Breed CS, Williams ES, Brooks BW. Global Assessment of Bisphenol A in the environment: review and analysis of its occurrence and bioaccumulation. Dose Response 2015;13:1559325815598308. 26674671 10.1177/1559325815598308 PMC4674187

[dmae025-B25] Cui F-P , Yang P, Liu C, Chen P-P, Deng Y-L, Miao Y, Luo Q, Zhang M, Lu W-Q, Zeng Q. Urinary Bisphenol A and its alternatives among pregnant women: predictors and risk assessment. Sci Total Environ 2021;784:147184. 33901963 10.1016/j.scitotenv.2021.147184

[dmae025-B26] Derakhshan A , Shu H, Peeters RP, Kortenkamp A, Lindh CH, Demeneix B, Bornehag C-G, Korevaar TIM. Association of urinary bisphenols and triclosan with thyroid function during early pregnancy. Environ Int 2019;133:105123. 31521814 10.1016/j.envint.2019.105123

[dmae025-B66545827] Desmarchais A, , Téteau O, , Papillier P, , Jaubert M, , Druart X, , Binet A, , Maillard V, , Elis S. Bisphenol S impaired in vitro ovine early developmental oocyte competence. Int J Mol Sci 2020;21:1238. 32059612 10.3390/ijms21041238 PMC7072985

[dmae025-B27] Desmarchais A , Teteau O, Kasal-Hoc N, Cognie J, Lasserre O, Papillier P, Lacroix M, Vignault C, Jarrier-Gaillard P, Maillard V et al Chronic low BPS exposure through diet impairs in vitro embryo production parameters according to metabolic status in the ewe. Ecotoxicol Environ Saf 2022;229:113096. 34952380 10.1016/j.ecoenv.2021.113096

[dmae025-B28] Ding ZM , Chen YW, Ahmad MJ, Wang YS, Yang SJ, Duan ZQ, Liu M, Yang CX, Liang AX, Hua GH et al Bisphenol F exposure affects mouse oocyte in vitro maturation through inducing oxidative stress and DNA damage. Environ Toxicol 2022;37:1413–1422. 35218298 10.1002/tox.23494

[dmae025-B29] Ding ZM , Jiao XF, Wu D, Zhang JY, Chen F, Wang YS, Huang CJ, Zhang SX, Li X, Huo LJ. Bisphenol AF negatively affects oocyte maturation of mouse in vitro through increasing oxidative stress and DNA damage. Chem Biol Interact 2017;278:222–229. 29102535 10.1016/j.cbi.2017.10.030

[dmae025-B30] Durcik M , Hiti L, Tomašič T, Mašič LP. New Bisphenol A and Bisphenol S analogs: evaluation of their hERα agonistic and antagonistic activities using the OECD 455 in-vitro assay and molecular modeling. Chem Biol Interact 2022;354:109820. 35077665 10.1016/j.cbi.2022.109820

[dmae025-B31] Ehrlich S , Williams PL, Missmer SA, Flaws JA, Ye X, Calafat AM, Petrozza JC, Wright D, Hauser R. Urinary bisphenol A concentrations and early reproductive health outcomes among women undergoing IVF. Hum Reprod 2012;27:3583–3592. 23014629 10.1093/humrep/des328 PMC3501244

[dmae025-B32] Eichenlaub-Ritter U , Vogt E, Cukurcam S, Sun F, Pacchierotti F, Parry J. Exposure of mouse oocytes to bisphenol A causes meiotic arrest but not aneuploidy. Mutat Res 2008;651:82–92. 18096426 10.1016/j.mrgentox.2007.10.014

[dmae025-B33] Ejaredar M , Lee Y, Roberts DJ, Sauve R, Dewey D. Bisphenol A exposure and children’s behavior: A systematic review. J Expo Sci Environ Epidemiol 2017;27:175–183. 26956939 10.1038/jes.2016.8

[dmae025-B34] Eladak S , Grisin T, Moison D, Guerquin M-J, N'Tumba-Byn T, Pozzi-Gaudin S, Benachi A, Livera G, Rouiller-Fabre V, Habert R. A new chapter in the bisphenol A story: bisphenol S and bisphenol F are not safe alternatives to this compound. Fertil Steril 2015;103:11–21. 25475787 10.1016/j.fertnstert.2014.11.005

[dmae025-B35] EPA. Guidelines for reproductive toxicity risk assessment [FRL-5630-6]. Federal Register 61(212):56274-56322. Washington, DC. 1996.

[dmae025-B36] EPA. Bisphenol A alternatives in thermal paper, final report. Washington, DC. 2014. https://www.epa.gov/sites/default/files/2015-08/documents/bpa_final.pdf (20 May 2024, date last accessed).

[dmae025-B37] European Commission. Commission Directive 2011/8/EU of 28 January 2011 amending Directive 2002/72/EC as regards the restriction of use of Bisphenol A in plastic infant feeding bottles. 2011.

[dmae025-B38] FDA BPA Joint Emerging Science Working Group. Updated review of the ‘low-dose’ literature (data) on Bisphenol A (CAS RN 80-05-7) and response to charge questions regarding the risk assessment on Bisphenol A. 2011.

[dmae025-B39] FDA BPA Joint Emerging Science Working Group. 2012 updated review of literature and data on Bisphenol A (CAS RN 80-05-7). 2013.

[dmae025-B40] FDA BPA Joint Emerging Science Working Group. 2014 updated review of literature and data on Bisphenol A (CAS RN 80-05-7). 2014a.

[dmae025-B41] FDA BPA Joint Emerging Science Working Group. 2014 updated safety assessment of Bisphenol A (BPA) for use in food contact applications. 2014b.

[dmae025-B42] Fernández M , Bourguignon N, Lux-Lantos V, Libertun C. Neonatal exposure to Bisphenol A and reproductive and endocrine alterations resembling the polycystic ovarian syndrome in adult rats. Environ Health Perspect 2010;118:1217–1222. 20413367 10.1289/ehp.0901257 PMC2944080

[dmae025-B43] Ferris J , Favetta LA, King WA. Bisphenol A exposure during oocyte maturation in vitro results in spindle abnormalities and chromosome misalignment in *Bos taurus*. Cytogenet Genome Res 2015;145:50–58. 25871885 10.1159/000381321

[dmae025-B44] Ferris J , Mahboubi K, MacLusky N, King WA, Favetta LA. BPA exposure during in vitro oocyte maturation results in dose-dependent alterations to embryo development rates, apoptosis rate, sex ratio and gene expression. Reprod Toxicol 2016;59:128–138. 26686065 10.1016/j.reprotox.2015.12.002

[dmae025-B45] FitzGerald R , Loveren HV, Civitella C, Castoldi AF, Bernasconi G, European Food Safety Authority (EFSA). Assessment of new information on Bisphenol S (BPS) submitted in response to the Decision 1 under REACH Regulation (EC) No 1907/2006. EFSA Support Publ 2020;17:1844E.

[dmae025-B46] Food and Drug Administration. Indirect food additives: polymers 77 FR 41899. 2012.

[dmae025-B47] Food Standards Australia New Zealand. FSANZ activities in relation to Bisphenol A. 2010.

[dmae025-B48] Fujimoto VY , Kim D, Vom Saal FS, Lamb JD, Taylor JA, Bloom MS. Serum unconjugated bisphenol A concentrations in women may adversely influence oocyte quality during in vitro fertilization. Fertil Steril 2011;95:1816–1819. 21122836 10.1016/j.fertnstert.2010.11.008

[dmae025-B49] Futran Fuhrman V , Tal A, Arnon S. Why endocrine disrupting chemicals (EDCs) challenge traditional risk assessment and how to respond. J Hazard Mater 2015;286:589–611. 25646754 10.1016/j.jhazmat.2014.12.012

[dmae025-B50] Ganesan S , Keating AF. Bisphenol A-induced ovotoxicity involves DNA damage induction to which the ovary mounts a protective response indicated by increased expression of proteins involved in DNA repair and xenobiotic biotransformation. Toxicol Sci 2016;152:169–180. 27208089 10.1093/toxsci/kfw076

[dmae025-B51] Génard-Walton M , McGee G, Williams PL, Souter I, Ford JB, Chavarro JE, Calafat AM, Hauser R, Mínguez-Alarcón L. Mixtures of urinary concentrations of phenols and phthalate biomarkers in relation to the ovarian reserve among women attending a fertility clinic. Sci Total Environ 2023;898:165536. 37453702 10.1016/j.scitotenv.2023.165536

[dmae025-B52] Gerona R , Vom Saal FS, Hunt PA. BPA: have flawed analytical techniques compromised risk assessments? Lancet Diabetes Endocrinol 2020;8:11–13. 31813841 10.1016/S2213-8587(19)30381-X

[dmae025-B53] Gingrich J , Pu Y, Ehrhardt R, Karthikraj R, Kannan K, Veiga-Lopez A. Toxicokinetics of Bisphenol A, Bisphenol S, and Bisphenol F in a pregnancy sheep model. Chemosphere 2019;220:185–194. 30583211 10.1016/j.chemosphere.2018.12.109 PMC6363860

[dmae025-B54] Ginsberg G , Rice DC. Does rapid metabolism ensure negligible risk from Bisphenol A? Environ Health Perspect 2009;117:1639–1643. 20049111 10.1289/ehp.0901010 PMC2801165

[dmae025-B55] González-Gómez M , Reyes R, Damas-Hernández MDC, Plasencia-Cruz X, González-Marrero I, Alonso R, Bello AR. NTS, NTSR1 and ERs in the pituitary–gonadal axis of cycling and postnatal female rats after BPA treatment. Int J Mol Sci 2023;24:7418. 37108581 10.3390/ijms24087418 PMC10138486

[dmae025-B56] Hamid N , Junaid M, Pei D-S. Combined toxicity of endocrine-disrupting chemicals: a review. Ecotoxicol Environ Saf 2021;215:112136. 33735605 10.1016/j.ecoenv.2021.112136

[dmae025-B57] Han J-W , Ruiz-Garcia L, Qian J-P, Yang X-T. Food packaging: a comprehensive review and future trends. Compr Rev Food Sci Food Saf 2018;17:860–877. 33350114 10.1111/1541-4337.12343

[dmae025-B58] Herez SH , Jawad MA, Ms A. Effect of Bisphenol A level in follicular fluid on ICSI OutCome. J Pharm Negative Results 2022;13:370–376.

[dmae025-B59] Hu Y , Yuan DZ, Wu Y, Yu LL, Xu LZ, Yue LM, Liu L, Xu WM, Qiao XY, Zeng RJ et al Bisphenol A initiates excessive premature activation of primordial follicles in mouse ovaries via the PTEN signaling pathway. Reprod Sci 2018;25:609–620. 28982275 10.1177/1933719117734700

[dmae025-B60] Huang R , Li J, Liao M, Ma L, Laurent I, Lin X, Zhang Y, Gao R, Ding Y, Xiao X. Combinational exposure to Bisphenol A and a high-fat diet causes trans-generational malfunction of the female reproductive system in mice. Mol Cell Endocrinol 2022;541:111507. 34785282 10.1016/j.mce.2021.111507

[dmae025-B61] Huang R-P , Liu Z-H, Yin H, Dang Z, Wu P-X, Zhu N-W, Lin Z. Bisphenol A concentrations in human urine, human intakes across six continents, and annual trends of average intakes in adult and child populations worldwide: A thorough literature review. Sci Total Environ 2018;626:971–981. 29898562 10.1016/j.scitotenv.2018.01.144

[dmae025-B62] Hunt PA , Koehler KE, Susiarjo M, Hodges CA, Ilagan A, Voigt RC, Thomas S, Thomas BF, Hassold TJ. Bisphenol A exposure causes meiotic aneuploidy in the female mouse. Curr Biol 2003;13:546–553. 12676084 10.1016/s0960-9822(03)00189-1

[dmae025-B63] Hunt PA , Lawson C, Gieske M, Murdoch B, Smith H, Marre A, Hassold T, VandeVoort CA. Bisphenol A alters early oogenesis and follicle formation in the fetal ovary of the rhesus monkey. Proc Natl Acad Sci USA 2012;109:17525–17530. 23012422 10.1073/pnas.1207854109 PMC3491481

[dmae025-B64] Inoue A , Nakajima R, Nagata M, Aoki F. Contribution of the oocyte nucleus and cytoplasm to the determination of meiotic and developmental competence in mice. Hum Reprod 2008;23:1377–1384. 18367455 10.1093/humrep/den096

[dmae025-B65] Iqbal K , Tran DA, Li AX, Warden C, Bai AY, Singh P, Wu X, Pfeifer GP, Szabo PE. Deleterious effects of endocrine disruptors are corrected in the mammalian germline by epigenome reprogramming. Genome Biol 2015;16:59. 25853433 10.1186/s13059-015-0619-z PMC4376074

[dmae025-B66] Jia Z , Wang H, Feng Z, Zhang S, Wang L, Zhang J, Liu Q, Zhao X, Feng D, Feng X. Fluorene-9-bisphenol exposure induces cytotoxicity in mouse oocytes and causes ovarian damage. Ecotoxicol Environ Saf 2019;180:168–178. 31082581 10.1016/j.ecoenv.2019.05.019

[dmae025-B67] Jiao X , Ding Z, Meng F, Zhang X, Wang Y, Chen F, Duan Z, Wu D, Zhang S, Miao Y et al The toxic effects of Fluorene-9-bisphenol on porcine oocyte in vitro maturation. Environ Toxicol 2020;35:152–158. 31696613 10.1002/tox.22851

[dmae025-B68] Jiao XF , Liang QM, Wu D, Ding ZM, Zhang JY, Chen F, Wang YS, Zhang SX, Miao YL, Huo LJ. Effects of acute Fluorene-9-bisphenol exposure on mouse oocyte in vitro maturation and its possible mechanisms. Environ Mol Mutagen 2019;60:243–253. 30499614 10.1002/em.22258

[dmae025-B69] Jones RL , Lang SA, Kendziorski JA, Greene AD, Burns KA. Use of a mouse model of experimentally induced endometriosis to evaluate and compare the effects of Bisphenol A and Bisphenol AF exposure. Environ Health Perspect 2018;126:127004. 30675821 10.1289/EHP3802 PMC6371646

[dmae025-B70] Karavan JR , Pepling ME. Effects of estrogenic compounds on neonatal oocyte development. Reprod Toxicol 2012;34:51–56. 22406039 10.1016/j.reprotox.2012.02.005

[dmae025-B71] Karrer C , Roiss T, von Goetz N, Gramec Skledar D, Peterlin Mašič L, Hungerbühler K. Physiologically based pharmacokinetic (PBPK) modeling of the bisphenols BPA, BPS, BPF, and BPAF with new experimental metabolic parameters: comparing the pharmacokinetic behavior of BPA with its substitutes. Environ Health Perspect 2018;126:077002. 29995627 10.1289/EHP2739 PMC6108829

[dmae025-B72] Khmiri I , Côté J, Mantha M, Khemiri R, Lacroix M, Gely C, Toutain P-L, Picard-Hagen N, Gayrard V, Bouchard M. Toxicokinetics of Bisphenol-S and its glucuronide in plasma and urine following oral and dermal exposure in volunteers for the interpretation of biomonitoring data. Environ Int 2020;138:105644. 32179324 10.1016/j.envint.2020.105644

[dmae025-B73] Kim HK , Ko DH, Lee W, Kim KR, Chun S, Song J, Min WK. Body fluid concentrations of Bisphenol A and their association with in vitro fertilization outcomes. Hum Fertil (Camb) 2021;24:199–207. 31099279 10.1080/14647273.2019.1612104

[dmae025-B74] Kojima H , Takeuchi S, Sanoh S, Okuda K, Kitamura S, Uramaru N, Sugihara K, Yoshinari K. Profiling of Bisphenol A and eight of its analogues on transcriptional activity via human nuclear receptors. Toxicology 2019;413:48–55. 30582956 10.1016/j.tox.2018.12.001

[dmae025-B75] Krisher RL. In vivo and in vitro environmental effects on mammalian oocyte quality. Annu Rev Anim Biosci 2013;1:393–417. 25387025 10.1146/annurev-animal-031412-103647

[dmae025-B76] Lambré C , Barat Baviera JM, Bolognesi C, Chesson A, Cocconcelli PS, Crebelli R, Gott DM, Grob K, Lampi E, Mengelers M et al; EFSA Panel on Food Contact Materials E. Re-evaluation of the risks to public health related to the presence of bisphenol A (BPA) in foodstuffs. EFSA J 2023;21:e06857. 37089179 10.2903/j.efsa.2023.6857 PMC10113887

[dmae025-B77] Lebachelier de la Riviere ME , Wu L, Gayet M, Bousquet M, Buron C, Vignault C, Teteau O, Desmarchais A, Maillard V, Uzbekova S et al Cumulative and potential synergistic effects of seven different bisphenols on human granulosa cells in vitro? Environ Pollut 2023;330:121818. 37182577 10.1016/j.envpol.2023.121818

[dmae025-B78] Lenie S , Cortvrindt R, Eichenlaub-Ritter U, Smitz J. Continuous exposure to bisphenol A during in vitro follicular development induces meiotic abnormalities. Mutat Res 2008;651:71–81. 18093867 10.1016/j.mrgentox.2007.10.017

[dmae025-B79] Li C , Qi T, Ma L, Lan YB, Luo J, Chu K, Huang Y, Ruan F, Zhou J. In utero Bisphenol A exposure disturbs germ cell cyst breakdown through the PI3k/Akt signaling pathway and BDNF expression. Ecotoxicol Environ Saf2023a;259:115031. 37210998 10.1016/j.ecoenv.2023.115031

[dmae025-B80] Li Q , Zhao Z. Influence of N-acetyl-L-cysteine against bisphenol a on the maturation of mouse oocytes and embryo development: in vitro study. BMC Pharmacol Toxicol 2019;20:43. 31331389 10.1186/s40360-019-0323-9 PMC6647297

[dmae025-B81] Li X , Wen Z, Wang Y, Mo J, Zhong Y, Ge R-S. Bisphenols and leydig cell development and function. Front Endocrinol (Lausanne) 2020;11:447. 32849262 10.3389/fendo.2020.00447 PMC7411000

[dmae025-B82] Li Y , Liu S, Gao F, Peng Z, Zhang J, Li S, Lu D, Pan X. BPA interferes with granulosa cell development and oocyte meiosis in mouse preantral follicles. Exp Biol Med (Maywood) 2023b;248:1145–1158. 37452689 10.1177/15353702231179940 PMC10583751

[dmae025-B83] Li Y , Zhang W, Liu J, Wang W, Li H, Zhu J, Weng S, Xiao S, Wu T. Prepubertal Bisphenol A exposure interferes with ovarian follicle development and its relevant gene expression. Reprod Toxicol 2014;44:33–40. 24051130 10.1016/j.reprotox.2013.09.002

[dmae025-B84] Li YQ , Dong Z, Liu ST, Gao F, Zhang JY, Peng ZD, Wang LX, Pan XY. Astaxanthin improves the development of the follicles and oocytes through alleviating oxidative stress induced by BPA in cultured follicles. Sci Rep 2022;12:7853. 35551214 10.1038/s41598-022-11566-1 PMC9098901

[dmae025-B85] Lin ML , Hua R, Ma J, Zhou Y, Li P, Xu XY, Yu ZQ, Quan S. Bisphenol A promotes autophagy in ovarian granulosa cells by inducing AMPK/mTOR/ULK1 signalling pathway. Environ Int 2021;147:106298. 33387880 10.1016/j.envint.2020.106298

[dmae025-B86] Liu B , Zhou S, Yang C, Chen P, Chen P, Xi D, Zhu H, Gao Y. Bisphenol A deteriorates egg quality through HDAC7 suppression. Oncotarget 2017;8:92359–92365. 29190921 10.18632/oncotarget.21308 PMC5696187

[dmae025-B87] Liu WX , Donatella F, Tan SJ, Ge W, Wang JJ, Sun XF, Cheng SF, Shen W. Detrimental effect of Bisphenol S in mouse germ cell cyst breakdown and primordial follicle assembly. Chemosphere 2021;264:128445. 33017704 10.1016/j.chemosphere.2020.128445

[dmae025-B88] Liu X , Shi H, Xie B, Dionysiou DD, Zhao Y. Microplastics as both a sink and a source of Bisphenol A in the marine environment. Environ Sci Technol 2019;53:10188–10196. 31393116 10.1021/acs.est.9b02834

[dmae025-B89] López-Cervantes J , Paseiro-Losada P. Determination of Bisphenol A in, and its migration from, PVC stretch film used for food packaging. Food Addit Contam 2003;20:596–606. 12881134 10.1080/0265203031000109495

[dmae025-B90] Loup B , Poumerol E, Jouneau L, Fowler PA, Cotinot C, Mandon-Pepin B. BPA disrupts meiosis I in oogonia by acting on pathways including cell cycle regulation, meiosis initiation and spindle assembly. Reprod Toxicol 2022;111:166–177. 35667523 10.1016/j.reprotox.2022.06.001

[dmae025-B91] Machtinger R , Combelles CMH, Missmer SA, Correia KF, Williams P, Hauser R, Racowsky C. Bisphenol-A and human oocyte maturation in vitro. Hum Reprod 2013;28:2735–2745. 23904465 10.1093/humrep/det312 PMC3777571

[dmae025-B92] Makowska K , Staniszewska M, Bodziach K, Calka J, Gonkowski S. Concentrations of Bisphenol A (BPA) in fresh pork loin meat under standard stock-farming conditions and after oral exposure—A preliminary study. Chemosphere 2022;295:133816. 35131273 10.1016/j.chemosphere.2022.133816

[dmae025-B93] Mansoori T , Soleimani Mehranjani M, Shariatzadeh MA, Mahmoodi M, Noreini N. S. The protective effect of vitamin C on adverse effect of bisphenol a on the ovary in adult rat. Int J Fertil Steril 2013;7:101.

[dmae025-B94] Mehranjani MS , Mansoori T. Stereological study on the effect of vitamin C in preventing the adverse effects of bisphenol A on rat ovary. IJRM 2016;14:403–410. 27525324 PMC4971555

[dmae025-B95] Metz CM. Bisphenol A: understanding the controversy. Workplace Health Saf 2016;64:28–36; quiz 37. 26800896 10.1177/2165079915623790

[dmae025-B96] Mina A , Boutzios G, Papoutsis I, Kaparos G, Christopoulos P, Kousta E, Mastrominas M, Athanaselis S, Mastorakos G. Bisphenol A correlates with fewer retrieved oocytes in women with tubal factor infertility. Hormones (Athens) 2022;21:305–315. 35524040 10.1007/s42000-022-00370-1

[dmae025-B97] Mínguez-Alarcón L , Gaskins AJ, Chiu YH, Souter I, Williams PL, Calafat AM, Hauser R, Chavarro JE, TES. EARTH Study Team. Dietary folate intake and modification of the association of urinary Bisphenol A concentrations with in vitro fertilization outcomes among women from a fertility clinic. Reprod Toxicol 2016;65:104–112. 27423903 10.1016/j.reprotox.2016.07.012 PMC5067190

[dmae025-B98] Mínguez-Alarcón L , Gaskins AJ, Chiu YH, Williams PL, Ehrlich S, Chavarro JE, Petrozza JC, Ford JB, Calafat AM, Hauser R; EARTH Study Team. Urinary Bisphenol A concentrations and association with in vitro fertilization outcomes among women from a fertility clinic. Hum Reprod 2015;30:2120–2128. 26209788 10.1093/humrep/dev183 PMC4542722

[dmae025-B99] Mínguez-Alarcón L , Messerlian C, Bellavia A, Gaskins AJ, Chiu YH, Ford JB, Azevedo AR, Petrozza JC, Calafat AM, Hauser R, Earth Study Team et al Urinary concentrations of bisphenol A, parabens and phthalate metabolite mixtures in relation to reproductive success among women undergoing in vitro fertilization. Environ Int 2019;126:355–362. 30826614 10.1016/j.envint.2019.02.025 PMC6469504

[dmae025-B2486654] Mlynarcíková A, , Nagyová EVA, , Ficková M, , Scsuková S. Effects of selected endocrine disruptors on meiotic maturation, cumulus expansion, synthesis of hyaluronan and progesterone by porcine oocyte-cumulus complexes. Toxicol in Vitro 2009;23:371–377. 19162163 10.1016/j.tiv.2008.12.017

[dmae025-B100] Mohri T , Yoshida S. Estrogen and Bisphenol A disrupt spontaneous [Ca(2+)](i) oscillations in mouse oocytes. Biochem Biophys Res Commun 2005;326:166–173. 15567167 10.1016/j.bbrc.2004.11.024

[dmae025-B101] Mok-Lin E , Ehrlich S, Williams PL, Petrozza J, Wright DL, Calafat AM, Ye X, Hauser R. Urinary Bisphenol A concentrations and ovarian response among women undergoing IVF. Int J Androl 2010;33:385–393. 20002217 10.1111/j.1365-2605.2009.01014.x PMC3089904

[dmae025-B102] Moon MK. Concern about the safety of Bisphenol A substitutes. Diabetes Metab J 2019;43:46–48. 30793551 10.4093/dmj.2019.0027 PMC6387873

[dmae025-B103] Moore-Ambriz TR , Acuna-Hernandez DG, Ramos-Robles B, Sanchez-Gutierrez M, Santacruz-Marquez R, Sierra-Santoyo A, Pina-Guzman B, Shibayama M, Hernandez-Ochoa I. Exposure to bisphenol A in young adult mice does not alter ovulation but does alter the fertilization ability of oocytes. Toxicol Appl Pharmacol 2015;289:507–514. 26493930 10.1016/j.taap.2015.10.010

[dmae025-B104] Muhlhauser A , Susiarjo M, Rubio C, Griswold J, Gorence G, Hassold T, Hunt PA. Bisphenol A effects on the growing mouse oocyte are influenced by diet. Biol Reprod 2009;80:1066–1071. 19164168 10.1095/biolreprod.108.074815 PMC2804836

[dmae025-B105] Muncke J , Myers JP, Scheringer M, Porta M. Food packaging and migration of food contact materials: will epidemiologists rise to the neotoxic challenge? J Epidemiol Community Health 2014;68:592–594. 24554760 10.1136/jech-2013-202593

[dmae025-B106] Munn Z , Peters MDJ, Stern C, Tufanaru C, McArthur A, Aromataris E. Systematic review or scoping review? Guidance for authors when choosing between a systematic or scoping review approach. BMC Med Res Methodol 2018;18:143. 30453902 10.1186/s12874-018-0611-x PMC6245623

[dmae025-B107] Muscat JE , Liu A, Richie JP. Jr., A comparison of creatinine vs. specific gravity to correct for urinary dilution of cotinine. Biomarkers 2011;16:206–211. 21288164 10.3109/1354750X.2010.538084 PMC3631104

[dmae025-B108] Nakano K , Nishio M, Kobayashi N, Hiradate Y, Hoshino Y, Sato E, Tanemura K. Comparison of the effects of BPA and BPAF on oocyte spindle assembly and polar body release in mice. Zygote 2016;24:172–180. 25925194 10.1017/S0967199415000027

[dmae025-B109] Nevoral J , Havrankova J, Kolinko Y, Prokesova S, Fenclova T, Monsef L, Zalmanova T, Petr J, Kralickova M. Exposure to alternative bisphenols BPS and BPF through breast milk: noxious heritage effect during nursing associated with idiopathic infertility. Toxicol Appl Pharmacol 2021;413:115409. 33476676 10.1016/j.taap.2021.115409

[dmae025-B110] Nevoral J , Kolinko Y, Moravec J, Žalmanová T, Hošková K, Prokešová Š, Klein P, Ghaibour K, Hošek P, Štiavnická M et al Long-term exposure to very low doses of Bisphenol S affects female reproduction. Reproduction 2018;156:47–57. 29748175 10.1530/REP-18-0092

[dmae025-B111] Nguyen M , Sabry R, Davis OS, Favetta LA. Effects of BPA, BPS, and BPF on oxidative stress and antioxidant enzyme expression in bovine oocytes and spermatozoa. Genes (Basel) 2022;13:142. 35052481 10.3390/genes13010142 PMC8774721

[dmae025-B112] NICNAS. Phenol, 4,4′-sulfonylbis-: human health tier II assessment. IMAP Single Assessment Report. 2015.

[dmae025-B113] NICNAS. Bisphenol S (BPS)-based polymers: human health tier II assessment. IMAP Group Assessment Report. 2019.

[dmae025-B114] Nilsson E , Larsen G, Manikkam M, Guerrero-Bosagna C, Savenkova MI, Skinner MK. Environmentally induced epigenetic transgenerational inheritance of ovarian disease. PLoS One 2012;7:e36129. 22570695 10.1371/journal.pone.0036129 PMC3343040

[dmae025-B115] Nourian A , Soleimanzadeh A, Jalali AS, Najafi G. Effects of bisphenol-S low concentrations on oxidative stress status and in vitro fertilization potential in mature female mice. Vet Res Forum 2017;8:341–345. 29326794 PMC5756255

[dmae025-B9680633] Ozkemahli G, , Balci Ozyurt A, , Erkekoglu P, , Zeybek ND, , Yersal N, , Kocer-Gumusel B. The effects of prenatal and lactational bisphenol A and/or di(2-ethylhexyl) phthalate exposure on female reproductive system. Toxicol Mech Methods 2022;32:597–605. 35321620 10.1080/15376516.2022.2057265

[dmae025-B116] Pacchierotti F , Ranaldi R, Eichenlaub-Ritter U, Attia S, Adler ID. Evaluation of aneugenic effects of Bisphenol A in somatic and germ cells of the mouse. Mutat Res 2008;651:64–70. 18083607 10.1016/j.mrgentox.2007.10.009

[dmae025-B117] Pal S , Sahu A, Verma R, Haldar C. BPS-induced ovarian dysfunction: protective actions of melatonin via modulation of SIRT-1/Nrf2/NFkB and IR/PI3K/pAkt/GLUT-4 expressions in adult golden hamster. J Pineal Res 2023;75:e12869. 37002642 10.1111/jpi.12869

[dmae025-B118] Pan J , Liu P, Yu X, Zhang Z, Liu J. The adverse role of endocrine disrupting chemicals in the reproductive system. Front Endocrinol (Lausanne) 2024;14:1324993. 38303976 10.3389/fendo.2023.1324993 PMC10832042

[dmae025-B119] Pan M-H , Wu Y-K, Liao B-Y, Zhang H, Li C, Wang J-L, Hu L-L, Ma B. Bisphenol A exposure disrupts organelle distribution and functions during mouse oocyte maturation. Front Cell Dev Biol 2021;9:661155. 33834027 10.3389/fcell.2021.661155 PMC8021768

[dmae025-B120] Park HJ , Park SY, Kim JW, Yang SG, Kim MJ, Jegal HG, Kim IS, Choo YK, Koo DB. Melatonin improves oocyte maturation and mitochondrial functions by reducing bisphenol a-derived superoxide in porcine oocytes in vitro. Int J Mol Sci 2018;19:3422. 30384504 10.3390/ijms19113422 PMC6274783

[dmae025-B121] Pelch K , Wignall JA, Goldstone AE, Ross PK, Blain RB, Shapiro AJ, Holmgren SD, Hsieh J-H, Svoboda D, Auerbach SS et al A scoping review of the health and toxicological activity of bisphenol A (BPA) structural analogues and functional alternatives. Toxicology 2019;424:152235. 31201879 10.1016/j.tox.2019.06.006

[dmae025-B122] Peters AE , Mihalas BP, Bromfield EG, Roman SD, Nixon B, Sutherland JM. Autophagy in female fertility: a role in oxidative stress and aging. Antioxid Redox Signal 2020;32:550–568. 31892284 10.1089/ars.2019.7986

[dmae025-B123] Poormoosavi SM , Behmanesh MA, Janati S, Najafzadehvarzi H. Level of Bisphenol A in follicular fluid and serum and oocyte morphology in patients undergoing IVF treatment. J Family Reprod Health 2019;13:154–159. 32201490 PMC7072031

[dmae025-B1530005] Prabhu NB, , Adiga D, , Kabekkodu SP, , Bhat SK, , Satyamoorthy K, , Rai PS. Bisphenol A exposure modulates reproductive and endocrine system, mitochondrial function and cellular senescence in female adult rats: A hallmarks of polycystic ovarian syndrome phenotype. Environ Toxicol Pharmacol 2022;96:104010. 36334871 10.1016/j.etap.2022.104010

[dmae025-B124] Prokešová Š , Ghaibour K, Liška F, Klein P, Fenclová T, Štiavnická M, Hošek P, Žalmanová T, Hošková K, Řimnáčová H et al Acute low-dose bisphenol S exposure affects mouse oocyte quality. Reprod Toxicol 2020;93:19–27. 31881267 10.1016/j.reprotox.2019.12.005

[dmae025-B125] Radwan P , Wielgomas B, Radwan M, Krasiński R, Klimowska A, Kaleta D, Jurewicz J. Urinary Bisphenol A concentrations and in vitro fertilization outcomes among women from a fertility clinic. Reprod Toxicol 2020;96:216–220. 32721521 10.1016/j.reprotox.2020.07.009

[dmae025-B126] Rancière F , Lyons JG, Loh VH, Botton J, Galloway T, Wang T, Shaw JE, Magliano DJ. Bisphenol A and the risk of cardiometabolic disorders: a systematic review with meta-analysis of the epidemiological evidence. Environ Health 2015;14:46. 26026606 10.1186/s12940-015-0036-5 PMC4472611

[dmae025-B127] Rienzi L , Balaban B, Ebner T, Mandelbaum J. The oocyte. Hum Reprod 2012;27:i2–i21. 22811312 10.1093/humrep/des200

[dmae025-B128] Rivera OE , Varayoud J, Rodríguez HA, Muñoz-de-Toro M, Luque EH. Neonatal exposure to Bisphenol A or diethylstilbestrol alters the ovarian follicular dynamics in the lamb. Reprod Toxicol 2011;32:304–312. 21722727 10.1016/j.reprotox.2011.06.118

[dmae025-B129] Rochester JR , Bolden AL. Bisphenol S and F: a systematic review and comparison of the hormonal activity of Bisphenol A substitutes. Environ Health Perspect 2015;123:643–650. 25775505 10.1289/ehp.1408989 PMC4492270

[dmae025-B130] Rodriguez HA , Santambrosio N, Santamaria CG, Munoz-de-Toro M, Luque EH. Neonatal exposure to Bisphenol A reduces the pool of primordial follicles in the rat ovary. Reprod Toxicol 2010;30:550–557. 20692330 10.1016/j.reprotox.2010.07.008

[dmae025-B05191405] Rodríguez HA, , Santambrosio N, , Santamaría CG, , Muñoz-De-Toro M, , Luque EH. Neonatal exposure to bisphenol A reduces the pool of primordial follicles in the rat ovary. Reprod Toxicol 2010;30:550–557. 20692330 10.1016/j.reprotox.2010.07.008

[dmae025-B131] Ruiz TFR , Grigio V, Ferrato LJ, de Souza LG, Colleta SJ, Amaro GM, Goes RM, Vilamaior PSL, Leonel ECR, Taboga SR. Impairment of steroidogenesis and follicle development after Bisphenol A exposure during pregnancy and lactation in the ovaries of Mongolian gerbils aged females. Mol Cell Endocrinol 2023;566–567:111892. 10.1016/j.mce.2023.111892 36813021

[dmae025-B132] Ryu D-Y , Pang W-K, Adegoke EO, Rahman MS, Park Y-J, Pang M-G. Bisphenol-A disturbs hormonal levels and testis mitochondrial activity, reducing male fertility. Hum Reprod Open 2023;2023:hoad044. 38021376 10.1093/hropen/hoad044 PMC10681812

[dmae025-B133] Sabry R , Apps C, Reiter-Saunders JA, Saleh AC, Balachandran S, St John EJ, Favetta LA. BPA and BPS affect connexin 37 in bovine cumulus cells. Genes (Basel) 2021a;12:321. 33672423 10.3390/genes12020321 PMC7926832

[dmae025-B134] Sabry R , Saleh AC, Stalker L, LaMarre J, Favetta LA. Effects of Bisphenol A and Bisphenol S on microRNA expression during bovine (*Bos taurus*) oocyte maturation and early embryo development. Reprod Toxicol2021b;99:96–108. 33285269 10.1016/j.reprotox.2020.12.001

[dmae025-B135] Saleh AC , Sabry R, Mastromonaco GF, Favetta LA. BPA and BPS affect the expression of anti-Mullerian hormone (AMH) and its receptor during bovine oocyte maturation and early embryo development. Reprod Biol Endocrinol 2021;19:119. 34344364 10.1186/s12958-021-00773-6 PMC8330045

[dmae025-B136] Sanchis Y , Coscolla C, Corpas-Burgos F, Vento M, Gormaz M, Yusà V; Bettermilk Project Biomonitoring of bisphenols A, F, S and parabens in urine of breastfeeding mothers: exposure and risk assessment. Environ Res 2020;185:109481. 32278926 10.1016/j.envres.2020.109481

[dmae025-B137] Santamaría C , Durando M, Muñoz De Toro M, Luque EH, Rodriguez HA. Ovarian dysfunctions in adult female rat offspring born to mothers perinatally exposed to low doses of Bisphenol A. J Steroid Biochem Mol Biol 2016;158:220–230. 26658420 10.1016/j.jsbmb.2015.11.016

[dmae025-B138] Santangeli S , Maradonna F, Olivotto I, Piccinetti CC, Gioacchini G, Carnevali O. Effects of BPA on female reproductive function: the involvement of epigenetic mechanism. Gen Comp Endocrinol 2017;245:122–126. 27591071 10.1016/j.ygcen.2016.08.010

[dmae025-B139] Sarigiannis DA , Karakitsios SP, Handakas E, Simou K, Solomou E, Gotti A. Integrated exposure and risk characterization of Bisphenol-A in Europe. Food Chem Toxicol 2016;98:134–147. 27769850 10.1016/j.fct.2016.10.017

[dmae025-B140] Sasso AF , Pirow R, Andra SS, Church R, Nachman RM, Linke S, Kapraun DF, Schurman SH, Arora M, Thayer KA et al Pharmacokinetics of Bisphenol A in humans following dermal administration. Environ Int 2020;144:106031. 32798798 10.1016/j.envint.2020.106031 PMC9210257

[dmae025-B141] Shen J , Kang QM, Mao YC, Yuan M, Le F, Yang XY, Xu XR, Jin F. Urinary Bisphenol A concentration is correlated with poorer oocyte retrieval and embryo implantation outcomes in patients with tubal factor infertility undergoing in vitro fertilisation. Ecotoxicol Environ Saf 2020;187:109816. 31648075 10.1016/j.ecoenv.2019.109816

[dmae025-B142] Signorile PG , Spugnini EP, Citro G, Viceconte R, Vincenzi B, Baldi F, Baldi A. Endocrine disruptors in utero cause ovarian damages linked to endometriosis. Front Biosci (Elite Ed) 2012;4:1724–1730. 22201988 10.2741/493

[dmae025-B143] Souter I , Smith KW, Dimitriadis I, Ehrlich S, Williams PL, Calafat AM, Hauser R. The association of Bisphenol-A urinary concentrations with antral follicle counts and other measures of ovarian reserve in women undergoing infertility treatments. Reprod Toxicol 2013;42:224–231. 24100206 10.1016/j.reprotox.2013.09.008 PMC4383527

[dmae025-B144] Susiarjo M , Hassold TJ, Freeman E, Hunt PA. Bisphenol A exposure in utero disrupts early oogenesis in the mouse. PLoS Genet 2007;3:e5. 17222059 10.1371/journal.pgen.0030005 PMC1781485

[dmae025-B145] Taylor JA , Vom Saal FS, Welshons WV, Drury B, Rottinghaus G, Hunt PA, Toutain P-L, Laffont CM, VandeVoort CA. Similarity of Bisphenol A pharmacokinetics in rhesus monkeys and mice: relevance for human exposure. Environ Health Perspect 2011;119:422–430. 20855240 10.1289/ehp.1002514 PMC3080921

[dmae025-B146] Telfer EE , McLaughlin M. Natural history of the mammalian oocyte. Reprod Biomed Online 2007;15:288–295. 17854526 10.1016/s1472-6483(10)60341-0

[dmae025-B147] Thayer KA , Doerge DR, Hunt D, Schurman SH, Twaddle NC, Churchwell MI, Garantziotis S, Kissling GE, Easterling MR, Bucher JR et al Pharmacokinetics of Bisphenol A in humans following a single oral administration. Environ Int 2015;83:107–115. 26115537 10.1016/j.envint.2015.06.008 PMC4545316

[dmae025-B148] Trapphoff T , Heiligentag M, El Hajj N, Haaf T, Eichenlaub-Ritter U. Chronic exposure to a low concentration of Bisphenol A during follicle culture affects the epigenetic status of germinal vesicles and metaphase II oocytes. Fertil Steril 2013;100:1758–1767.e1. 24034936 10.1016/j.fertnstert.2013.08.021

[dmae025-B149] Tricco AC , Lillie E, Zarin W, O'Brien KK, Colquhoun H, Levac D, Moher D, Peters MDJ, Horsley T, Weeks L et al PRISMA extension for scoping reviews (PRISMA-ScR): checklist and explanation. Ann Intern Med 2018;169:467–473. 30178033 10.7326/M18-0850

[dmae025-B150] Tyl RW , Myers CB, Marr MC, Sloan CS, Castillo NP, Veselica MM, Seely JC, Dimond SS, Van Miller JP, Shiotsuka RN et al Two-generation reproductive toxicity study of dietary Bisphenol A in CD-1 (Swiss) mice. Toxicol Sci 2008;104:362–384. 18445619 10.1093/toxsci/kfn084

[dmae025-B151] Tyl RW , Myers CB, Marr MC, Thomas BF, Keimowitz AR, Brine DR, Veselica MM, Fail PA, Chang TY, Seely JC et al Three-generation reproductive toxicity study of dietary Bisphenol A in CD Sprague-Dawley rats. Toxicol Sci 2002;68:121–146. 12075117 10.1093/toxsci/68.1.121

[dmae025-B152] U.S. Food and Drug Administration. Guidance for industry and other stakeholders toxicological principles for the safety assessment of food ingredients: redbook 2000. College Park, MD. 2000; revised 2007. https://www.fda.gov/media/79074/download (4 June 2024, date last accessed).

[dmae025-B153] Vandenberg LN. Non-monotonic dose responses in studies of endocrine disrupting chemicals: Bisphenol A as a case study. Dose Response 2014;12:259–276. 24910584 10.2203/dose-response.13-020.Vandenberg PMC4036398

[dmae025-B154] Vandenberg LN , Chahoud I, Heindel JJ, Padmanabhan V, Paumgartten FJR, Schoenfelder G. Urinary, circulating, and tissue biomonitoring studies indicate widespread exposure to Bisphenol A. Environ Health Perspect 2010;118:1055–1070. 20338858 10.1289/ehp.0901716 PMC2920080

[dmae025-B155] Vandenberg LN , Colborn T, Hayes TB, Heindel JJ, Jacobs DR Jr, Lee D-H, Shioda T, Soto AM, Vom Saal FS, Welshons WV et al Hormones and endocrine-disrupting chemicals: low-dose effects and nonmonotonic dose responses. Endocr Rev 2012;33:378–455. 22419778 10.1210/er.2011-1050 PMC3365860

[dmae025-B156] Vandenberg LN , Ehrlich S, Belcher SM, Ben-Jonathan N, Dolinoy DC, Hugo ER, Hunt PA, Newbold RR, Rubin BS, Saili KS et al Low dose effects of bisphenol A. Endocrine Disruptors2013a;1:e26490.

[dmae025-B157] Vandenberg LN , Hauser R, Marcus M, Olea N, Welshons WV. Human exposure to Bisphenol A (BPA). Reprod Toxicol 2007;24:139–177. 17825522 10.1016/j.reprotox.2007.07.010

[dmae025-B158] Vandenberg LN , Hunt PA, Myers JP, Vom Saal FS. Human exposures to Bisphenol A: mismatches between data and assumptions. Rev Environ Health2013b;28:37–58. 23612528 10.1515/reveh-2012-0034

[dmae025-B159] Vignault C , Cadoret V, Jarrier-Gaillard P, Papillier P, Teteau O, Desmarchais A, Uzbekova S, Binet A, Guerif F, Elis S et al Bisphenol S impairs oestradiol secretion during in vitro basal folliculogenesis in a mono-ovulatory species model. Toxics 2022;10:437. 36006116 10.3390/toxics10080437 PMC9412475

[dmae025-B160] Vilarinho F , Sendón R, van der Kellen A, Vaz MF, Silva AS. Bisphenol A in food as a result of its migration from food packaging. Trends Food Sci Technol 2019;91:33–65.

[dmae025-B161] Völkel W , Colnot T, Csanády GA, Filser JG, Dekant W. Metabolism and kinetics of Bisphenol A in humans at low doses following oral administration. Chem Res Toxicol 2002;15:1281–1287. 12387626 10.1021/tx025548t

[dmae025-B162] Völkel W , Kiranoglu M, Fromme H. Determination of free and total Bisphenol A in human urine to assess daily uptake as a basis for a valid risk assessment. Toxicol Lett 2008;179:155–162. 18579321 10.1016/j.toxlet.2008.05.002

[dmae025-B163] Vom Saal FS , Vandenberg LN. Update on the health effects of Bisphenol A: overwhelming evidence of harm. Endocrinology 2021;162:bqaa171. 10.1210/endocr/bqaa171 PMC7846099 33516155

[dmae025-B164] Wang C , He C, Xu S, Gao Y, Wang K, Liang M, Hu K. Bisphenol A triggers apoptosis in mouse pre-antral follicle granulosa cells via oxidative stress. J Ovarian Res 2024;17:20. 38229135 10.1186/s13048-023-01322-y PMC10790560

[dmae025-B165] Wang T , Han J, Duan X, Xiong B, Cui XS, Kim NH, Liu HL, Sun SC. The toxic effects and possible mechanisms of Bisphenol A on oocyte maturation of porcine in vitro. Oncotarget 2016;7:32554–32565. 27086915 10.18632/oncotarget.8689 PMC5078033

[dmae025-B166] Wang X , Jiang SW, Wang L, Sun Y, Xu F, He H, Wang S, Zhang Z, Pan X. Interfering effects of Bisphenol A on in vitro growth of preantral follicles and maturation of oocyes. Clin Chim Acta 2018;485:119–125. 29958887 10.1016/j.cca.2018.06.041

[dmae025-B167] Wetherill YB , Akingbemi BT, Kanno J, McLachlan JA, Nadal A, Sonnenschein C, Watson CS, Zoeller RT, Belcher SM. In vitro molecular mechanisms of Bisphenol A action. Reprod Toxicol 2007;24:178–198. 17628395 10.1016/j.reprotox.2007.05.010

[dmae025-B168] Yang CZ , Yaniger SI, Jordan VC, Klein DJ, Bittner GD. Most plastic products release estrogenic chemicals: a potential health problem that can be solved. Environ Health Perspect 2011;119:989–996. 21367689 10.1289/ehp.1003220 PMC3222987

[dmae025-B169] Yang L , Baumann C, De La Fuente R, Viveiros MM. Mechanisms underlying disruption of oocyte spindle stability by bisphenol compounds. Reproduction 2020;159:383–396. 31990668 10.1530/REP-19-0494 PMC7032969

[dmae025-B170] Ye X , Pierik FH, Hauser R, Duty S, Angerer J, Park MM, Burdorf A, Hofman A, Jaddoe VWV, Mackenbach JP et al Urinary metabolite concentrations of organophosphorous pesticides, Bisphenol A, and phthalates among pregnant women in Rotterdam, the Netherlands: The Generation R study. Environ Res 2008;108:260–267. 18774129 10.1016/j.envres.2008.07.014 PMC2628162

[dmae025-B171] Yenigül NN , Dilbaz S, Dilbaz B, Kaplanoğlu İ, Güçel F, Aldemir O, Baser E, Ozelci R, Moraloglu Tekin O. The effect of plastic bottled water consumption on outcomes of ICSI cycles undertaken for unexplained infertility. Reprod Biomed Online 2021;43:91–99. 34001442 10.1016/j.rbmo.2021.04.010

[dmae025-B172] Yue H , Tian Y, Wu X, Yang X, Xu P, Zhu H, Sang N. Exploration of the damage and mechanisms of BPS exposure on the uterus and ovary of adult female mice. Sci Total Environ2023a;868:161660. 36690098 10.1016/j.scitotenv.2023.161660

[dmae025-B173] Yue HF , Yang XW, Wu XY, Tian YC, Xu PC, Sang N. Identification of risk for ovarian disease enhanced by BPB or BPAF exposure. Environ Pollut2023b;319:120980. 36587784 10.1016/j.envpol.2022.120980

[dmae025-B7736073] Žalmanová T, , Hošková K, , Nevoral JAN, , Adámková K, , Kott T, , Šulc M, , Kotíková Z, , Prokešová Š, , Jílek F, , Králíčková M et al Bisphenol S negatively affects the meiotic maturation of pig oocytes. Sci Rep 2017;7:485. 28352085 10.1038/s41598-017-00570-5 PMC5428703

[dmae025-B08915440] Žalmanová T, , Hošková K, , Prokešová Š, , Nevoral JAN, , Ješeta M, , Benc M, , Yi Y-J, , Moravec J, , Močáryová B, , Martínková S et al The bisphenol S contamination level observed in human follicular fluid affects the development of porcine oocytes. Front Cell Dev Biol 2023;11:1145182. 37091980 10.3389/fcell.2023.1145182 PMC10115966

[dmae025-B174] Zhang HQ , Zhang XF, Zhang LJ, Chao HH, Pan B, Feng YM, Li L, Sun XF, Shen W. Fetal exposure to Bisphenol A affects the primordial follicle formation by inhibiting the meiotic progression of oocytes. Mol Biol Rep2012a;39:5651–5657. 22187349 10.1007/s11033-011-1372-3

[dmae025-B179] Zhang XF , Zhang LJ, Feng YN, Chen B, Feng YM, Liang GJ, Li L, Shen W. Bisphenol A exposure modifies DNA methylation of imprint genes in mouse fetal germ cells. Mol Biol Rep2012b;39:8621–8628. 22699882 10.1007/s11033-012-1716-7

[dmae025-B175] Zhang MQ , Dai XX, Lu YJ, Miao YL, Zhou CY, Cui ZK, Liu HL, Xiong B. Melatonin protects oocyte quality from Bisphenol A-induced deterioration in the mouse. J Pineal Res 2017;62:e12396. 10.1111/jpi.12396 28178360

[dmae025-B176] Zhang SX , Ding ZM, Ahmad MJ, Wang YS, Duan ZQ, Miao YL, Xiong JJ, Huo LJ. Bisphenol B exposure disrupts mouse oocyte meiotic maturation in vitro through affecting spindle assembly and chromosome alignment. Front Cell Dev Biol2020a;8:616771. 33392205 10.3389/fcell.2020.616771 PMC7773771

[dmae025-B177] Zhang MY , Tian Y, Yan ZH, Li WD, Zang CJ, Li L, Sun XF, Shen W, Cheng SF. Maternal Bisphenol S exposure affects the reproductive capacity of F1 and F2 offspring in mice. Environ Pollut2020b;267:115382. 32866863 10.1016/j.envpol.2020.115382

[dmae025-B178] Zhang T , Li L, Qin XS, Zhou Y, Zhang XF, Wang LQ, De Felici M, Chen H, Qin GQ, Shen W. Di-(2-ethylhexyl) phthalate and Bisphenol A exposure impairs mouse primordial follicle assembly in vitro. Environ Mol Mutagen 2014;55:343–353. 24458533 10.1002/em.21847

[dmae025-B180] Zhang Z , Alomirah H, Cho H-S, Li Y-F, Liao C, Minh TB, Mohd MA, Nakata H, Ren N, Kannan K. Urinary Bisphenol A concentrations and their implications for human exposure in several Asian countries. Environ Sci Technol 2011;45:7044–7050. 21732633 10.1021/es200976k

[dmae025-B181] Zhao Q , Ma Y, Sun NX, Ye C, Zhang Q, Sun SH, Xu C, Wang F, Li W. Exposure to Bisphenol A at physiological concentrations observed in Chinese children promotes primordial follicle growth through the PI3K/Akt pathway in an ovarian culture system. Toxicol In Vitro 2014;28:1424–1429. 25108129 10.1016/j.tiv.2014.07.009

[dmae025-B182] Zhou CQ , Wang W, Peretz J, Flaws JA. Bisphenol A exposure inhibits germ cell nest breakdown by reducing apoptosis in cultured neonatal mouse ovaries. Reprod Toxicol 2015;57:87–99. 26049153 10.1016/j.reprotox.2015.05.012 PMC4550517

[dmae025-B183] Zhou W , Fang F, Zhu WT, Chen ZJ, Du YZ, Zhang J. Bisphenol A and ovarian reserve among infertile women with polycystic ovarian syndrome. Int J Environ Res Public Health 2017;14:18. 10.3390/ijerph14010018 PMC5295269 28036005

[dmae025-B89258987] Zhu X, , Tian GG, , Yu B, , Yang Y, , Wu JI. Effects of bisphenol A on ovarian follicular development and female germline stem cells. Arch Toxicol 2018;92:1581–1591. 29380011 10.1007/s00204-018-2167-2

[dmae025-B184] Ziv-Gal A , Craig ZR, Wang W, Flaws JA. Bisphenol A inhibits cultured mouse ovarian follicle growth partially via the aryl hydrocarbon receptor signaling pathway. Reprod Toxicol 2013;42:58–67. 23928317 10.1016/j.reprotox.2013.07.022 PMC3836856

